# Effects of whole-body vibration training as an adjunct to conventional rehabilitation exercise on pain, physical function and disability in knee osteoarthritis: A systematic review and meta-analysis

**DOI:** 10.1371/journal.pone.0318635

**Published:** 2025-02-10

**Authors:** Yan Peng, Qi Qi, Chai Li Lee, Yan Ling Tay, Siaw Chui Chai, Mohd Azzuan Ahmad

**Affiliations:** 1 Physiotherapy Programme, Centre for Rehabilitation and Special Needs Studies, Faculty of Health Sciences, Universiti Kebangsaan Malaysia, Kuala Lumpur, Malaysia; 2 Shanghai Yangzhi Rehabilitation Hospital (Shanghai Sunshine Rehabilitation Center), Shanghai, China; 3 Occupational Therapy Programme, Centre for Rehabilitation and Special Needs Studies, Faculty of Health Sciences, Universiti Kebangsaan Malaysia, Kuala Lumpur, Malaysia; Berner Fachhochschule, SWITZERLAND

## Abstract

**Background:**

Knee osteoarthritis (KOA) is a prevalent degenerative joint condition that impairs mobility and quality of life. While whole-body vibration training (WBVT) shows promise as an adjunct to conventional KOA rehabilitation, its efficacy remains unclear due to inconsistent clinical evidence.

**Objective:**

To elucidate the combined effects of WBVT and rehabilitation exercise on pain, physical function, and disability in KOA management through a systematic review and meta-analysis.

**Methods:**

A comprehensive search was conducted across eight electronic databases (PubMed, Web of Science, Embase, PEDro, SPORTDiscus, Scopus, ScienceDirect, and China National Knowledge Infrastructure) up to February 2024. Inclusion criteria were (i) randomized controlled trials comparing combined WBVT and rehabilitation exercise versus rehabilitation alone in KOA (ii) reported clinical outcomes (iii) human studies, and (iv) publications in English or Chinese. Trial quality was assessed using the PEDro scale and Cochrane risk-of-bias tool. The meta-analysis employed random-effects models in Review Manager 5.3 to account for heterogeneity, supported by sensitivity analyses for robustness and subgroup analyses on WBVT frequency effects.

**Results:**

Sixteen RCTs comprising 589 participants were included. The systematic review found that WBVT combined with conventional rehabilitation significantly reduced pain and improved physical function in KOA patients. The meta-analysis quantified these effects, showing that WBVT significantly (i) reduced knee pain (MD = −0.43, 95% CI [−0.70, −0.16], *p* = 0.002), with greater reductions observed from high-frequency WBVT, and (ii) increased isokinetic knee peak torque compared to rehabilitation exercise alone. No significant differences were found in balance, functional mobility, and disability outcomes. Sensitivity analysis of high-quality trials supported these results. However, the heterogeneity among studies and variations in control group interventions warrant cautious interpretation.

**Conclusion:**

WBVT seems to be effective in reducing pain and enhancing muscle strength in KOA patients when used in conjunction with conventional rehabilitation. Future high-quality RCTs must standardize WBVT protocols, emphasize long-term follow-up, and refine dosage for clinically meaningful outcomes.

**Systematic review registration:** International prospective register of systematic reviews (PROSPERO CRD42024508386)

## Introduction

Knee osteoarthritis (KOA) is a chronic degenerative joint disease characterized by the progressive degradation of articular cartilage primarily due to wear and tear [1]. Clinically, KOA manifests as knee pain, joint swelling, limited mobility, stiffness, and functional impairment [2,3], severely impacting daily activities and quality of life [1]. Globally, KOA is a prevalent form of osteoarthritis, with a reported prevalence of 16.0% and an incidence rate of 203 per 10,000 person-years, highlighting a significant public health concern [4]. It affects over 650 million individuals over 40 years old, with a notable 50% prevalence in those over 65 years, including 13% in women and 10% in men [5]. KOA is a major health challenge, ranked among the top five causes of disability worldwide [6]. Its rising prevalence imposes a substantial burden on individuals, healthcare systems, and society, necessitating effective management strategies [7]. Despite various conservative treatments such as medication, therapeutic exercises, and electrophysical agents, achieving optimal outcomes in KOA management remains challenging, indicating that these interventions alone are often insufficient [7,8].

Recently, whole-body vibration training (WBVT) has gained significant attention in the rehabilitation of various musculoskeletal disorders [9,10]. Rehabilitation, in this context, is a multifaceted process aimed at restoring function across different domains. It includes the restoration of physiological functions of body systems (e.g., musculoskeletal and neuromuscular systems), improving the ability to perform specific tasks or activities (e.g., walking, climbing stairs), and enhancing participation at individual and societal levels (e.g., work, social integration). For patients with KOA, rehabilitation focuses on alleviating pain, enhancing physical function, and supporting a return to functional independence, ultimately improving quality of life. WBVT utilizes motor-driven vibrating platforms to transmit energy through the body, inducing changes in the muscle-tendon complex and eliciting reflexive muscle contractions [11]. Three types of WBV devices are commonly used in clinical, training, and research settings [12,13]. These include sinusoidal vertical WBV (VS-WBV), which operates at frequencies between 30–60 Hz and amplitudes of 0−12 mm (both feet placed on one plate); sinusoidal side-alternating WBV (SS-WBV), which operates at frequencies between 12–30 Hz and amplitudes of 0–12 mm (feet placed on one plate); and stochastic resonance WBV (SR-WBV), characterized by frequencies of 1–12 Hz and amplitudes of 0–12 mm (each foot placed on independent motorized plates) [12,13].

The physiological mechanisms of WBVT are diverse and encompass several key aspects. Firstly, it involves the stimulation of rapid muscle contractions via vibration, activating both voluntary and involuntary muscle fibers, which leads to greater muscle engagement compared to traditional static exercises [14]. Secondly, WBVT promotes the recruitment and synchronization of motor units, fostering motor learning and neural adaptations, thus enhancing overall muscle performance [15,16]. Thirdly, it has been observed to elevate growth hormone levels while reducing cortisol levels, indicating potential hormonal benefits [17]. Lastly, WBVT imposes mechanical stress on bones, which can stimulate bone remodeling processes [18]. Moreover, WBVT is reported for its ability to enhance muscle strength and proprioception by activating muscle proprioceptors and increasing myocyte content [11].

The application of WBVT in musculoskeletal disorders, including KOA, has been gradually explored [10]. Despite growing interest, the clinical efficacy of WBVT remains uncertain due to conflicting findings from recent clinical trials. Some studies have demonstrated positive effects on pain [10], physical function, and disability [19], while others have reported no significant differences compared to conventional exercise interventions [20,21]. Early meta-analyses by Wang et al. (2015) [22], Li et al. (2015) [23], and Zafar et al. (2015) [24] included a limited number of studies, rendering their findings inconclusive. The most recent comprehensive review by Qiu et al. (2022) analyzed 14 trials from five databases, indicating that WBVT had additional positive effects on pain, knee extensor strength, and physical function in KOA [9]. However, the control groups across the individual trials exhibited significant variations, and the review did not specifically address the effect of rehabilitation exercise, which is the primary recommended conservative treatment [7,25,26]. These variations in control groups may influence the outcomes, particularly in meta-analyses [27,28]. This oversight is significant, as the combination of WBVT and rehabilitation exercises might offer synergistic benefits not apparent when either therapy is used alone [20]. Further research is needed to clarify the efficacy of WBVT, particularly in combination with established rehabilitation exercises, to provide more definitive clinical guidelines.

This study aims to address the aforementioned gaps by conducting a systematic review and meta-analysis to elucidate the combined effects of WBVT and conventional rehabilitation exercises on pain, physical function, and disability in patients with KOA. This research represents a methodological advancement by delineating the role of WBVT as an adjunct to conventional rehabilitation exercises and undertaking a comprehensive search across multiple databases, including both English and Chinese publications. By synthesizing the existing literature on this integrated approach, we seek to provide robust insights into the synergistic effects of WBVT and conventional rehabilitation exercises, potentially offering valuable guidance for healthcare practitioners in optimizing KOA rehabilitation strategies.

## Materials and methods

This systematic review was conducted in accordance with the PRISMA guidelines (S1 File) [29]. The protocol has been registered with PROSPERO (CRD42024508386). The review question was formulated using the population, intervention, comparison, and outcome (PICO) model: “What are the effects of WBVT combined with conventional rehabilitation exercises on pain, physical function, and disability in adults with KOA?”

### Search strategy and data sources

A comprehensive search of relevant literature, from inception regardless of publication year, was conducted across eight electronic databases: PubMed, Web of Science (WOS), Embase, PEDro, SPORTDiscus, Scopus, ScienceDirect, and China National Knowledge Infrastructure (CNKI). The final database searches were conducted on February 29, 2024. The keywords used for the search were “vibration,” “whole body vibration,” “WBV,” “knee osteoarthritis,” “gonarthrosis,” and “arthr*.” To broaden the systematic search, Boolean operators “AND” and “OR” were utilized to combine these keywords. The search strategy chosen was “whole body vibration AND knee osteoarthritis,” due to its highest hit rate and relevance. Furthermore, cross-referencing of the retrieved articles was performed to identify any additional relevant studies.

During the course of the review, minor deviations from the registered PROSPERO protocol (CRD42024508386) were made to enhance the comprehensiveness of the search. Initially, the database search was planned to conclude by January 2024, but this was extended to February 2024 to ensure the inclusion of the most up-to-date studies. Additionally, while the original protocol specified the inclusion of English-language articles only, the language criteria were expanded to include both English and Chinese publications. This allowed for the capture of relevant studies from Chinese databases, particularly those exploring KOA interventions. Lastly, China National Knowledge Infrastructure was included as an additional database, increasing the total number of databases searched from seven to eight. These adjustments were implemented to improve the coverage and comprehensiveness of the review while maintaining alignment with the original objectives and methodological rigor.

### Study selection criteria

The literature for this review was selected based on the following eligibility criteria: (1) randomized controlled trials assessing the effectiveness of WBVT combined with rehabilitation exercises for KOA compared to a control group; (2) studies involving adults diagnosed with KOA according to the American College of Rheumatology criteria; (3) participants in the experimental group who received WBVT combined with conventional rehabilitation exercises; (4) control group participants who received rehabilitation exercises; (5) studies reported clinical outcomes including knee pain, physical function, and disability scores; (6) studies were included regardless of the year of publication; (7) studies involving humans only; and (8) studies published in English or Chinese. Exclusions were made for review articles, letters to the editor, and animal studies. Two authors independently conducted database searches and selected trials based on these criteria. Disagreements in selection were resolved through discussion, and if necessary, a consensus-based decision involving a third author was reached.

### Risk of bias and quality assessment

The methodological quality of the chosen studies was independently evaluated by two authors using the PEDro scale and the Cochrane risk-of-bias tool. Any discrepancies in item scores were resolved through discussion between the two reviewers until a consensus was achieved. The PEDro scale is particularly relevant for physiotherapy and rehabilitation research, providing a comprehensive assessment of the quality of randomized controlled trials with a focus on internal validity, statistical reporting, and study design. Based on the PEDro scale, studies scoring six or above were deemed to be of high methodological quality with a low risk of bias, whereas those scoring three or below were considered low quality with a high risk of bias [30]. The Cochrane risk-of-bias assessment covers the following domains: randomization method, allocation concealment, blinding (participant and evaluator), completeness of outcome data, selective reporting of findings, and other sources of bias. Judgment options for each domain included “low risk,” “unclear risk,” and “high risk.”

### Data extraction

The information from the selected studies was independently extracted by two authors. The extracted data encompassed several categories: (1) study details, (2) participant demographics (including age, gender, body mass index, and Kellgren–Lawrence grading system), (3) details of interventions (WBVT and rehabilitation exercise), (4) scores reflecting clinical outcomes, and (5) primary findings. Discrepancies in the data collected by the two authors were reconciled through discussion involving a third author to reach a consensus. The outcomes selected for meta-analysis were based on the availability of reported data and included (i) knee pain intensity scores as measured by visual analogue scale (VAS) and numerical pain rating scale (NPRS), (ii) physical function assessed using the Timed Up and Go Test (TUGT), 6-minute Walk Distance test (6MWT), muscle strength, 30-second chair stand test (CST), and Berg Balance Scale (BBS), and (iii) disability score as evaluated using the McMaster Universities Osteoarthritis Index (WOMAC).

In cases where studies did not report sufficient data (e.g., missing means or standard deviations), attempts were made to contact the study authors for clarification. If no response was obtained, missing data were estimated using established methods, such as imputing missing standard deviations from available data (e.g., using the largest or smallest reported values) or calculating them from confidence intervals, t-values, or *p*-values when available. Advanced imputation techniques (e.g., multiple imputation) were not utilized due to the limited availability of individual participant data from the included studies. Sensitivity analyses were performed to assess the robustness of the results.

### Statistical analysis

Participant characteristics at the group level were analyzed using SPSS Statistics software version 28 (IBM, USA). The meta-analysis primarily focused on key outcomes: knee pain intensity, physical function, and disability level scores, utilizing post-intervention data (immediately upon intervention completion) (S2 File). Due to variations in data reporting across studies, robust techniques were employed to estimate the mean (SD) where necessary. Since the clinical outcomes were continuous, the mean and standard deviation (SD) were used to calculate the standard mean difference (SMD), with a 95% confidence interval (CI). To ensure uniformity, robust techniques were consistently applied to estimate mean (SD) values throughout the meta-analysis.

The meta-analyses were performed by two authors, with a third author reviewing the results to resolve any disagreements. The primary meta-analysis was conducted using Review Manager software 5.3 (The Nordic Cochrane Centre), employing random-effects models to account for expected heterogeneity among the included studies, especially when significant heterogeneity (*I*² ≥ 50%) [31]. Subgroup and sensitivity analyses were conducted to explore potential sources of heterogeneity. The significance level was set at *p* < 0.05, and forest plots were generated to visually represent the meta-analysis results.

## Results

### Data screening, selection, and extraction process

**Fig 1:**
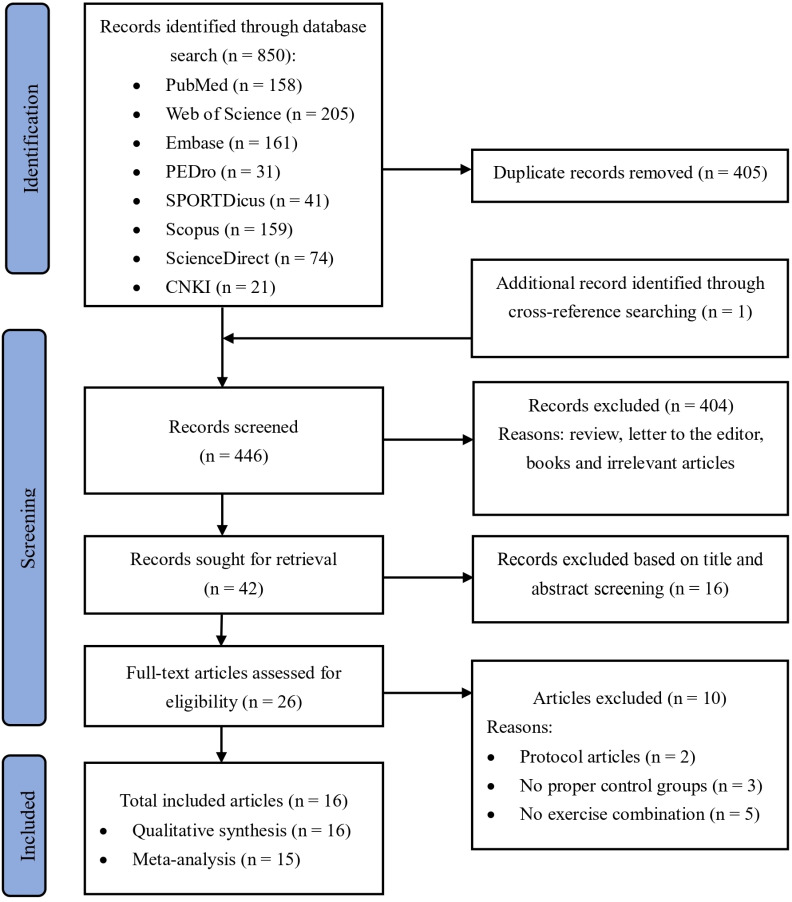
Flowchart depicting the study selection process.

A total of 850 articles were identified from eight databases: PubMed (n = 158), Web of Science (n = 205), Embase (n = 161), PEDro (n = 31), SPORTDiscus (n = 41), Scopus (n = 159), ScienceDirect (n = 74), and CNKI (n = 21). After initial screening, 405 articles were removed due to duplication. An additional article was identified through cross-referencing (n = 1), and 403 articles were excluded as they were reviews, letters, books, or irrelevant. Screening of titles and abstracts excluded 16 studies, leaving 26 articles for full-text review. Following the full-text review, 10 trials were excluded for the following reasons: protocol articles (n = 2), lack of proper control groups (n = 3), and absence of rehabilitation exercise combination (n = 5) (S1 Table). As a result, 16 trials were included in the review. Fig 1 presents a flowchart of the study selection process.

### Study characteristics

This review analysed 16 studies with a total of 589 participants (310 participants in the experimental group and 279 in the control group), published between 2011 and 2021. Participant ages ranged from 51.94 to 75.12 years. The studies originated from various countries: China (n = 6) [22,32–35], Brazil (n = 3) (30-32), Iran (n = 2) [21,36], India (n = 2) [20,37], United States (n = 1) [38], Korea (n = 1) [39], and Japan (n = 1) [40]. The studies varied in sample sizes from 15 [34] to 99 [22,32–35] participants, with an average of 37 participants. Detailed characteristics of the included studies are summarized in Table 1.

**Table 1:** Summary of the included studies.

Study; Design; PEDro score	Groups (n); Age, years: mean (SD); Sex, Male/Female (n); KL grade, (I/ II/ III/ IV)	Details intervention of the whole-body vibration training; Details intervention of the rehabilitation exercise	Findings of the experimental group: Significant improvement in pre-post assessment (Yes/ No)		
			Outcome measures	Intra-group	Inter-group
Abbasi et al. (2017); RCT-SB; 6	Experimental (n = 12); Age: 63.69 (3.05); Sex: 6/6; Grade: II & III Control (n = 13); Age: 63.5 (4.84); Sex: 8/5; Grade: II & III	Amplitude: 3 mm, frequency: 30 Hz; barefoot on platform, 30-degree knee flexion (semi-squat); 12 sessions (3x/week for 1 month) Static quadriceps contractions, straight leg raises, 3 sets of 10 reps with 5s hold; 12 sessions (3x/week for 1 month)	Strength: EMG (semi squat)	No	No
			Strength: EMG (SLR position)	No	No
			Strength: EMG (ankle plantar flexion position)	Yes	Yes
Aggarwal et al. (2020); RCT; 4	Experimental (n = 15); Age: 59.00 (5.68); Sex: 7/8; Grade: I, II & III Control (n = 15); Age: 62.00 (5.88); Sex: 2/13; Grade: I, II & III	Frequency: 25 Hz, rest intervals increased from 30s to 60s; repetitions increased in 4th week; 12 sessions (3x/week for 4 weeks) Supervised strengthening exercises: isometric quadriceps, hip adduction, straight leg raises; 12 sessions (3x/week for 4 weeks)	Pain: VAS	Yes	No
			ROM: Knee active flexion and extension	No	No
			Disability: WOMAC	No	No
			Balance: BBS	Yes	No
			Functional: 30-second CST	Yes	No
Avelar et al. (2011); RCT; 5	Experimental (n = 11); Age: 75.00 (5.00); Sex: 2/9; Grade: I, II, III & Ⅳ Control (n = 10); Age: 71.00 (4.00); Sex: 1/9; Grade: I, II, III & Ⅳ	Frequency: 35-40 Hz, amplitude: 4 mm; 36 sessions (3x/week for 12 weeks) Supervised warm-up on ergometric bicycle at 70% of predicted max heart rate for 10 minutes; 36 sessions (3x/week for 12 weeks)	Functional: 30-second CST	Yes	No
			Disability: WOMAC	Yes	No
			Functional: 6MWT	Yes	No
			Balance: BBS	Yes	No
			Functional: TUG	Yes	No
Bokaeian et al. (2016); RCT; 7	Experimental (n = 15); Age: 51.80 (8.30); Sex: 0/9; Grade: I, II & III Control (n = 13); Age: 54.00 (3.90); Sex: 4/9; Grade: I, II & III	Vertical vibration: 2 mm; 24 sessions (3x/week for 8 weeks) Supervised warm-up on bicycle (5 min), continuous flexion-extension on dynamometer; 24 sessions (3x/week for 8 weeks)	Pain: VAS	No	No
			Functional: 50-foot walking test (50FWT)	No	No
			Disability: WOMAC	No	No
			Functional: 2MWT	Yes	No
			Functional: TUG	No	No
			Strength: muscle peak torque (MPT), total work (TW) and muscle power (MP)	Yes	No
Lai et al. (2021); RCT-SB; 7	Experimental (n = 27); Age: 63.52 (4.98); Sex: 5/22; Grade: I, II & III Control (n = 27); Age: 64.81 (4.04); Sex: 2/25; Grade: I, II & III	Frequency: 20 Hz, amplitude: 2 mm; static squat on platform (30° and 60° knee flexion); 24 sessions (3x/week for 8 weeks) Supervised static squat on flat ground with bent knees (30° and 60°); 24 sessions (3x/week for 8 weeks)	Pain: VAS	Yes	No
			Functional: Proprioception	Yes	No
			Functional: 6MWT	No	No
			Functional: TUG	Yes	No
			Strength: isokinetic dynamometer	No	Yes
Lai et al. (2019); RCT- SB; 6	Experimental (n = 20); Age: 64.10 (4.95); Sex: 4/16; Grade: I, II & III Control (n = 21); Age: 64.10 (4.95); Sex: 1/20; Grade: I, II & III	Frequency: 20 Hz, amplitude: 2 mm; vertical vibration, static squat on platform without shoes, knee flexion (30° and 60°); 24 sessions (3x/week for 8 weeks) Supervised warm-up (5 min), static squat on flat ground; 24 sessions (3x/week for 8 weeks)	Pain: VAS	Yes	No
			Functional: TUG	Yes	No
			Functional: 6MWT	Yes	No
			Strength: isokinetic dynamometer	No	No
Park et al. (2013); RCT; 4	Experimental (n = 11); Age: 62.5 (5.66); Sex: Not specified; Grade: II & III Control (n = 11); Age: 60.0 (6.22); Sex: Not specified; Grade: II & III	Low frequency: 12-14 Hz, vertical displacement: 2.5-5 mm; barefoot on platform, slight knee flexion, 20 minutes training, 2 cycles of 10 minutes with 5-minute rest; 24 sessions (3x/week for 8 weeks) Home-based active range-of-motion exercises, muscle strengthening, up to 10 reps each; 24 sessions (3x/week for 8 weeks)	Pain: NRS	Yes	Yes
			Functional: Lysholm Scoring Scale	Yes	No
			Disability: KWOMAC	Yes	No
			Strength: isokinetic torque and isometric torque	Yes	No
			Balance: Biodex Stability System	Yes	No
Philip et al. (2018); RCT; 5	Experimental (n = 15); Age: Not specified; Sex: Not specified; Grade: Not specified Control (n = 15); Age: Not specified; Sex: Not specified; Grade: Not specified	Frequency of 45 Hz; amplitude of 4 to 6 mm displacement. Total exposure time of 30 minutes/day (vibration maximum 20 minutes, interval rest 10 minutes) on 7 days/week Conventional exercises only given for 30 min/day for 7 days/week	Pain: NRPS	Yes	Yes
			Strength: MST	Yes	Yes
			Functional: TUG	Yes	No
Segal et al. (2013); RCT-SB; 7	Experimental (n = 26); Age: 52.80 (4.90); Sex: 0/26; Grade: Not specified Control (n = 13); Age: 52.80 (4.70); Sex: 0/13; Grade: Not specified	WAVE Pro-elite vibration platform/ Amplitude of 2 mm/A frequency of 35 Hz; 24 sessions (2x/week for 12 weeks) Exercise protocol included five leg-strengthening exercises with stretches and rest between repetitions; 24 sessions (2x/week for 12 weeks)	Strength: Lower limb muscle power	Yes	No
			Function: Stair climb power	Yes	No
Simao et al. (2012); RCT-SB; 7	Experimental (n = 10); Age: 75 (7.4); Sex: 1/9; Grade: II, III & Ⅳ Control (n = 10); Age: 69 (3.7); Sex: 2/8; Grade: II, III & Ⅳ	Frequency was varied from 35 to 40Hz/ amplitude was 4mm/ acceleration ranged from 2.78 to 3.26g/ 10° of knee flexion and continuing until 60°; 36 sessions (3x/week for 12 weeks) Supervised/ The squat exercise was performed starting at approximately 10° of knee flexion and continuing until 60° of knee flexion was reached, the length of maintaining the semi full position (3s) and the flexed position (3s of isometric contraction) of the knees in each squat repetition; 36 sessions (3x/week for 12 weeks)	Disability: WOMAC	Yes	No
			Functional: 6MWT	Yes	No
			Balance: BBS	Yes	No
			Functional: gait speed	Yes	Yes
Simao et al. (2019); RCT-DB; 8	Experimental (n = 7); Age: 75.00 (68.55); Sex: 0/7; Grade: II, III & Ⅳ Control (n = 8); Age: 71 (67.7–74.3); Sex: 0/8; Grade: II, III & Ⅳ	Frequency of 35–40 Hz/ Amplitude of 4 mm/ Acceleration that ranged from 2.78 to 3.26 G/ Two feet are 28 cm apart, each lower limb is stimulated by the same amount of vibration, and squat on the vibration platform; 36 sessions (3 times per week for 12 weeks) Squats begin with knee flexion of about 10° and continue until knee flexion reaches 60°. Standardize the time the knee is held in half position (3 seconds) and bent position (3 seconds isometric contraction) for each repeat squat; 36 sessions(3 times per week for 12 weeks)	Strength: isometric quadriceps muscle strength	No	Yes
Tsuji et al. (2014); SB 4	Experimental (n = 29); Age: 62.1 (5.5); Sex: 0/29; Grade: I, II, III & Ⅳ Control (n = 9); Age: 60.9 (4.6); Sex: 0/9; Grade: I, II, III & Ⅳ	Frequency of 30 Hz/ vibration amplitude of 2.5 mm and for 30 s/set; 24 sessions (3x/week for 8 weeks) Home-based/ 50 min long which included a 10-minute warm-up and flexibility training period, a 25-minute period of strength training with participants using their own weight and a 15-minute cool-down period; 24 sessions (3x/week for 8 weeks)	Functional: TUG	No	Yes
			Disability: Japanese Knee Osteoarthritis Measure	No	Yes
			Strength: Knee strength and power	Yes	No
Wang et al (2015); RCT-DB; 9	Experimental (n = 49); Age: 61.2 (9.6); Sex: 13/ 36; Grade: II & III Control (n = 50); Age: 61.5 (9.1); Sex: 15/ 35; Grade: II & III	Frequency of 35 Hz/ amplitude of 4-mm to 6-mm displacement/ The participants were guided to stand on the vibration platform without shoes and with knees slightly flexed/ Total exposure time of 30 min per day (vibration 60 seconds, interval rest 60 seconds); 40 sessions (5x/week for 24 weeks) Supervised/ Quadriceps Resistance Exercise, static inner quadriceps contraction/ Band knee extension in sitting/ Squats with Bobath ball, 40 min per day; 40 sessions (5x/week for 24 weeks)	Pain: VAS	Yes	Yes
			Functional: ROM	Yes	Yes
			Disability: WOMAC	Yes	Yes
			Functional: TUG	Yes	Yes
			Functional: 6MWT	Yes	Yes
			Functional: SF-36	Yes	Yes
Wang et al. (2016); RCT; 9	Experimental (n = 19); Age: 61.10 (7.10); Sex: 9/ 10; Grade: I, II, III & Ⅳ Control (n = 20); Age: 61.5 (7.3); Sex: 7/ 13; Grade: I, II, III & Ⅳ	Frequency of 35 Hz/ amplitude of 4 - 6mm, total exposure time of 30 minutes/day (vibration 60 seconds, interval rest 60 seconds); 60 sessions (5x/week for 12 weeks) Supervised/ static inner quadriceps contraction; quadriceps over fulcrum resistance; band knee extension in sitting; squats with a Bobath ball; 60 sessions (5x/week for 12 weeks)	Pain: VAS	Yes	No
			Disability: WOMAC	Yes	Yes
			Functional: TUG	Yes	Yes
			Functional: 6MWT	Yes	Yes
			Functional: Gait parameters	No	No
Xia et al. (2017); RCT-DB; 6	Experimental (n = 28); Age: Not specified; Sex: Not specified; Grade: Not specified Control (n = 28); Age: Not specified; Sex: Not specified; Grade: Not specified	Frequency 6 ~ 8 Hz, amplitude 2.5 mm, torso maintained upright position, 10 min each time; 28 sessions (7x/week for 4 weeks) Patients were seated for treadmill aerobic training, the resistance of the training was the degree of slight exertion of the patient’s foot, the training time was 30 min; 28 sessions (7x/week for 4 weeks)	Pain: VAS	Yes	Yes
			Pain: PPT	Yes	No
			Pain, stiffness and function: WOMAC	Yes	No
			Function: Lysholm	Yes	Yes
Zhang et al. (2021); RCT; 5	Experimental (n = 16); Age: 60.38 (6.30); Sex: 8/8; Grade: Not specified Control (n = 16); Age: 51.94 (6.14); Sex: 7/9; Grade: Not specified	Knee flexion 0° ~ 15°, vibration frequency 35 ~ 40 Hz, vibration amplitude 4 ~ 6 mm, each treatment time 10 min; 32 sessions (4x/week for 8 weeks) Muscle strength training, range of motion training, aerobic training; 32 sessions (4x/week for 8 weeks)	Pain: VAS	Yes	No
			Disability: WOMAC	Yes	Yes
			Pain, stiffness and function: WOMAC	Yes	No
			Function: LEFS	No	No
			Function: Functional reach test	Yes	No

Note: EMG, electromyography; VAS, visual analogue scale; ROM, Range of motion; WOMAC, Western Ontario and McMaster Universities Osteoarthritis Index; 6MWT, six-minute walk test; NRPS, numerical rating pain scale; RCT, randomized controlled trial; SB, single-blinded; DB, double-blinded; TUG, timed up and go test; SF-36, MOS Item Short Form from Health Survey; BBS, Berg Balance Scale; CST, Chair Stand Test; JKOM, Japanese Knee Osteoarthritis Measure; MPT, muscle peak torque; TW, total work; MP, muscle power; 50FWT, 50-foot walking test; IQMS, isometric quadriceps muscle strength; MST, Modified Sphygmomanometer Test.

### Risk of bias and quality assessment

**Fig 2:**
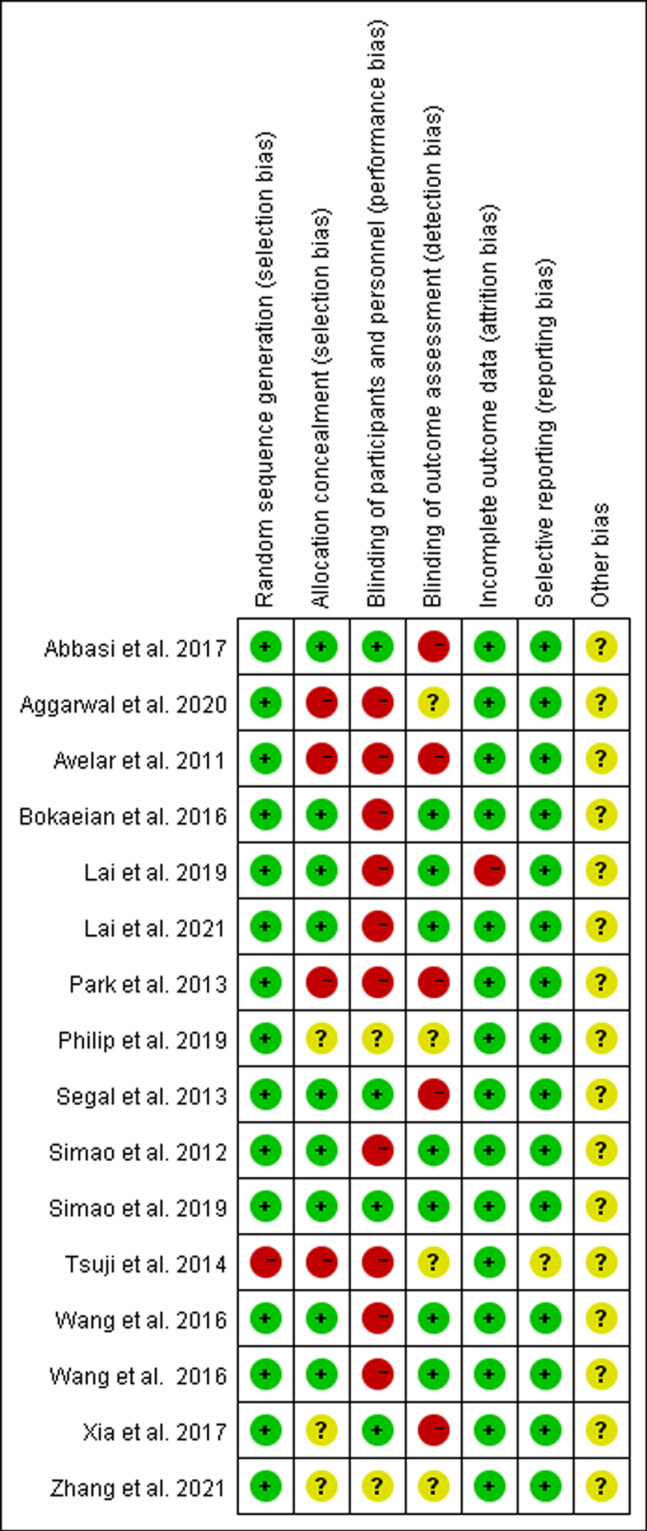
Risk-of-bias graph and summary.

The 16 studies included in this review and meta-analysis were of moderate (38%; n = 6) to high (62%; n = 10) methodological quality, with PEDro scores ranging from four to nine and a mean score of 6.1 (S2 Table). Three studies were double-blinded [34,35,41], seven were single-blinded [21,32,33,36,38,42,43], and six did not specify the blinding method [19,20,37,39,40,44]. Using the Cochrane risk of bias assessment, randomized sequence generation was rated as low risk in 15 studies [20,21,32–39,41,42,44], and high risk in one study [40] (S3 Table). Allocation concealment was low risk in nine studies [21,32–36,38,41,42], high risk in four studies [20,39,40,44], and not explicitly described in three studies [11,37,43]. Participant blinding was low risk in four studies [36,38,41,43] and high risk in 10 studies [20,21,32–35,39,40,42,44], with two studies not explicitly described [11,37]. Assessor blinding was high risk in five studies [36,38,39,43,44], low risk in seven studies [21,32–35,41,42], and not specified in the remaining studies [11,20,37,40,44]. Completeness of outcome data was low risk in 15 studies [11,20,21,33–40,42–44], and high risk in one study [32]. Selective reporting was low risk in 15 studies [11,20,21,32–39,41–44] and unclear in one study [40]. No other sources of bias were mentioned. Fig 2 illustrates the risk-of-bias for each study.

### Publication bias

**Fig 3:**
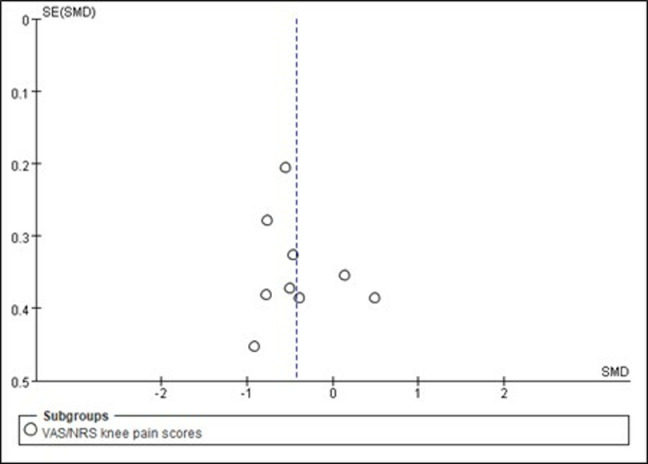
Funnel plot of publication bias risk.

To assess the potential for publication bias, a funnel plot was generated for studies reporting VAS scores, which is a primary outcome in this review. However, due to the limited number of studies included in the meta-analysis for some outcomes, the visual assessment of the funnel plot may not be sufficient to conclusively determine publication bias. Therefore, Egger’s regression test was conducted to quantitatively evaluate the risk of publication bias for VAS scores using StataMP 14.0. The results of Egger’s test (t = −0.26, *p* = 0.804) suggest no significant evidence of publication bias (*p* > 0.05) (Fig 3). This finding, supported by the symmetrical appearance of the funnel plot, indicates that publication bias is unlikely to have influenced the results for the pain outcome in this review.

### WBVT intervention

The vibration parameters in the selected studies varied (Table 1). Vibration frequencies ranged from 6 to 8 Hz to 45 Hz, amplitudes from 2 mm to 6 mm, number of motions from 12 to 60, and intervention durations from 4 to 24 weeks. Vibration frequencies were classified into high (> 30 Hz) and low (≤ 30 Hz), resulting in seven low-frequency [20,32,33,36,39,40,43] and eight high-frequency studies [11,21,35,37,38,41,42,44]; low-frequency interventions generally had shorter durations compared to high-frequency interventions. The longest WBVT intervention was 24 weeks, involving 99 participants [36]. All studies supplemented WBVT with conventional rehabilitation exercise, with the reported outcomes assessed at baseline and immediately post-treatment; except for one study [37] that assessed outcomes seven days post-treatment. Due to variations in intervention timing and assessment time points, the meta-analysis utilized outcome measurements taken immediately after treatment completion.

### Rehabilitation exercise intervention

The conventional rehabilitation exercises were prescribed as supervised [11,20,21,32–35,41–44] or a home-based program [39,40]; however, three studies did not specify the mode of exercise prescription [36–38]. Among the training programs included active range of motion exercises, static quadriceps contractions, lower limb strength training, functional training, and squatting exercises. Supervised rehabilitation exercise frequencies ranged from three to seven times per week, with most studies conducted three times per week, and the total duration ranged from four to 24 weeks. Home-based exercises entailed providing detailed handouts and instructions [39,40]. Only one study [40] included a detailed and standardized program comprising warm-up, flexibility training, strength training, and cool-down components.

### Meta-analysis results

Various outcome indicators (VAS, WOMAC, TUG, knee strength, 6MWT, BBS, and CST) were used to assess pain, physical functioning, functional mobility and disability in KOA participants. Meta-analysis included data from 15 study trials due to the unavailability of data from one study [33].

### Meta-analysis of knee pain scores

**Fig 4:**
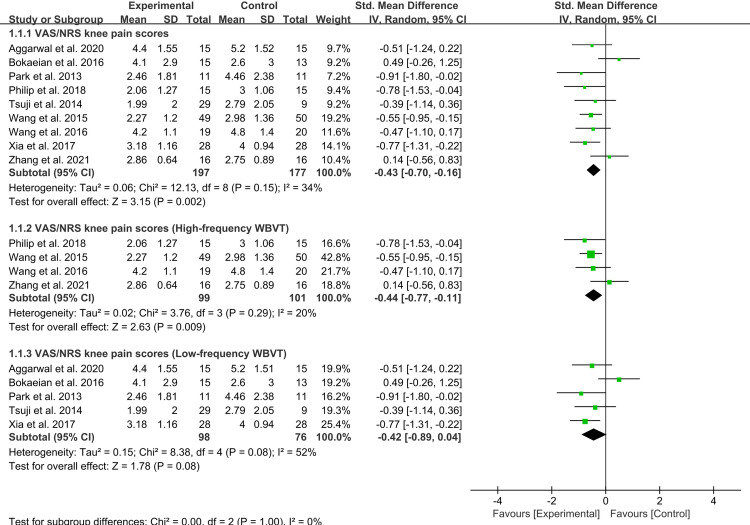
Forest plot of knee pain intensity scores for the experimental versus control groups.

Nine studies reported outcomes on knee pain intensity, covering 374 participants (197 in the experimental group and 177 in the control group). Seven studies reported VAS scores [11,20,21,34,35,40,43], while two used NPRS [37,39]. The experimental group showed a significant reduction in knee pain compared to the control group [Mean Difference (MD) = −0.43, 95% CI (−0.7, −0.16), *I*^2^ = 34%, *p* = 0.002]. Subgroup analysis revealed that high-frequency vibration training was more effective for knee pain reduction [MD = −0.44, 95% CI (−0.77, −0.11), *I*^2^ = 20%, *p* = 0.009] compared to low-frequency [MD = −0.42, 95% CI (−0.89, −0.04), *I*^2^ = 52%, *p* = 0.08] (Fig 4). However, a sensitivity analysis of four high-quality trials [21,34,35,43], each with a PEDro score of 6 or higher, found no significant difference in pain reduction between the experimental and control groups (S1 Fig).

### Meta-analysis of WOMAC scores

**Fig 5:**
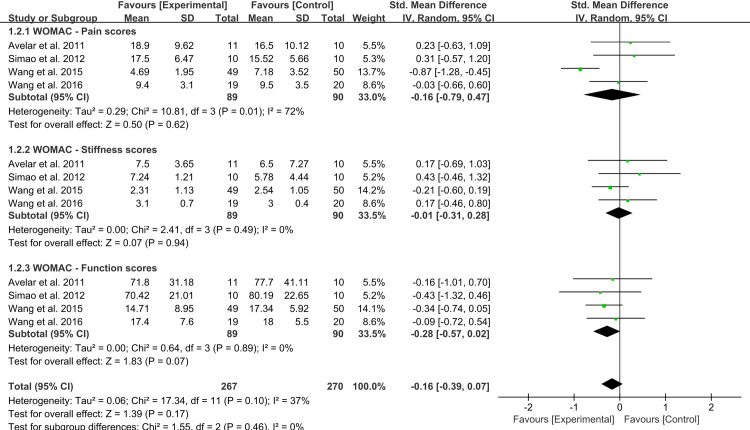
Forest plot of WOMAC scores for pain, stiffness, and function for experimental versus control groups.

Four studies (179 participants) reported WOMAC domain scores for pain, stiffness, and function. No significant difference was found between the experimental and control groups in WOMAC-pain [MD = −0.16, 95% CI (−0.79, 0.47), *I*^2^ = 72%, *p* = 0.62], WOMAC-stiffness [MD = −0.01, 95% CI (−0.31, 0.28), *I*^2^ = 0%, *p* = 0.94], and WOMAC-function [MD = −0.28, 95% CI (−0.57, 0.02), *I*^2^ = 0%, *p* = 0.07] (Fig 5). Meanwhile, four other studies reported WOMAC total scores, showing no significant difference between the two groups [MD = −0.12, 95% CI (−0.78, 0.54), *I*^2^ = 67%, *p* = 0.73]. Sensitivity analysis conducted by excluding one moderate-quality trial [44] while retaining three high-quality trials confirmed that there were no significant differences in WOMAC domain scores for pain, stiffness, and function between the two groups.

### Meta-analysis of TUG scores

**Fig 6:**
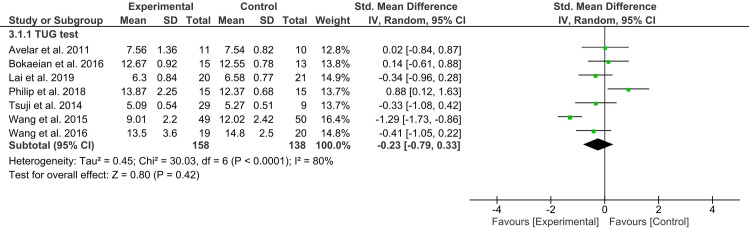
Forest plot of TUG scores for experimental versus control groups.

Seven studies (296 participants) reported TUG scores [21,32,34,35,37,40,44]. The meta-analysis found no significant difference in terms of improvement in functional mobility between the experimental and control groups [MD = −0.23, 95% CI (−0.79, 0.33), *I*^2^ = 80%, *p* = 0.42] (Fig 6). Similar results were observed in the sensitivity analysis after excluding three trials with moderate methodological quality [37,40,44].

### Meta-analysis of knee strength scores

**Fig 7:**
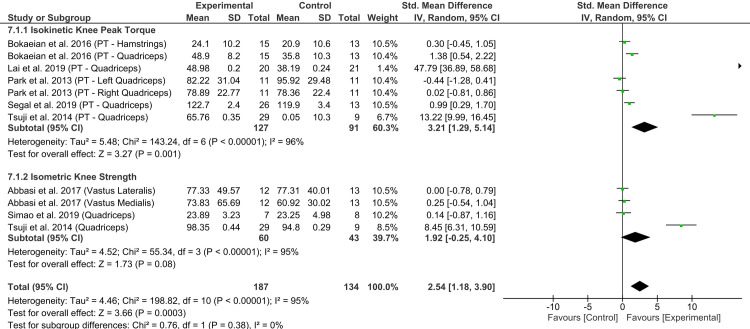
Forest plot of isometric knee strength and isokinetic knee peak torque scores for experimental versus control groups.

Cumulatively, seven studies reported outcomes on knee muscle strength. Based on the subgroup analyses for isokinetic knee peak torque and isometric knee strength, the experimental group showed a significant improvement in isokinetic knee peak torque compared to the control group [MD = 3.21, 95% CI (1.29, 5.14), *I*² = 0%, *p* = 0.001]; however, no significant findings were observed for isometric knee strength [MD = 1.92, 95% CI (−0.25, 4.10), *I*² = 0%, *p* = 0.08] (Fig 7). Sensitivity analysis focusing solely on high-quality trials [21,32,36,38,41] also showed similar results, indicating a significant improvement in isokinetic knee peak torque compared to the control group [MD = 3.17, 95% CI (0.56, 5.77), *I*² = 96%, *p* = 0.02] (S2 Fig); however, this analysis revealed a markedly high heterogeneity compared to the primary analysis.

### Meta-analysis of 6MWT scores

**Fig 8:**
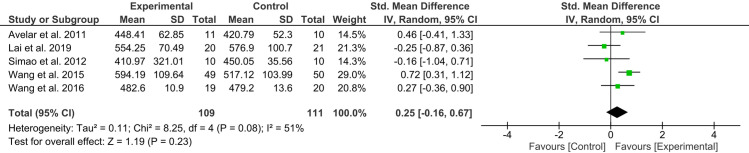
Forest plot of 6MWT scores for experimental versus control groups.

Five studies [32,34,35,42,44], comprising 220 participants, reported 6MWT scores. The meta-analysis revealed that the experimental group did not exhibit a significant improvement in functional mobility compared to the control group [MD = 0.25, 95% CI (−0.16, 0.67), I² = 51%, *p* = 0.23] (Fig 8). The results were consistent with the sensitivity analysis, which focused solely on high-quality trials [32,34,35,42].

### Meta-analysis of BBS scores

**Fig 9:**
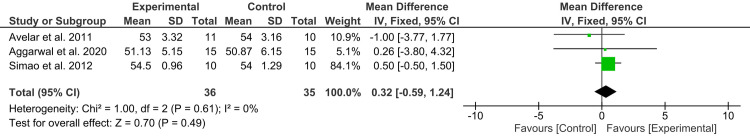
Forest plot of Berg Balance Scale scores for experimental versus control groups.

Three studies [20,42,44], involving a total of 71 participants, reported balance outcomes using the BBS. The meta-analysis revealed no significant difference in the BBS scores between the experimental and control groups [MD = 0.32, 95% CI (−0.59, 1.24), *I*² = 0%, *p* = 0.49] (Fig 9).

### Meta-analysis of CST scores

Two studies [11,21,32–35,37,41,42] involving a total of 51 participants (26 in the experimental group and 25 in the control group) reported scores on the 30-second CST. The analysis indicated no significant difference in CST scores between the experimental and control groups.

## Discussion

The literature describes WBVT as a neuromuscular and sensorimotor training method aimed at optimizing the integration of afferent information during specific exercise programs [45], emphasizing its potential to address key KOA rehabilitation goals. According to Wade et al. (2020), the primary goals of rehabilitation are to enhance muscle strength and physical activity, ultimately improving energy expenditure and cardiorespiratory function in daily life [46]. This systematic review analyzed 16 RCTs involving a total of 589 participants, investigating the effects of WBVT combined with conventional rehabilitation exercises on muscle strength, physical activity, and functional outcomes in patients with KOA. The findings underscore the potential of WBVT as a valuable adjunct to conventional rehabilitation methods, particularly in addressing common issues in KOA patients, such as pain and muscle weakness. These outcomes are critical in KOA rehabilitation, as muscle weakness and reduced activity levels are primary contributors to functional decline. While pain reduction is often a key outcome in KOA management, its significance lies in enabling increased activity and energy consumption, which are foundational to effective rehabilitation [47,48]. By incorporating WBVT into rehabilitation programs, significant improvements in pain, muscle strength, and overall physical performance can be achieved, offering a promising approach to enhancing the quality of life for individuals with KOA.

The findings found that WBVT combined with conventional rehabilitation exercises significantly reduced pain intensity and improved muscle strength in patients with KOA compared to those who only receive conventional rehabilitation exercises. Notably, the subgroup meta-analysis revealed that higher vibration frequencies were more effective in reducing pain. While improvements were observed in pain and muscle strength, measures of balance, physical function, functional mobility, and disability showed no significant differences between the groups. This conclusion is supported by high-certainty evidence, as 62% of the included trials were of high methodological quality. These findings suggest that WBVT could serve as a valuable adjunct to conventional rehabilitation exercises for managing pain, particularly at high vibration frequencies, and addressing muscle weakness in KOA patients. Pain reduction may indirectly contribute to strength improvements by alleviating arthrogenic muscle inhibition, a mechanism in which pain interferes with proper muscle activation [48]. Additionally, reducing pain may alleviate fear of movement and increase willingness to participate in physical activities, fostering improvements in physical fitness and functional mobility [47]. Whole-body vibration training, as a neuromuscular and sensorimotor training method, optimizes afferent information integration during exercise programs and can further support these outcomes [12]. By addressing sensory-motor interactions, WBVT enhances muscle strength, joint stability, and balance. This low-impact, high-efficiency exercise is particularly advantageous for older adults and individuals with joint limitations [49]. Strength training, including WBVT, has been shown to significantly improve static and dynamic balance in older adults [12], highlighting its potential as an effective intervention for individuals with KOA. Meanwhile, the efficacy of WBVT in reducing knee pain may be attributed to its ability to modulate the immune response and reduce the expression of inflammatory factors [18], thereby modulating pain perception, reducing pain and disability, and improving physical functioning [50]. It is also reported that WBVT may work by stimulating receptors in the skin and muscles, which in turn activate nerve stimulation that can lead to the release of endogenous analgesic substances (e.g., endorphins and serotonin), thereby reducing pain [51].

Our findings on the effects of WBVT on knee pain intensity are consistent with previous meta-analyses that have shown the beneficial effects of WBVT on KOA pain [9,24]. Previous meta-analyses concluded that WBVT can effectively reduce KOA pain [9,24]. In contrast, an earlier meta-analysis published in 2015 found no significant effect of WBVT on KOA pain, possibly due to the limited number of included studies (only five) [23]. Thus, it is believed that our findings are more reliable, as they were based on a larger sample analysis. Meanwhile, subgroup analysis indicating greater effectiveness of high-frequency vibration training underscores the importance of optimizing vibration parameters in clinical practice to achieve maximum pain relief in KOA patients. However, it is also worth noting that these comparisons were made based on heterogeneous study samples, as differences in factors such as adjuvant rehabilitation exercise selection, intervention duration, and vibration protocol might influence subgroup analysis outcomes.

Furthermore, regarding the clinical significance of our findings, it is essential to interpret the reported mean differences in light of established minimal clinically important differences (MCIDs) for KOA. For pain reduction, an MCID of ≥ 1.5 points on the VAS or NPRS has been suggested in the literature [52]. In this study, the mean difference in pain reduction for the experimental group was −0.43, which, while statistically significant, falls below the threshold of clinical significance as outlined by the MCID. Therefore, while WBVT shows promise in reducing knee pain intensity, further research is needed to establish optimal dosage parameters and clinical significance in line with established MCIDs.

The high heterogeneity observed across the studies, as indicated by the *I*² statistics, raises concerns regarding the generalizability of our findings. Variability in the sample populations, treatment protocols, and outcome measures can significantly affect the clinical applicability of our results [27]. To address these variations, apart from vibration frequency, research on other potential moderating factors such as patient age, severity of KOA, and duration of WBVT remains limited. To address this gap, future systematic reviews and meta-analyses should explore subgroup analyses based on these factors to provide more comprehensive insights into the differential effects of WBVT. While our study was unable to conduct such analyses due to limitations in sample size and available data, these variables are likely to have a significant influence on treatment outcomes. Future systematic reviews and meta-analyses should pre-specify subgroup analyses based on age, KOA severity, and WBVT duration in their protocols to ensure that such analyses are hypothesis-driven and not data-driven. Pre-specifying these factors would enhance the robustness of findings and avoid issues related to post hoc analyses. In our analysis, we chose to use a random-effects model to address this heterogeneity. We also looked into using a fixed-effects model for sensitivity analysis. Although we didn’t find any significant differences between the outcomes of the random and fixed models, we leaned toward the random-effects model because it’s a common approach in research that takes into account the variability across different studies [27]. This choice allows us to make broader inferences across various contexts and populations. Furthermore, employing a random-effects model supports the idea of adjusting for multiple comparisons [27], which can offer valuable insights into the reliability of the effects we’ve observed.

Furthermore, notable variability in the control group interventions across the included studies, particularly in the types and intensities of rehabilitation exercises, may have influenced these outcomes and contributed to the observed heterogeneity. Due to the high heterogeneity and the limited number of studies available, we were unable to conduct a sensitivity analysis based on the variability in control group interventions. We recognize this as a limitation, and future trials should aim to standardize rehabilitation interventions in control groups to minimize variability and enhance comparability of the results. This standardization will enable more definitive conclusions on the true effects of WBVT when combined with conventional rehabilitation. Apart from vibration frequency, research on other potential moderating factors such as patient age, severity of KOA, and duration of WBVT remains limited. To address this gap, future studies should explore subgroup analyses based on these factors to provide more comprehensive insights into the differential effects of WBVT. While our study was unable to conduct such analyses due to limitations in sample size and available data, these variables are likely to have a significant influence on treatment outcomes.

Our findings indicate that WBVT combined with exercise leads to a significant improvement in isokinetic knee muscle strength. These results align with previous studies demonstrating the efficacy of WBVT in enhancing muscle strength. For instance, Li et al. (2015) [23] observed similar improvements in strength metrics, although they did not find significant differences in pain and functional status. In contrast, Wang et al. (2015) [22] and Zafar et al. (2015) [24] reported improvements in physical function but not specifically in strength. The recent meta-analysis by Qiu et al. (2022) [9] highlighted the positive impact of WBVT on pain and physical function, yet our study uniquely integrates WBVT with conventional exercise therapy, providing a more comprehensive analysis of combined interventions. The improvement in knee muscle strength following WBVT intervention can be attributed to several mechanisms. Theoretically, WBVT enhances muscle strength by increasing muscle fiber activation and enhancing motor unit recruitment. The recruitment of more motor units and the activation of α-motor neurons through central nervous system stimulation play a critical role in enhancing knee strength [53]. Moreover, vibrations stimulate the expression of myogenic regulatory factors and promote cell proliferation, migration, and maturation, contributing to muscle hypertrophy and increased muscle cross-section [54]. Additionally, WBVT may improve bone density and morphology, thereby supporting muscle function and strength. This is believed to be due to vibration effects on increasing osteoblast activity and bone mineral content while improving bone morphology [55,56]. These mechanisms suggest that WBVT can complement conventional exercise therapy by targeting muscle weakness, a hallmark of KOA, thereby improving functional outcomes and overall rehabilitation efficacy.

Despite observing positive effects on pain relief and muscle strength, we found no statistically significant improvements in balance, functional mobility, or disability measures between the two groups studied. Previous reviews have reported mixed results: some showed significant improvements in these outcomes, while our findings, similar to those of Wang et al. (2015) [22] and Li et al. (2015) [23], align with studies like Zafar et al. (2015) [24], which reported no significant effects. This variability likely stems from methodological differences in WBVT protocols, including variations in vibration frequencies and durations. The observed discrepancies may also be attributed to differences in baseline characteristics among study populations, potentially diluting the therapeutic benefits. Moreover, heterogeneity in patient demographics and disease severity could have influenced these outcomes. It is essential to highlight that the limited number of studies [20,42,44] reporting these outcomes and the high level of heterogeneity may affect the conclusions drawn from this review. The small sample sizes in individual studies, particularly those focusing on balance [42,44] and functional mobility [37,44], restrict the power to detect significant differences. However, it is essential to note the limited number of studies reporting these outcomes; typically, only three studies comprising 71 participants [20,42,44] were analyzed for balance, and only two studies with 51 participants [20,44] were available for analysis of functional mobility and disability, reflecting small sample sizes that may limit conclusive effect size determination. Further research should focus on standardizing WBVT protocols and exploring patient-specific responses to clarify these discrepancies, particularly regarding the outcomes mentioned above. This underscores the urgent need for future studies employing more standardized protocols to better assess the efficacy of WBVT in improving these aspects of KOA management.

The present review acknowledges previous comparable reviews by Li et al. (2015) [23] and Qiu et al. (2022) [9]. Unlike Li et al. (2015), which only examined English-language publications, our study included both English and Chinese databases. Furthermore, we imposed more stringent methodological criteria by combining WBVT with exercise therapy in the experimental group, whereas Li et al. (2015) did not have this restriction. Li et al. (2015) reviewed five trials involving 168 participants and found; (i) no significant difference in muscle strength based on data from three studies (112 participants), and (ii) reported improved pain outcomes based on two studies (60 participants), but this finding was not statistically significant between groups [23]. The recent meta-analysis by Qiu et al. (2022) included 14 studies with 559 participants, eventually analyzing 10 studies in their meta-analysis. Qiu et al. (2022) included only English-language studies with control groups of exercise therapy, sham WBVT, and no exercise. In contrast, our study reviewed 16 trials and performed a meta-analysis of 15 trials involving 589 participants, demonstrating significant improvement in muscle strength (seven studies, 208 participants) and pain (nine studies, 374 participants), thereby updating and expanding the data.

This review has several strengths. First, it analyzes the combined effects of WBVT with conventional rehabilitation exercise, providing insights into their synergistic impact rather than focusing solely on WBVT alone. Second, the inclusion of recent literature ensures that findings are both up-to-date and relevant. Third, a comprehensive search strategy involving large databases enhances the study’s robustness, while the inclusion of publications in both English and Chinese contributes to a more diverse and inclusive dataset. Despite our rigorous search strategy and protocol, several limitations were identified. Firstly, the limited number of studies available for certain outcomes and high heterogeneity in comparators and vibration protocols, alongside small sample sizes in some articles [34,42], may affect the robustness of the findings. Secondly, while pain outcomes rely on subjective measures such as the VAS or NPRS, which are inherently subjective and may vary due to individual interpretations and reporting styles, the reliance on self-reported measures alone without corroborating objective measures could limit the accuracy of these findings. This variability can influence how pain scores are recorded and reported across studies [57]. Furthermore, the review exclusively examines WBVT in combination with conventional rehabilitation exercises, making it challenging to isolate the independent effects of WBVT. This combined approach reflects common clinical practices, as WBVT is rarely implemented as a standalone intervention. However, this means no conclusions can be drawn regarding the efficacy of WBVT alone, and future research should investigate its standalone effects to provide greater clarity on its specific contributions.

In future research, the integration of objective biomarkers for pain and muscle strength, such as inflammatory markers or muscle imaging, should be considered to complement subjective scales. Additionally, more high-quality trials are needed to clarify the effects of WBVT on other components of physical function and disability, especially given that 38% of the trials in this review were rated moderate to low quality based on PEDRO scores. Future studies should also expand their focus to broader functional outcomes like mobility, balance, and activities of daily living. These outcomes should be assessed using well-validated and standardized measures, such as the 6MWT, BBS, and TUG test, with longer follow-up periods to provide a more comprehensive understanding of WBVT’s potential in KOA rehabilitation and to evaluate the long-term sustainability of observed benefits. Furthermore, future trials should prioritize larger sample sizes and implement thorough blinding protocols to minimize bias and improve the generalizability of findings. In light of the findings presented in this review, it is important to acknowledge the limited exploration of the long-term sustainability of the observed benefits of WBVT in patients with KOA. Although our analysis demonstrates significant improvements in pain and muscle strength, the majority of the included studies primarily focused on short-term outcomes, leaving a gap in understanding the durability of these effects over extended periods. Assessing the long-term effects of WBVT, particularly over extended follow-up periods, will be critical to evaluate its sustained efficacy and clinical relevance in KOA populations.

## Conclusion

The findings of this review suggest that adding WBVT to conventional rehabilitation could be beneficial in the management of KOA, particularly for the clinical management of pain and muscle strength. However more high-quality investigations are needed to substantiate the effects on other outcomes such as in balance, functional mobility, and disability. It is recommended that future trials should focus on optimizing WBVT protocols to achieve MCIDs thresholds, particularly for pain and functional outcomes, to ensure meaningful clinical applications in KOA rehabilitation and examining long-term benefits to refine rehabilitation strategies for KOA patients.

## Supporting information

S1 Fig Sensitivity analysis of knee pain scores. (TIFF)

S2 Fig Sensitivity analysis of knee strength scores. (TIFF)

S1 Table Full-text screening table. (DOCX)

S2 Table PEDro scoring assessment details. (PDF)

S3 Table Cochrane risk-of-bias assessment details. (PDF)

S1 File PRISMA checklist. (PDF)

S2 File Data file (Excel format). (XLSX)
