# Supplementary figures and images for: Effects of whole-body vibration training as an adjunct to conventional rehabilitation exercise on pain, physical function and disability in knee osteoarthritis: A systematic review and meta-analysis
Source: PLoS One. 2025 Feb 10;20(2):e0318635. doi: 10.1371/journal.pone.0318635 (PMC11809854; doi:10.1371/journal.pone.0318635)

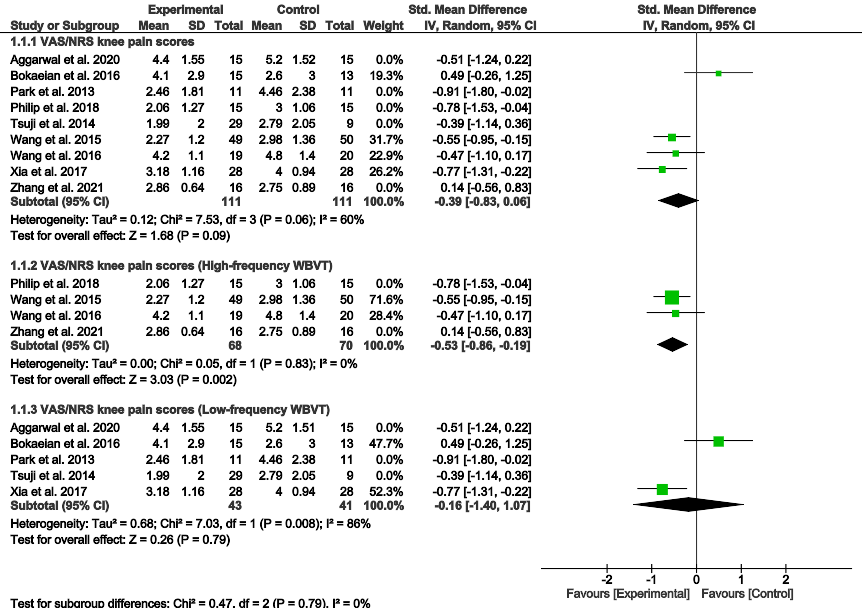

Supplement: S1 Fig — (TIFF) [file pone.0318635.s001.tiff]

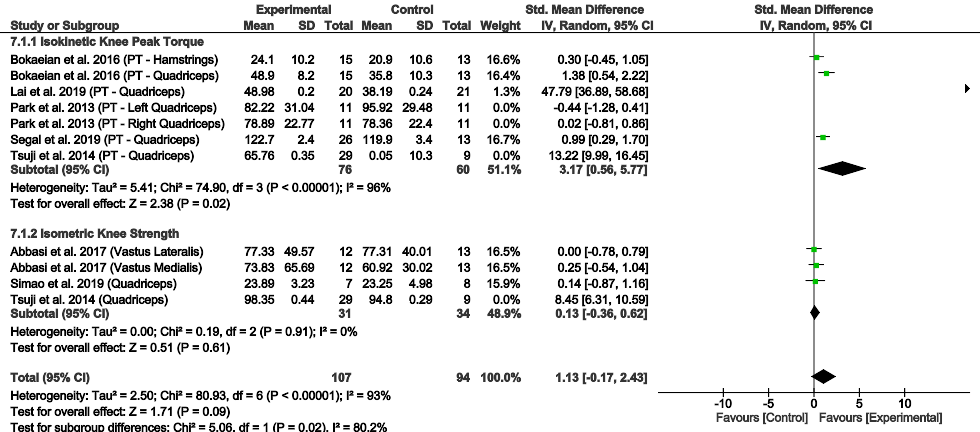

Supplement: S2 Fig — (TIFF) [file pone.0318635.s002.tiff]
