# Supplementary material for: Effects of whole-body vibration training as an adjunct to conventional rehabilitation exercise on pain, physical function and disability in knee osteoarthritis: A systematic review and meta-analysis
Source: PLoS One. 2025 Feb 10;20(2):e0318635. doi: 10.1371/journal.pone.0318635 (PMC11809854; doi:10.1371/journal.pone.0318635)
Supplement: S1 Table — (DOCX) [file pone.0318635.s003.docx]

**S1 Table. Screening Criteria for Inclusion and Exclusion of Articles (n = 446).**

Name of the extractors and assessors: Yan Peng, Mohd Azzuan Ahmad, Tay Yan Ling, Chai Siaw Chui

Date of full-text screening: 3rd – 9th March 2024

| No | Studies | Status (Included/ Excluded); If excluded, reason for exclusion |
| --- | --- | --- |
| 1 | Lu L, Mao L, Feng Y, Ainsworth BE, Liu Y, Chen N. Effects of different exercise training modes on muscle strength and physical performance in older people with sarcopenia: a systematic review and meta-analysis. BMC Geriatr. 2021 Dec 15;21(1):708. doi: 10.1186/s12877-021-02642-8. PMID: 34911483; PMCID: PMC8672633. | Excluded：review |
| 2 | Kochman M, Kasprzak M, Kielar A. ACL Reconstruction: Which Additional Physiotherapy Interventions Improve Early-Stage Rehabilitation? A Systematic Review. Int J Environ Res Public Health. 2022 Nov 29;19(23):15893. doi:10.3390/ijerph192315893. PMID: 36497965; PMCID: PMC9739138. | Excluded：review |
| 3 | Ferreira RM, Torres RT, Duarte JA, Gonçalves RS. Non-Pharmacological and Non-Surgical Interventions for Knee Osteoarthritis: A Systematic Review and Meta-Analysis. Acta Reumatol Port. 2019 Jul 29;44(3):173-217. English. PMID: 31356585. | Excluded：review |
| 4 | Letizia Mauro G, Scaturro D, Gimigliano F, Paoletta M, Liguori S, Toro G, Iolascon G, Moretti A. Physical Agent Modalities in Early Osteoarthritis: A Scoping Review. Medicina (Kaunas). 2021 Oct 26;57(11):1165. doi: 10.3390/medicina57111165. PMID: 34833383; PMCID: PMC8619194. | Excluded: review |
| 5 | Qiu CG, Chui CS, Chow SKH, Cheung WH, Wong RMY. Effects of Whole-Body Vibration Therapy on Knee Osteoarthritis: A Systematic Review and Meta-Analysis of Randomized Controlled Trials. J Rehabil Med. 2022 Mar 29;54:jrm00266. doi: 10.2340/jrm.v54.2032. PMID: 35174868; PMCID: PMC8963427. | Excluded：review |
| 6 | Arumugam A, Björklund M, Mikko S, Häger CK. Effects of neuromuscular training on knee proprioception in individuals with anterior cruciate ligament injury: a systematic review and GRADE evidence synthesis. BMJ Open. 2021 May 18;11(5):e049226. doi: 10.1136/bmjopen-2021-049226. PMID: 34006560; PMCID:  PMC8130739. | Excluded：review |
| 7 | Bennell KL, Hall M, Hinman RS. Osteoarthritis year in review 2015: rehabilitation and outcomes. Osteoarthritis Cartilage. 2016 Jan;24(1):58-70. doi: 10.1016/j.joca.2015.07.028. PMID: 26707993. | Excluded：review |
| 8 | Choursiya P, Tanwar T, Veqar Z. Effects of surface variability in whole-body vibration platform on knee osteoarthritis: A scoping review. J Bodyw Mov Ther.  2022 Oct;32:120-129. doi: 10.1016/j.jbmt.2022.04.007. Epub 2022 Apr 22. PMID:36180137. | Excluded：review |
| 9 | Abbott SC, Cole MD. Vibration arthrometry: a critical review. Crit Rev Biomed Eng. 2013;41(3):223-42. doi: 10.1615/critrevbiomedeng.2014010061. PMID:24579645. | Excluded：review |
| 10 | Alam MM, Khan AA, Farooq M. Effect of whole-body vibration on neuromuscular performance: A literature review. Work. 2018;59(4):571-583. doi:10.3233/WOR-182699. PMID: 29733043. | Excluded：review |
| 11 | Yu C, Zhao B, Li Y, Zang H, Li L. Vibrational Spectroscopy in Assessment of Early Osteoarthritis-A Narrative Review. Int J Mol Sci. 2021 May 15;22(10):5235. doi: 10.3390/ijms22105235. PMID: 34063436; PMCID: PMC8155859. | Excluded：review |
| 12 | Zafar H, Alghadir A, Anwer S, Al-Eisa E. Therapeutic effects of whole-body vibration training in knee osteoarthritis: a systematic review and meta-analysis. Arch Phys Med Rehabil. 2015 Aug;96(8):1525-32. doi:  10.1016/j.apmr.2015.03.010. Epub 2015 Mar 28. PMID: 25827655. | Excluded：review |
| 13 | Tan X, Jiang G, Zhang L, Wang D, Wu X. Effects of Whole-Body Vibration Training on Lower Limb Muscle Strength and Physical Performance Among Older Adults: A Systematic Review and Meta-analysis. Arch Phys Med Rehabil. 2023 Nov;104(11):1954-1965. doi: 10.1016/j.apmr.2023.04.002. Epub 2023 May 10. PMID:37169245. | Excluded：review |
| 14 | Ferreira RM, Duarte JA, Gonçalves RS. Non-pharmacological and non-surgical interventions to manage patients with knee osteoarthritis: An umbrella review. Acta Reumatol Port. 2018 Jul-Sep;43(3):182-200. English. PMID: 30414367. | Excluded：review |
| 15 | Moreira-Marconi E, Teixeira-Silva Y, Meirelles AG, Melo-Oliveira MES, Santos ACG, Reis-Silva A, Paineiras-Domingos LL, Seixas A, Dionello CDF, Sá-Caputo DDC, Bernardo-Filho M. Inflammatory Biomarker Responses to Whole-Body Vibration in Subjects with Different Clinical Status: A Systematic Review. Int J Environ Res Public Health. 2022 Nov 11;19(22):14853. doi: 10.3390/ijerph192214853. PMID:36429572; PMCID: PMC9690844. | Excluded：review |
| 16 | Oliveira S, Andrade R, Valente C, Espregueira-Mendes J, Silva F, Hinckel BB, Carvalho Ó, Leal A. Mechanical-based therapies may reduce pain and disability in some patients with knee osteoarthritis: A systematic review with meta-analysis. Knee. 2022 Aug;37:28-46. doi: 10.1016/j.knee.2022.05.005. Epub 2022 Jun 3. PMID:35660536. | Excluded：review |
| 17 | Spahn G, Lipfert JU, Maurer C, Hartmann B, Schiele R, Klemm HT, Grifka J, Hofmann GO. Risk factors for cartilage damage and osteoarthritis of the elbow joint: case-control study and systematic literature review. Arch Orthop Trauma Surg. 2017 Apr;137(4):557-566. doi: 10.1007/s00402-017-2654-6. Epub 2017 Feb 24.  PMID: 28236186. | Excluded：review |
| 18 | Sitjà-Rabert M, Rigau D, Fort Vanmeerghaeghe A, Romero-Rodríguez D, Bonastre Subirana M, Bonfill X. Efficacy of whole body vibration exercise in older people: a systematic review. Disabil Rehabil. 2012;34(11):883-93. doi: 10.3109/09638288.2011.626486. Epub 2012 Jan 6. PMID: 22225483. | Excluded：review |
| 19 | Anwer S, Alghadir A, Zafar H, Al-Eisa E. Effect of whole body vibration training on quadriceps muscle strength in individuals with knee osteoarthritis: a systematic review and meta-analysis. Physiotherapy. 2016 Jun;102(2):145-51. doi: 10.1016/j.physio.2015.10.004. Epub 2015 Oct 22. PMID: 26619822. | Excluded：review |
| 20 | Kang H, Lu J, Xu G. The effects of whole body vibration on muscle strength and functional mobility in persons with multiple sclerosis: A systematic review and meta-analysis. Mult Scler Relat Disord. 2016 May;7:1-7. doi: 10.1016/j.msard.2016.02.008. Epub 2016 Feb 8. PMID: 27237747. | Excluded：review |
| 21 | Wang P, Yang X, Yang Y, Yang L, Zhou Y, Liu C, Reinhardt JD, He C. Effects of whole body vibration on pain, stiffness and physical functions in patients with knee osteoarthritis: a systematic review and meta-analysis. Clin Rehabil. 2015 Oct;29(10):939-51. doi: 10.1177/0269215514564895. Epub 2014 Dec 18. PMID:25525066. | Excluded：review |
| 22 | Lu J, Xu G, Wang Y. Effects of whole body vibration training on people with chronic stroke: a systematic review and meta-analysis. Top Stroke Rehabil. 2015 Jun;22(3):161-8. doi: 10.1179/1074935714Z.0000000005. Epub 2015 Feb 24. PMID:26084320. | Excluded：review |
| 23 | Yang X, Wang P, Liu C, He C, Reinhardt JD. The effect of whole bodyvibration on balance, gait performance and mobility in people with stroke: a systematic review and meta-analysis. Clin Rehabil. 2015 Jul;29(7):627-38. doi:10.1177/0269215514552829. Epub 2014 Oct 13. PMID: 25311142. | Excluded：review |
| 24 | Lau RW, Liao LR, Yu F, Teo T, Chung RC, Pang MY. The effects of whole body vibration therapy on bone mineral density and leg muscle strength in older adults: a systematic review and meta-analysis. Clin Rehabil. 2011 Nov;25(11):975-88. doi: 10.1177/0269215511405078. Epub 2011 Aug 17. PMID:21849376. | Excluded：review |
| 25 | Oliveira LC, Oliveira RG, Pires-Oliveira DA. Effects of whole body vibration on bone mineral density in postmenopausal women: a systematic review and meta-analysis. Osteoporos Int. 2016 Oct;27(10):2913-33. doi:  10.1007/s00198-016-3618-3. Epub 2016 May 4. PMID: 27145947. | Excluded：review |
| 26 | Gignac MAM, Irvin E, Cullen K, Van Eerd D, Beaton DE, Mahood Q, McLeod C, Backman CL. Men and Women's Occupational Activities and the Risk of Developing Osteoarthritis of the Knee, Hip, or Hands: A Systematic Review and Recommendations for Future Research. Arthritis Care Res (Hoboken). 2020 Mar;72(3):378-396. doi: 10.1002/acr.23855. PMID: 30762317; PMCID: PMC7065017. | Excluded：review |
| 27 | Ji Q, He H, Zhang C, Lu C, Zheng Y, Luo XT, He C. Effects of whole-body vibration on neuromuscular performance in individuals with spinal cord injury: a  systematic review. Clin Rehabil. 2017 Oct;31(10):1279-1291. doi: 10.1177/0269215516671014. Epub 2016 Sep 29. PMID: 27688299. | Excluded：review |
| 28 | Madeleine P, Andersen RE, Larsen JB, Arendt-Nielsen L, Samani A. Wireless multichannel vibroarthrographic recordings for the assessment of knee osteoarthritis during three activities of daily living. Clin Biomech (Bristol, Avon). 2020 Feb;72:16-23. doi: 10.1016/j.clinbiomech.2019.11.015. Epub 2019 Nov  26. PMID: 31794924. | Excluded：review |
| 29 | Seixas A, Sañudo B, Sá-Caputo D, Taiar R, Bernardo-Filho M. Whole-Body Vibration for Individuals with Reconstructed Anterior Cruciate Ligament: A Systematic Review. Biomed Res Int. 2020 May 1;2020:7362069. doi: 10.1155/2020/7362069. PMID: 32462013; PMCID: PMC7212274. | Excluded：review |
| 30 | Osawa Y, Oguma Y, Ishii N. The effects of whole-body vibration on muscle strength and power: a meta-analysis. J Musculoskelet Neuronal Interact. 2013 Sep;13(3):380-90. PMID: 23989260. | Excluded：review |
| 31 | Dong Y, Wang W, Zheng J, Chen S, Qiao J, Wang X. Whole Body Vibration Exercise for Chronic Musculoskeletal Pain: A Systematic Review and Meta-analysis of Randomized Controlled Trials. Arch Phys Med Rehabil. 2019 Nov;100(11):2167-2178. doi: 10.1016/j.apmr.2019.03.011. Epub 2019 Apr 17. PMID:  31004565. | Excluded：review |
| 32 | Bączkowicz D, Kręcisz K. Vibroarthrography in the evaluation of musculoskeletal system - a pilot study. Ortop Traumatol Rehabil. 2013 Oct 31;15(5):407-16. doi: 10.5604/15093492.1084242. PMID: 24431252. | Excluded：review |
| 33 | Allen R, De Stefano A. A data acquisition and analysis system for the biomechanical evaluation of patient moving and transferring equipment and procedures. J Med Eng Technol. 2007 Jan-Feb;31(1):14-23. doi:10.1080/03091900500233593. PMID: 17365422. | Excluded：review |
| 34 | Leite HR, Camargos ACR, Mendonça VA, Lacerda ACR, Soares BA, Oliveira VC. Current evidence does not support whole body vibration in clinical practice in  children and adolescents with disabilities: a systematic review of randomized controlled trial. Braz J Phys Ther. 2019 May-Jun;23(3):196-211. doi: 10.1016/j.bjpt.2018.09.005. Epub 2018 Sep 19. PMID: 30245042; PMCID: PMC6531669. | Excluded：review |
| 35 | Melnyk M, Kofler B, Faist M, Hodapp M, Gollhofer A. Effect of a whole-body vibration session on knee stability. Int J Sports Med. 2008 Oct;29(10):839-44. doi: 10.1055/s-2008-1038405. Epub 2008 Apr 9. PMID: 18401809. | Excluded：review |
| 36 | Shinohara M, Sabra K, Gennisson JL, Fink M, Tanter M. Real-time visualization of muscle stiffness distribution with ultrasound shear wave imaging during muscle contraction. Muscle Nerve. 2010 Sep;42(3):438-41. doi: 10.1002/mus.21723. PMID: 20665510. | Excluded：review |
| 37 | Tihanyi TK, Horváth M, Fazekas G, Hortobágyi T, Tihanyi J. One session of whole body vibration increases voluntary muscle strength transiently in patients with stroke. Clin Rehabil. 2007 Sep;21(9):782-93. doi: 10.1177/0269215507077814. PMID: 17875558. | Excluded：review |
| 38 | Mulder ER, Stegeman DF, Gerrits KH, Paalman MI, Rittweger J, Felsenberg D, de Haan A. Strength, size and activation of knee extensors followed during 8 weeks of horizontal bed rest and the influence of a countermeasure. Eur J Appl Physiol. 2006 Aug;97(6):706-15. doi: 10.1007/s00421-006-0241-6. Epub 2006 Jun 20. PMID: 16786354. | Excluded：review |
| 39 | Cook DP, Mileva KN, James DC, Zaidell LN, Goss VG, Bowtell JL. Triaxial modulation of the acceleration induced in the lower extremity during whole-body vibration training: a pilot study. J Strength Cond Res. 2011 Feb;25(2):298-308. doi: 10.1519/JSC.0b013e3181be3003. PMID: 20040893. | Excluded：review |
| 40 | Moreira-Marconi E, Moura-Fernandes MC, Lopes-Souza P, Teixeira-Silva Y, Reis-Silva A, Marchon RM, Guedes-Aguiar EO, Paineiras-Domingos LL, Sá-Caputo DDC, Morel DS, Dionello CF, De-Carvalho SO, Pereira MJDS, Francisca-Santos A, Silva-Costa G, Olímpio-Souza M, Lemos-Santos TR, Asad NR, Xavier VL, Taiar R, Sonza A, Seixas A, Cochrane DJ, Bernardo-Filho M. Evaluation of the temperature of posterior lower limbs skin during the whole body vibration measured by infrared thermography: Cross-sectional study analysis using linear mixed effect model. PLoS One. 2019 Mar 13;14(3):e0212512. doi: 10.1371/journal.pone.0212512. PMID: 30865641; PMCID: PMC6415782. | Excluded：review |
| 41 | Ahlborg L, Andersson C, Julin P. Whole-body vibration training compared with resistance training: effect on spasticity, muscle strength and motor performance in adults with cerebral palsy. J Rehabil Med. 2006 Sep;38(5):302-8. doi: 10.1080/16501970600680262. PMID: 16931460. | Excluded：review |
| 42 | Mazzetto MO, Hotta TH, Carrasco TG, Mazzetto RG. Characteristics of TMD noise analyzed by electrovibratography. Cranio. 2008 Jul;26(3):222-8. doi: 10.1179/crn.2008.030. PMID: 18686500. | Excluded：review |
| 43 | Togo S, Kagawa T, Uno Y. Change of a motor synergy for dampening handvibration depending on a task difficulty. Exp Brain Res. 2014 Oct;232(10):3101-9. doi: 10.1007/s00221-014-3994-x. Epub 2014 Jun 4. PMID:24894587. | Excluded：review |
| 44 | Manó S, Pálinkás J, Szabó J, Nagy JT, Bakó K, Csernátony Z. Application of a vibrating device for the prevention of flexion contracture after total knee arthroplasty. Eur J Orthop Surg Traumatol. 2015 Jan;25(1):167-72. doi: 10.1007/s00590-014-1466-4. Epub 2014 Apr 29. PMID: 24777702. | Excluded：review |
| 45 | Lawrence JM, Stroman PW, Kollias SS. Functional magnetic resonance imaging of the human spinal cord during vibration stimulation of different dermatomes. Neuroradiology. 2008 Mar;50(3):273-80. doi: 10.1007/s00234-007-0338-6. Epub 2007 Nov 20. PMID: 18026942. | Excluded：review |
| 46 | Delecluse C, Roelants M, Diels R, Koninckx E, Verschueren S. Effects of whole body vibration training on muscle strength and sprint performance in sprint-trained athletes. Int J Sports Med. 2005 Oct;26(8):662-8. doi: 10.1055/s-2004-830381. PMID: 16158372. | Excluded：review |
| 47 | Millichap JJ, Sy BT, Leacock RO. Spinal cord infarction with multiple etiologic factors. J Gen Intern Med. 2007 Jan;22(1):151-4. doi:10.1007/s11606-006-0029-8. PMID: 17351858; PMCID: PMC1824728. | Excluded：review |
| 48 | Fattorini L, Ferraresi A, Rodio A, Azzena GB, Filippi GM. Motor performance changes induced by muscle vibration. Eur J Appl Physiol. 2006 Sep;98(1):79-87.  doi: 10.1007/s00421-006-0250-5. Epub 2006 Aug 9. PMID: 16896736. | Excluded：review |
| 49 | Yamada E, Kusaka T, Miyamoto K, Tanaka S, Morita S, Tanaka S, Tsuji S, Mori S, Norimatsu H, Itoh S. Vastus lateralis oxygenation and blood volume measured by near-infrared spectroscopy during whole body vibration. Clin Physiol Funct Imaging. 2005 Jul;25(4):203-8. doi: 10.1111/j.1475-097X.2005.00614.x. PMID: 15972021. | Excluded：review |
| 50 | Umapathy K, Krishnan S. Modified local discriminant bases algorithm and its application in analysis of human knee joint vibration signals. IEEE Trans Biomed Eng. 2006 Mar;53(3):517-23. doi: 10.1109/TBME.2005.869787. PMID: 16532778. | Excluded：review |
| 51 | Dahl MC, Kramer PA, Reinhall PG, Benirschke SK, Hansen ST, Ching RP. The efficacy of using vibrometry to detect osteointegration of the Agility total ankle. J Biomech. 2010 Jun 18;43(9):1840-3. doi: 10.1016/j.jbiomech.2010.02.019. Epub 2010 Mar 1. PMID: 20189573. | Excluded：review |
| 52 | Duc S, Munera M, Chiementin X, Bertucci W. Effect of vibration frequency and angle knee flexion on muscular activity and transmissibility function during static whole body vibration exercise. Comput Methods Biomech Biomed Engin. 2014;17 Suppl 1:116-7. doi: 10.1080/10255842.2014.931511. PMID: 25074192. | Excluded：review |
| 53 | Pel JJ, Bagheri J, van Dam LM, van den Berg-Emons HJ, Horemans HL, Stam HJ, van der Steen J. Platform accelerations of three different whole-body vibration devices and the transmission of vertical vibrations to the lower limbs. Med Eng Phys. 2009 Oct;31(8):937-44. doi: 10.1016/j.medengphy.2009.05.005. Epub 2009 Jun  11. PMID: 19523867. | Excluded：review |
| 54 | Lopate G, Pestronk A, Al-Lozi M, Lynch T, Florence J, Miller T, Levine T, Rampy T, Beson B, Ramneantu I. Peripheral neuropathy in an outpatient cohort of patients with Sjögren's syndrome. Muscle Nerve. 2006 May;33(5):672-6. doi: 10.1002/mus.20514. PMID: 16453296. | Excluded：review |
| 55 | Botez SA, Liu G, Logigian E, Herrmann DN. Is the bedside timed vibration test reliable? Muscle Nerve. 2009 Feb;39(2):221-3. doi: 10.1002/mus.21143. PMID:  19145659. | Excluded：review |
| 56 | Konishi Y, Kasukawa T, Tobita H, Nishino A, Konishi M. Gamma loop dysfunction of the quadriceps femoris of elderly patients hospitalized after fall injury. J Geriatr Phys Ther. 2007;30(2):54-9. doi: 10.1519/00139143-200708000-00004. PMID: 18171488. | Excluded：review |
| 57 | Garatachea N, Jiménez A, Bresciani G, Mariño NA, González-Gallego J, de Paz JA. The effects of movement velocity during squatting on energy expenditure and substrate utilization in whole-body vibration. J Strength Cond Res. 2007 May;21(2):594-8. doi: 10.1519/R-20566.1. PMID: 17530981. | Excluded：review |
| 58 | Häggström E, Hagberg K, Rydevik B, Brånemark R. Vibrotactile evaluation: osseointegrated versus socket-suspended transfemoral prostheses. J Rehabil Res  Dev. 2013;50(10):1423-34. doi: 10.1682/JRRD.2012.08.0135. PMID: 24699977. | Excluded：review |
| 59 | Nalband S, Sundar A, Prince AA, Agarwal A. Feature selection and classification methodology for the detection of knee-joint disorders. Comput Methods Programs Biomed. 2016 Apr;127:94-104. doi: 10.1016/j.cmpb.2016.01.020. Epub 2016 Jan 29. PMID: 27000292. | Excluded：review |
| 60 | Rangayyan RM, Wu Y. Analysis of vibroarthrographic signals with features related to signal variability and radial-basis functions. Ann Biomed Eng. 2009 Jan;37(1):156-63. doi: 10.1007/s10439-008-9601-1. Epub 2008 Nov 18. PMID:19015987. | Excluded：review |
| 61 | Yang S, Cai S, Zheng F, Wu Y, Liu K, Wu M, Zou Q, Chen J. Representation of fluctuation features in pathological knee joint vibroarthrographic signals using kernel density modeling method. Med Eng Phys. 2014 Oct;36(10):130511.doi:10.1016/j.medengphy.2014.07.008. Epub 2014 Aug 3. PMID: 25096412. | Excluded：review |
| 62 | Konishi YU. ACL repair might induce further abnormality of gamma loop in the intact side of the quadriceps femoris. Int J Sports Med. 2011 Apr;32(4):292-6. doi: 10.1055/s-0030-1270488. Epub 2011 Mar 4. PMID: 21380979. | Excluded：review |
| 63 | Spain RI, Leist TP, De Sousa EA. When metals compete: a case of copper- deficiency myeloneuropathy and anemia. Nat Clin Pract Neurol. 2009 Feb;5(2):106-11. doi: 10.1038/ncpneuro1008. PMID: 19194390. | Excluded：review |
| 64 | Bi X, Li G, Doty SB, Camacho NP. A novel method for determination of collagen orientation in cartilage by Fourier transform infrared imaging spectroscopy (FT-IRIS). Osteoarthritis Cartilage. 2005 Dec;13(12):1050-8. doi:  10.1016/j.joca.2005.07.008. Epub 2005 Sep 9. PMID: 16154778. | Excluded：review |
| 65 | Cochrane DJ, Loram ID, Stannard SR, Rittweger J. Changes in joint angle, muscle-tendon complex length, muscle contractile tissue displacement, and modulation of EMG activity during acute whole-body vibration. Muscle Nerve. 2009 Sep;40(3):420-9. doi: 10.1002/mus.21330. PMID: 19618430. | Excluded：review |
| 66 | Colson SS, Petit PD, Hébreard L, Tessaro J, Pensini M. Whole body vibration does not enhance muscle activation. Int J Sports Med. 2009 Dec;30(12):841-4. doi: 10.1055/s-0029-1234082. PMID: 19885777. | Excluded：review |
| 67 | Pamukoff DN, Ryan ED, Blackburn JT. The acute effects of local muscle vibration frequency on peak torque, rate of torque development, and EMG activity. J Electromyogr Kinesiol. 2014 Dec;24(6):888-94. doi: 10.1016/j.jelekin.2014.07.014. Epub 2014 Aug 7. PMID: 25169762. | Excluded：review |
| 68 | Rangayyan RM, Wu YF. Screening of knee-joint vibroarthrographic signals using statistical parameters and radial basis functions. Med Biol Eng Comput. 2008 Mar;46(3):223-32. doi: 10.1007/s11517-007-0278-7. Epub 2007 Oct 25. PMID:17960443. | Excluded：review |
| 69 | Finke M, Schweikard A. Motorization of a surgical microscope for intra- operative navigation and intuitive control. Int J Med Robot. 2010 Sep;6(3):269-80. doi: 10.1002/rcs.314. PMID: 20812267. | Excluded：review |
| 70 | Honda K, Natsumi Y, Urade M. Correlation between MRI evidence of degenerative condylar surface changes, induction of articular disc displacement and pathological joint sounds in the temporomandibular joint. Gerodontology. 2008 Dec;25(4):251-7. doi: 10.1111/j.1741-2358.2008.00219.x. Epub 2008 Feb 27. PMID: 18312371. | Excluded：review |
| 71 | Tenzer Y, Davies B, Rodriguez y Baena F. Investigation into the effectiveness of vibrotactile feedback to improve the haptic realism of an arthroscopy training simulator. Stud Health Technol Inform. 2008;132:517-22. PMID: 18391360. | Excluded：review |
| 72 | Munera M, Chiementin X, Duc S, Bertucci W. Transmission of whole-body vibration to lower limb during dynamic squat exercise. Comput Methods Biomech Biomed Engin. 2014;17 Suppl 1:148-9. doi: 10.1080/10255842.2014.931565. PMID: 25074208. | Excluded：review |
| 73 | Kammers MP, van der Ham IJ, Dijkerman HC. Dissociating body representations in healthy individuals: differential effects of a kinaesthetic illusion on perception and action. Neuropsychologia. 2006;44(12):2430-6. doi: 10.1016/j.neuropsychologia.2006.04.009. Epub 2006 Jun 5. PMID: 16750227. | Excluded：review |
| 74 | Celletti C, Castori M, Galli M, Rigoldi C, Grammatico P, Albertini G, Camerota F. Evaluation of balance and improvement of proprioception by repetitive muscle vibration in a 15-year-old girl with joint hypermobility syndrome. Arthritis Care Res (Hoboken). 2011 May;63(5):775-9. doi: 10.1002/acr.20434. PMID: 21240965. | Excluded：review |
| 75 | Qu X. Low-level noise affects balance control differently when applied at different body parts. J Biomech. 2010 Nov 16;43(15):2936-40. doi: 10.1016/j.jbiomech.2010.07.010. Epub 2010 Aug 11. PMID: 20705293. | Excluded：review |
| 76 | Mutch JA, Johansson JE. Occlusion of the artery of Adamkiewicz after hip and knee arthroplasty. J Arthroplasty. 2011 Apr;26(3):505.e5-8. doi: 10.1016/j.arth.2010.03.030. Epub 2010 May 31. PMID: 20570093. | Excluded：review |
| 77 | Heathers JA. Vibration training and body fat: a comment on Artero et al. (2011). Eur J Appl Physiol. 2012 Jun;112(6):2381-2. doi: 10.1007/s00421-011-2179-6. Epub 2011 Sep 29. PMID: 21956487. | Excluded：review |
| 78 | Mu T, Nandi AK, Rangayyan RM. Screening of knee-joint vibroarthrographic signals using the strict 2-surface proximal classifier and genetic algorithm. Comput Biol Med. 2008 Oct;38(10):1103-11. doi: 10.1016/j.compbiomed.2008.08.009. Epub 2008 Sep 27. PMID: 18823882. | Excluded：review |
| 79 | Zander T, Rabellino M, Baldi S, Blasco O, Maynar M. Infrainguinal revascularization using the Crosser vibrational system. Minim Invasive Ther Allied Technol. 2010 Aug;19(4):231-6. doi: 10.3109/13645701003644137. PMID: 20158407. | Excluded：review |
| 80 | Xue D, Zheng Q, Pan Z. Vibration may be a potential non-pharmacological treatment of osteoarthritis. Med Hypotheses. 2009 May;72(5):612. doi:10.1016/j.mehy.2008.12.015. Epub 2009 Jan 24. PMID: 19168290. | Excluded：review |
| 81 | Rubak TS, Svendsen SW, Andersen JH, Haahr JP, Kryger A, Jensen LD, Frost P. An expert-based job exposure matrix for large scale epidemiologic studies of primary hip and knee osteoarthritis: the Lower Body JEM. BMC Musculoskelet Disord. 2014 Jun 13;15:204. doi: 10.1186/1471-2474-15-204. PMID: 24927760; PMCID: PMC4067499. | Excluded：review |
| 82 | Stojanović SP, Zivić L, Stojanović J, Belić B. Total fixation of cricoarytenoid joint of a patient with rheumatoid arthritis and Hashimoto thyroiditis. Srp Arh Celok Lek. 2010 Mar-Apr;138(3-4):230-2. doi: 10.2298/sarh1004230s. PMID: 20499506. | Excluded：review |
| 83 | Lamont HS, Cramer JT, Bemben DA, Shehab RL, Anderson MA, Bemben MG. Effects of adding whole body vibration to squat training on isometric force/time characteristics. J Strength Cond Res. 2010 Jan;24(1):171-83. doi: 10.1519/JSC.0b013e3181c3b641. PMID: 19924007. | Excluded：review |
| 84 | Cairns NJ, Adam CJ, Pearcy MJ, Smeathers J. Evaluation of modal analysis techniques using physical models to detect osseointegration of implants in transfemoral amputees. Annu Int Conf IEEE Eng Med Biol Soc. 2011;2011:1600-3. doi: 10.1109/IEMBS.2011.6090463. PMID: 22254628. | Excluded：review |
| 85 | Currie KB, Tadisina KK, Mackinnon SE. Common Hand Conditions: A Review. JAMA. 2022 Jun 28;327(24):2434-2445. doi: 10.1001/jama.2022.8481. Erratum in: JAMA. 2023 Aug 22;330(8):772. doi: 10.1001/jama.2022.14783. PMID: 35762992. | Excluded：review |
| 86 | Fransson PA, Hjerpe M, Johansson R. Adaptation of multi-segmented body movements during vibratory proprioceptive and galvanic vestibular stimulation. J Vestib Res. 2007;17(1):47-62. PMID: 18219104. | Excluded：review |
| 87 | Hafstrom A, Patel M, Modig F, Magnusson M, Fransson PA. Acute alcohol intoxication impairs segmental body alignment in upright standing. J Vestib Res. 2014;24(4):297-304. doi: 10.3233/VES-140513. PMID: 25095774. | Excluded：review |
| 88 | Mildren RL, Yip MC, Lowrey CR, Harpur C, Brown SHM, Bent LR. Ageing reduces light touch and vibrotactile sensitivity on the anterior lower leg and foot dorsum. Exp Gerontol. 2017 Dec 1;99:1-6. doi: 10.1016/j.exger.2017.09.007. Epub2017 Sep 15. PMID: 28919542. | Excluded：review |
| 89 | Santos MJ, Pedro LM, Canhão H, Fernandes E Fernandes J, Canas da Silva J, Fonseca JE, Saldanha C. Hemorheological parameters are related to subclinical atherosclerosis in systemic lupus erythematosus and rheumatoid arthritis patients. Atherosclerosis. 2011 Dec;219(2):821-6. doi: 10.1016/j.atherosclerosis.2011.08.026. Epub 2011 Aug 22. PMID: 21906736. | Excluded：review |
| 90 | Jacobs CR, Temiyasathit S, Castillo AB. Osteocyte mechanobiology and pericellular mechanics. Annu Rev Biomed Eng. 2010 Aug 15;12:369-400. doi: 10.1146/annurev-bioeng-070909-105302. PMID: 20617941. | Excluded：review |
| 91 | Shehata AW, Keri MI, Gomez M, Marasco PD, Vette AH, Hebert JS. Skin Stretch Enhances Illusory Movement in Persons with Lower-Limb Amputation. IEEE Int Conf Rehabil Robot. 2019 Jun;2019:1233-1238. doi: 10.1109/ICORR.2019.8779477. PMID: 31374798. | Excluded：review |
| 92 | Agarwal V, Singh R, Wiclaf, Chauhan S, Tahlan A, Ahuja CK, Goel D, Pal L. A clinical, electrophysiological, and pathological study of neuropathy in rheumatoid arthritis. Clin Rheumatol. 2008 Jul;27(7):841-4. doi: 10.1007/s10067-007-0804-x. Epub 2007 Dec 15. PMID: 18084807. | Excluded：review |
| 93 | Raynor MB, Kuhn JE. Utility of features of the patient's history in the diagnosis of atraumatic shoulder pain: a systematic review. J Shoulder Elbow Surg. 2016 Apr;25(4):688-94. doi: 10.1016/j.jse.2015.09.023. Epub 2015 Dec 23. PMID: 26711472. | Excluded：review |
| 94 | Cheng HY, Yu YC, Wong AM, Tsai YS, Ju YY. Effects of an eight-week whole body vibration on lower extremity muscle tone and function in children with cerebral palsy. Res Dev Disabil. 2015 Mar;38:256-61. doi: 10.1016/j.ridd.2014.12.017. Epub 2015 Jan 7. PMID: 25575288. | Excluded：review |
| 95 | Stanciu S, Cîrmaci M, Berghea F, Bugaru M, Ciobica L, Jurcuţ C, Cherecheş T, Blaj S. Vibroarthrography--a possible functional non-invasive method for early detection damaged cartilage joint. Rom J Intern Med. 2006;44(4):471-6. PMID: 18386624. | Excluded：review |
| 96 | Liu Y, Fan Y, Chen X. Effects of whole-body vibration training in static and dynamic semi-squat patterns on the lower limb muscle activity. Sci Rep. 2023 Sep 2;13(1):14432. doi: 10.1038/s41598-023-40985-x. PMID: 37660154; PMCID: PMC10475118. | Excluded：review |
| 97 | Park JM, Park S, Jee YS. Rehabilitation Program Combined with Local Vibroacoustics Improves Psychophysiological Conditions in Patients with ACL Reconstruction. Medicina (Kaunas). 2019 Sep 30;55(10):659. doi: 10.3390/medicina55100659. PMID: 31574964; PMCID: PMC6843143. | Excluded：review |
| 98 | Xue L, Sun P. Detection of minor salivary glands affected in Sjogren's syndrome by Raman spectroscopy. Med Hypotheses. 2011 Feb;76(2):176-7. doi: 10.1016/j.mehy.2010.09.010. Epub 2010 Oct 8. PMID: 20934259. | Excluded：review |
| 99 | Sokmen O, Gocmen R, Nurlu G, Karabudak R. Combined Central and Peripheral Demyelination in a Case With Sjogren Syndrome. Neurologist. 2022 Nov 1;27(6):354-356. doi: 10.1097/NRL.0000000000000411. PMID: 35051965. | Excluded：review |
| 100 | Carvalho RL, Almeida GL. The effect of vibration on postural response of Down syndrome individuals on the seesaw. Res Dev Disabil. 2009 Nov-Dec;30(6):1124-31. doi: 10.1016/j.ridd.2009.02.012. Epub 2009 Apr 25. PMID:  19394195. | Excluded：review |
| 101 | Bergmann G, Bender A, Dymke J, Duda GN, Damm P. Physical Activities That Cause High Friction Moments at the Cup in Hip Implants. J Bone Joint Surg Am.2018 Oct 3;100(19):1637-1644. doi: 10.2106/JBJS.17.01298. PMID: 30277993. | Excluded：review |
| 102 | Kruse LM, Gray B, Wright RW. Rehabilitation after anterior cruciate ligament reconstruction: a systematic review. J Bone Joint Surg Am. 2012 Oct 3;94(19):1737-48. doi: 10.2106/JBJS.K.01246. PMID: 23032584; PMCID: PMC3448301. | Excluded：review |
| 103 | Keri MI, Shehata AW, Marasco PD, Hebert JS, Vette AH. A Cost-Effective Inertial Measurement System for Tracking Movement and Triggering Kinesthetic Feedback in Lower-Limb Prosthesis Users. Sensors (Basel). 2021 Mar 6;21(5):1844. doi: 10.3390/s21051844. PMID: 33800790; PMCID: PMC7961441. | Excluded：review |
| 104 | Russell K, Nicholson R, Naidu R. Reducing the pain of intramuscular benzathine penicillin injections in the rheumatic fever population of Counties Manukau District Health Board. J Paediatr Child Health. 2014 Feb;50(2):112-7. doi: 10.1111/jpc.12400. Epub 2013 Oct 18. PMID: 24134180. | Excluded：review |
| 105 | Bertucco M, Nardon M, Mueske N, Sandhu S, Rethlefsen SA, Wren TAL, Sanger TD. The Effects of Prolonged Vibrotactile EMG-Based Biofeedback on Ankle Joint Range of Motion During Gait in Children with Spastic Cerebral Palsy: A Case Series. Phys Occup Ther Pediatr. 2023;43(3):351-366. doi: 10.1080/01942638.2022.2151391. Epub 2022 Nov 29. PMID: 36446743. | Excluded：review |
| 106 | Barton A, McLean B. An unusual case of peripheral neuropathy possibly due to arsenic toxicity secondary to excessive intake of dietary supplements. Ann Clin Biochem. 2013 Sep;50(Pt 5):496-500. doi: 10.1177/0004563212473276. Epub 2013 May 29. PMID: 23761379. | Excluded：review |
| 107 | Rangayyan RM, Wu Y. Modeling and classification of knee-joint vibroarthrographic signals using probability density functions estimated with Parzen windows. Annu Int Conf IEEE Eng Med Biol Soc. 2008;2008:2099-102. doi:  10.1109/IEMBS.2008.4649607. PMID: 19163110. | Excluded：review |
| 108 | Flanagan EP, Leep Hunderfund AN, Kumar N, Murray JA, Krecke KN, Katz BS, Pittock SJ. Clinical reasoning: a 55-year-old man with weight loss, ataxia, and foot drop. Neurology. 2014 Jun 17;82(24):e214-9. doi: 10.1212/WNL.0000000000000525. PMID: 24960835; PMCID: PMC4113461. | Excluded：review |
| 109 | Booth S, Chohan S, Curran JC, Karrison T, Schmitz A, Utset TO. Whole blood viscosity and arterial thrombotic events in patients with systemic lupus erythematosus. Arthritis Rheum. 2007 Jun 15;57(5):845-50. doi: 10.1002/art.22766. PMID: 17530685. | Excluded：review |
| 110 | atel M, Fransson PA, Karlberg M, Malmstrom EM, Magnusson M. Change of body movement coordination during cervical proprioceptive disturbances with increased  age. Gerontology. 2010;56(3):284-90. doi: 10.1159/000265750. Epub 2009 Dec 10. PMID: 20016118. | Excluded：review |
| 111 | Wu Y, Chen P, Luo X, Huang H, Liao L, Yao Y, Wu M, Rangayyan RM. Quantification of knee vibroarthrographic signal irregularity associated with patellofemoral joint cartilage pathology based on entropy and envelope amplitude measures. Comput Methods Programs Biomed. 2016 Jul;130:1-12. doi: 10.1016/j.cmpb.2016.03.021. Epub 2016 Mar 21. PMID: 27208516. | Excluded：review |
| 112 | Charlton JM, Xia H, Shull PB, Hunt MA. Validity and reliability of a shoe-embedded sensor module for measuring foot progression angle during over-ground walking. J Biomech. 2019 May 24;89:123-127. doi: 10.1016/j.jbiomech.2019.04.012. Epub 2019 Apr 17. PMID: 31047695. | Excluded：review |
| 113 | Fatima ST, Jeilani A, Mazhar-ud-Duha, Abbasi NZ, Khan AA, Khan K, Sheikh AS, Ali F, Memon KH. Validation of tuning fork test in stress fractures and its comparison with radionuclide bone scan. J Ayub Med Coll Abbottabad. 2012 Jul-Dec;24(3-4):180-2. PMID: 24669648. | Excluded：review |
| 114 | Grant JA. Updating Recommendations for Rehabilitation after ACL Reconstruction: a Review. Clin J Sport Med. 2013 Nov;23(6):501-2. doi: 10.1097/JSM.0000000000000044. PMID: 24169301. | Excluded：review |
| 115 | Winnard A, Nasser M, Debuse D, Stokes M, Evetts S, Wilkinson M, Hides J, Caplan N. Systematic review of countermeasures to minimise physiological changes and risk of injury to the lumbopelvic area following long-term microgravity. Musculoskelet Sci Pract. 2017 Jan;27 Suppl 1:S5-S14. doi:10.1016/j.msksp.2016.12.009. Epub 2016 Dec 11. PMID: 28173932. | Excluded：review |
| 116 | Kitay GS, Koren MJ, Helfet DL, Parides MK, Markenson JA. Efficacy of combined local mechanical vibrations, continuous passive motion and thermotherapy in the management of osteoarthritis of the knee. Osteoarthritis  Cartilage. 2009 Oct;17(10):1269-74. doi: 10.1016/j.joca.2009.04.015. Epub 2009 May 4. PMID: 19433134. | Excluded：review |
| 117 | Culvenor AG, Girdwood MA, Juhl CB, Patterson BE, Haberfield MJ, Holm PM, Bricca A, Whittaker JL, Roos EM, Crossley KM. Rehabilitation after anterior cruciate ligament and meniscal injuries: a best-evidence synthesis of systematic reviews for the OPTIKNEE consensus. Br J Sports Med. 2022 Dec;56(24):1445-1453. doi: 10.1136/bjsports-2022-105495. Epub 2022 Jun 29. PMID: 35768181; PMCID:PMC9726950. | Excluded：review |
| 118 | Kluess D, Mittelmeier W, Bader R. Intraoperative impaction of total knee replacements: an explicit finite-element-analysis of principal stresses in ceramic vs. cobalt-chromium femoral components. Clin Biomech (Bristol, Avon). 2010 Dec;25(10):1018-24. doi: 10.1016/j.clinbiomech.2010.08.002. Epub 2010 Sep  15. PMID: 20828897. | Excluded：review |
| 119 | Martini E, Cesini I, D'Abbraccio J, Arnetoli G, Doronzio S, Giffone A, Meoni B, Oddo CM, Vitiello N, Crea S. Increased Symmetry of Lower-Limb Amputees Walking With Concurrent Bilateral Vibrotactile Feedback. IEEE Trans Neural Syst Rehabil Eng. 2021;29:74-84. doi: 10.1109/TNSRE.2020.3034521. Epub 2021 Feb 25. PMID: 33125331. | Excluded：review |
| 120 | Tsigonia A, Tanagra D, Linos A, Merekoulias G, Alexopoulos EC. Musculoskeletal disorders among cosmetologists. Int J Environ Res Public Health. 2009 Dec;6(12):2967-79. doi: 10.3390/ijerph6122967. Epub 2009 Nov 27. PMID: 20049238; PMCID: PMC2800326. | Excluded：review |
| 121 | Eckhardt H, Wollny R, Müller H, Bärtsch P, Friedmann-Bette B. Enhanced myofiber recruitment during exhaustive squatting performed as whole-body vibration exercise. J Strength Cond Res. 2011 Apr;25(4):1120-5. doi: 10.1519/JSC.0b013e3181d09e0e. PMID: 20647942. | Excluded：review |
| 122 | Jensen R, Kvale A, Baerheim A. Is pain in patellofemoral pain syndrome neuropathic? Clin J Pain. 2008 Jun;24(5):384-94. doi: 10.1097/AJP.0b013e3181658170. PMID: 18496302. | Excluded：review |
| 123 | Zhao Q, He Y, Wu N, Wang L, Dai J, Wang J, Ma J. Non-Pharmacological Interventions to Improve Physical Function in Patients with End-Stage Renal Disease: A Network Meta-Analysis. Am J Nephrol. 2023;54(1-2):35-41. doi:10.1159/000530219. Epub 2023 Mar 30. PMID: 36996785. | Excluded：review |
| 124 | Haas CT, Buhlmann A, Turbanski S, Schmidtbleicher D. Proprioceptive and sensorimotor performance in Parkinson's disease. Res Sports Med. 2006 Oct- Dec;14(4):273-87. doi: 10.1080/15438620600985902. PMID: 17214404. | Excluded：review |
| 125 | Fry AC, Housh TJ, Cramer JB, Weir JP, Beck TW, Schilling BK, Miller JD, Nicoll JX. Noninvasive Assessment of Skeletal Muscle Myosin Heavy Chain Expression in Trained and Untrained Men. J Strength Cond Res. 2017 Sep;31(9):2355-2362. doi: 10.1519/JSC.0000000000001645. PMID: 28820846. | Excluded：review |
| 126 | Peng X, Wang L. Idiopathic myelitis presenting as Brown-Séquard syndrome: two case reports and a review of the literature. J Med Case Rep. 2021 May 12;15(1):233. doi: 10.1186/s13256-021-02834-1. PMID: 33975651; PMCID:PMC8114540. | Excluded：review |
| 127 | Knee KM, Mukerji I. Real time monitoring of sickle cell hemoglobin fiber formation by UV resonance Raman spectroscopy. Biochemistry. 2009 Oct 20;48(41):9903-11. doi: 10.1021/bi901352m. PMID: 19778007. | Excluded：review |
| 128 | Mayr A. Investigating the Voce Faringea: Physiological and Acoustic Characteristics of the Bel Canto Tenor's Forgotten Singing Practice. J Voice. 2017 Mar;31(2):255.e13-255.e23. doi: 10.1016/j.jvoice.2016.06.010. Epub 2016 Jul  16. PMID: 27430862. | Excluded：review |
| 129 | McLaughlin JF, Felix SD, Nowbar S, Ferrel A, Bjornson K, Hays RM. Lower extremity sensory function in children with cerebral palsy. Pediatr Rehabil. 2005 Jan-Mar;8(1):45-52. doi: 10.1080/13638490400011181. PMID: 15799135. | Excluded：review |
| 130 | Murtezani A, Ibraimi Z, Sllamniku S, Osmani T, Sherifi S. Prevalence and risk factors for low back pain in industrial workers. Folia Med (Plovdiv). 2011 Jul-Sep;53(3):68-74. doi: 10.2478/v10153-011-0060-3. PMID: 22359986. | Excluded：review |
| 131 | Sonnery-Cottet B, Saithna A, Quelard B, Daggett M, Borade A, Ouanezar H, Thaunat M, Blakeney WG. Arthrogenic muscle inhibition after ACL reconstruction:  a scoping review of the efficacy of interventions. Br J Sports Med. 2019 Mar;53(5):289-298. doi: 10.1136/bjsports-2017-098401. Epub 2018 Sep 7. Erratum  in: Br J Sports Med. 2019 Dec;53(23):e8. doi: 10.1136/bjsports-2017-098401corr1. PMID: 30194224; PMCID: PMC6579490. | Excluded：review |
| 132 | Alkhuder K. Fourier-transform infrared spectroscopy: A universal optical sensing technique with auspicious application prospects in the diagnosis and management of autoimmune diseases. Photodiagnosis Photodyn Ther. 2023  Jun;42:103606. doi: 10.1016/j.pdpdt.2023.103606. Epub 2023 May 14. PMID:37187270. | Excluded：review |
| 133 | Poole CJ. Illness deception and work: incidence, manifestations and detection. Occup Med (Lond). 2010 Mar;60(2):127-32. doi: 10.1093/occmed/kqp170. Epub 2009 Dec 22. PMID: 20028802. | Excluded：review |
| 134 | Karki DB, Yadava SK, Pant S, Thusa N, Dangol E, Ghimire S. Prevalence of Sensory Neuropathy in Type 2 Diabetes Mellitus and Its Correlation with Duration of Disease. Kathmandu Univ Med J (KUMJ). 2016 Apr-Jun;14(54):120-124. PMID:  28166066. | Excluded：review |
| 135 | Tung TH, Martin DZ, Novak CB, Lauryssen C, Mackinnon SE. Nerve reconstruction in lumbosacral plexopathy. Case report and review of the literature. J Neurosurg. 2005 Jan;102(1 Suppl):86-91. doi: 10.3171/ped.2005.102.1.0086. PMID: 16206740. | Excluded：review |
| 136 | Shull PB, Damian DD. Haptic wearables as sensory replacement, sensory augmentation and trainer - a review. J Neuroeng Rehabil. 2015 Jul 20;12:59. doi: 10.1186/s12984-015-0055-z. PMID: 26188929; PMCID: PMC4506766. | Excluded：review |
| 137 | Bakola H, Zyga S, Stergioulas A, Kipreos G, Panoutsopoulos G. Musculoskeletal Problems Among Greek Perioperative Nurses in Regional Hospitals in Southern Peloponnese : Musculoskeletal Problems in Perioperative Nurses. Adv Exp Med Biol. 2017;989:21-37. doi: 10.1007/978-3-319-57348-9_3. PMID: 28971414. | Excluded：review |
| 138 | Joseph L, Standen M, Paungmali A, Kuisma R, Sitilertpisan P, Pirunsan U. Prevalence of musculoskeletal pain among professional drivers: A systematic review. J Occup Health. 2020 Jan;62(1):e12150. doi: 10.1002/1348-9585.12150.  PMID: 32810918; PMCID: PMC7434558. | Excluded：review |
| 139 | Hulshof CTJ, Pega F, Neupane S, van der Molen HF, Colosio C, Daams JG, Descatha A, Kc P, Kuijer PPFM, Mandic-Rajcevic S, Masci F, Morgan RL, Nygård CH, Oakman J, Proper KI, Solovieva S, Frings-Dresen MHW. The prevalence of occupational exposure to ergonomic risk factors: A systematic review and meta-analysis from the WHO/ILO Joint Estimates of the Work-related Burden of Disease and Injury. Environ Int. 2021 Jan;146:106157. doi: 10.1016/j.envint.2020.106157. Epub 2020 Dec 14. PMID: 33395953. | Excluded：review |
| 140 | Okita G, Hayashi M, Ikegami S, Iwakawa H, Haro H, Kato H. The Prevalence and Risk Factors for Trigger Digits in a Random Sampling of a Japanese Population Registry between 50 and 89 Years of Age. J Hand Surg Asian Pac Vol. 2022 Feb;27(1):148-155. doi: 10.1142/S2424835522500151. Epub 2022 Feb 9. PMID:  35135427. | Excluded：review |
| 141 | Farhadi F, Faraz M, Heng M, Johnson S. An Ergonomic Testing System for the First Metatarsophalangeal Joint Stiffness. J Biomech Eng. 2018 Oct 1;140(10). doi: 10.1115/1.4040248. PMID: 30029238. | Excluded：review |
| 142 | Bochkezanian V, Newton RU, Trajano GS, Vieira A, Pulverenti TS, Blazevich AJ. Effect of tendon vibration during wide-pulse neuromuscular electrical stimulation (NMES) on the decline and recovery of muscle force. BMC Neurol. 2017 May 2;17(1):82. doi: 10.1186/s12883-017-0862-x. PMID: 28464800; PMCID: PMC5414318. | Excluded：review |
| 143 | Biering-Sørensen F, Hansen B, Lee BS. Non-pharmacological treatment and prevention of bone loss after spinal cord injury: a systematic review. Spinal Cord. 2009 Jul;47(7):508-18. doi: 10.1038/sc.2008.177. Epub 2009 Jan 27. PMID: 19172152. | Excluded：review |
| 144 | Tampin B, Slater H, Jacques A, Lind CRP. Association of quantitative sensory testing parameters with clinical outcome in patients with lumbar radiculopathy undergoing microdiscectomy. Eur J Pain. 2020 Aug;24(7):1377-1392.  doi: 10.1002/ejp.1586. Epub 2020 Jun 14. PMID: 32383177; PMCID: PMC7496563. | Excluded：review |
| 145 | Johnson AW, Myrer JW, Hunter I, Feland JB, Hopkins JT, Draper DO, Eggett D. Whole-body vibration strengthening compared to traditional strengthening during physical therapy in individuals with total knee arthroplasty. Physiother Theory Pract. 2010 May;26(4):215-25. doi: 10.3109/09593980902967196. PMID: 20397856. | Excluded：review |
| 146 | Stewart S, Dalbeth N, Aiyer A, Rome K. Objectively Assessed Foot and Ankle Characteristics in Patients With Systemic Lupus Erythematosus: A Comparison With Age- and Sex-Matched Controls. Arthritis Care Res (Hoboken). 2020 Jan;72(1):122-130. doi: 10.1002/acr.23832. Epub 2019 Dec 10. PMID: 30629828. | Excluded：review |
| 147 | Hooftman WE, van der Beek AJ, Bongers PM, van Mechelen W. Is there a gender difference in the effect of work-related physical and psychosocial risk factors on musculoskeletal symptoms and related sickness absence? Scand J Work Environ Health. 2009 Mar;35(2):85-95. doi: 10.5271/sjweh.1316. Epub 2009 Apr 1. PMID: 19337673. | Excluded：review |
| 148 | Orlando G, Sacchetti M, D'Errico V, Haxhi J, Rapisarda G, Pugliese G, Balducci S. Muscle fatigability in patients with type 2 diabetes: relation with long-term complications. Diabetes Metab Res Rev. 2020 Feb;36(2):e3231. doi: 10.1002/dmrr.3231. Epub 2019 Nov 11. PMID: 31670895. | Excluded：review |
| 149 | Tan M, Urasawa K, Koshida R, Haraguchi T, Kitani S, Nakagawa Y, Igarashi Y, Sato K. Evaluation for the efficacy and safety of the crosser catheter as a CTO crossing device and a flossing device. Cardiovasc Interv Ther. 2018 Jan;33(1):77-83. doi: 10.1007/s12928-016-0444-7. Epub 2016 Nov 21. PMID: 27873169. | Excluded：review |
| 150 | Rosano C, Studenski SA, Aizenstein HJ, Boudreau RM, Longstreth WT Jr, Newman AB. Slower gait, slower information processing and smaller prefrontal area in older adults. Age Ageing. 2012 Jan;41(1):58-64. doi: 10.1093/ageing/afr113. Epub 2011 Sep 28. PMID: 21965414; PMCID: PMC3234076. | Excluded：review |
| 151 | He L, Van Roie E, Bogaerts A, Verschueren S, Delecluse C, Morse CI, Thomis M. The Genetic Effect on Muscular Changes in an Older Population: A Follow-Up Study after One-Year Cessation of Structured Training. Genes (Basel). 2020 Aug 21;11(9):968. doi: 10.3390/genes11090968. PMID: 32825595; PMCID: PMC7564970. | Excluded：review |
| 152 | Alexandrescu VA, Van Overmeire L, Makrygiannis G, Azdad K, Popitiu M, Paquet S, Poppe L, Nodit M. Clinical Implications of Diabetic Peripheral Neuropathy in Primary Infrapopliteal Angioplasty Approach for Neuro-Ischemic  Foot Wounds. J Endovasc Ther. 2023 Dec;30(6):920-930. doi: 10.1177/15266028221106312. Epub 2022 Jul 2. PMID: 35786131. | Excluded：review |
| 153 | Roquelaure Y, Ha C, Rouillon C, Fouquet N, Leclerc A, Descatha A, Touranchet A, Goldberg M, Imbernon E; Members of Occupational Health Services of the Pays de la Loire Region. Risk factors for upper-extremity musculoskeletal disorders in the working population. Arthritis Rheum. 2009 Oct 15;61(10):1425-34. doi: 10.1002/art.24740. PMID: 19790112; PMCID: PMC3189514. | Excluded：review |
| 154 | Chen B, Chen C, Hu J, Sayeed Z, Qi J, Darwiche HF, Little BE, Lou S, Darwish M, Foote C, Palacio-Lascano C. Computer Vision and Machine Learning- Based Gait Pattern Recognition for Flat Fall Prediction. Sensors (Basel). 2022 Oct 19;22(20):7960. doi: 10.3390/s22207960. PMID: 36298311; PMCID: PMC9612353. | Excluded：review |
| 155 | Shiri R, Falah-Hassani K, Heliövaara M, Solovieva S, Amiri S, Lallukka T, Burdorf A, Husgafvel-Pursiainen K, Viikari-Juntura E. Risk Factors for Low Back Pain: A Population-Based Longitudinal Study. Arthritis Care Res (Hoboken). 2019 Feb;71(2):290-299. doi: 10.1002/acr.23710. PMID: 30044543. | Excluded：review |
| 156 | Parsons V, Pattani S, Gilbey A, Madan I, Harling C, Muiry R, de Bono A. Expanding the strategic and clinical leadership role of accredited specialists. Occup Med (Lond). 2022 Oct 18;72(7):456-461. doi: 10.1093/occmed/kqac064. PMID:35815920. | Excluded：review |
| 157 | Wang TG, Hsiao TY, Wang CL, Shau YW. Resonance frequency in patellar tendon. Scand J Med Sci Sports. 2007 Oct;17(5):535-8. doi: 10.1111/j.1600-0838.2006.00618.x. Epub 2007 Feb 19. PMID: 17316374. | Excluded：review |
| 158 | Yang F, Di N, Guo WW, Ding WB, Jia N, Zhang H, Li D, Wang D, Wang R, Zhang D, Liu Y, Shen B, Wang ZX, Yin Y. The prevalence and risk factors of work related musculoskeletal disorders among electronics manufacturing workers: a cross-sectional analytical study in China. BMC Public Health. 2023 Jan 3;23(1):10. doi: 10.1186/s12889-022-14952-6. PMID: 36597111; PMCID: PMC9809125. | Excluded：review |
| 159 | Yamada K, Yuan J, Mano T, Takashima H, Shibata M. Arthropathy-related pain in a patient with congenital impairment of pain sensation due to hereditary sensory and autonomic neuropathy type II with a rare mutation in the WNK1/HSN2 gene: a case report. BMC Neurol. 2016 Oct 21;16(1):201. doi: 10.1186/s12883-016-0727-8. PMID: 27765018; PMCID: PMC5073964. | Excluded：review |
| 160 | Howlin RP, Fabbri S, Offin DG, Symonds N, Kiang KS, Knee RJ, Yoganantham DC, Webb JS, Birkin PR, Leighton TG, Stoodley P. Removal of Dental Biofilms with an Ultrasonically Activated Water Stream. J Dent Res. 2015 Sep;94(9):1303-9. doi: 10.1177/0022034515589284. Epub 2015 Jun 8. PMID: 26056055. | Excluded：review |
| 161 | French HP, Abbott JH, Galvin R. Adjunctive therapies in addition to land-based exercise therapy for osteoarthritis of the hip or knee. Cochrane Database Syst Rev. 2022 Oct 17;10(10):CD011915.doi: 10.1002/14651858.CD011915.pub2. PMID: 36250418; PMCID: PMC9574868. | Excluded：review |
| 162 | Ingram TG, Roddick JM, Byrne JM. Is gamma loop dysfunction related to bilateral inhibition in anterior knee pain? Muscle Nerve. 2016 Feb;53(2):280-6. doi: 10.1002/mus.24705. Epub 2015 Nov 26. PMID: 25974873. | Excluded：review |
| 163 | Lin WC, Lee CL, Chang NJ. Acute Effects of Dynamic Stretching Followed by Vibration Foam Rolling on Sports Performance of Badminton Athletes. J Sports Sci Med. 2020 May 1;19(2):420-428. PMID: 32390736; PMCID: PMC7196741. | Excluded：review |
| 164 | Hosu CD, Moisoiu V, Stefancu A, Antonescu E, Leopold LF, Leopold N, Fodor D. Raman spectroscopy applications in rheumatology. Lasers Med Sci. 2019 Jun;34(4):827-834. doi: 10.1007/s10103-019-02719-2. Epub 2019 Jan 21. PMID: 30666523. | Excluded：review |
| 165 | Wang Z, Zhang X, Sun M. The application of whole-body vibration training in knee osteoarthritis. Joint Bone Spine. 2022 Mar;89(2):105276. doi: 10.1016/j.jbspin.2021.105276. Epub 2021 Sep 15. PMID: 34536625. | Excluded：review |
| 166 | Montagnese F, Thiele S, Wenninger S, Schoser B. Long-term whole-body vibration training in two late-onset Pompe disease patients. Neurol Sci. 2016 Aug;37(8):1357-60. doi: 10.1007/s10072-016-2612-z. Epub 2016 May 18. PMID:  27193587. | Excluded：review |
| 167 | Ye Y, Wan Z, Liu B, Xu H, Wang Q, Ding T. Monitoring deterioration of knee osteoarthritis using vibration arthrography in daily activities. Comput Methods Programs Biomed. 2022 Jan;213:106519. doi: 10.1016/j.cmpb.2021.106519. Epub 2021 Nov 12. PMID: 34826659. | Excluded：review |
| 168 | Bennell KL, Hall M, Hinman RS. Osteoarthritis year in review 2015: rehabilitation and outcomes. Osteoarthritis Cartilage. 2016 Jan;24(1):58-70. doi: 10.1016/j.joca.2015.07.028. PMID: 26707993. | Excluded：review |
| 169 | Lu Y, Zhang J, Li H, Li T. Association of non-alcoholic fatty liver disease with self-reported osteoarthritis among the US adults. Arthritis Res Ther. 2024 Jan 31;26(1):40. doi: 10.1186/s13075-024-03272-2. PMID: 38297351; PMCID:  PMC10829206. | Excluded：review |
| 170 | Takanashi Y, Chinen Y, Hatakeyama S. Whole-body vibration training improves the balance ability and leg strength of athletic throwers. J Sports Med Phys Fitness. 2019 Jul;59(7):1110-1118. doi: 10.23736/S0022-4707.18.09012-6. Epub 2018 Oct 10. PMID: 30317837. | Excluded：review |
| 171 | Blackburn T, Padua DA, Pietrosimone B, Schwartz TA, Spang JT, Goodwin JS, Dewig DR, Johnston CD. Vibration improves gait biomechanics linked to posttraumatic knee osteoarthritis following anterior cruciate ligament injury. J  Orthop Res. 2021 May;39(5):1113-1122. doi: 10.1002/jor.24821. Epub 2020 Aug 12. PMID: 32757272. | Excluded：review |
| 172 | Aminian-Far A, Hadian MR, Olyaei G, Talebian S, Bakhtiary AH. Whole-body vibration and the prevention and treatment of delayed-onset muscle soreness. J Athl Train. 2011 Jan-Feb;46(1):43-9. doi: 10.4085/1062-6050-46.1.43. PMID:21214349; PMCID: PMC3017487. | Excluded：review |
| 173 | Templeton CA, Strzalkowski NDJ, Galvin P, Bent LR. Cutaneous sensitivity in unilateral trans-tibial amputees. PLoS One. 2018 Jun 1;13(6):e0197557. doi: 10.1371/journal.pone.0197557. PMID: 29856766; PMCID: PMC5983436. | Excluded：review |
| 174 | Andreu-Caravaca L, Chung LH, Ramos-Campo DJ, Marín-Cascales E, Encarnación-Martínez A, Rubio-Arias JÁ. Neuromuscular and Mobility Responses to a Vibration Session in Hypoxia in Multiple Sclerosis. Int J Sports Med. 2021 Apr;42(4):307-313. doi: 10.1055/a-1273-8304. Epub 2020 Oct 19. PMID: 33075829. | Excluded：review |
| 175 | Viellehner J, Potthast W. The effect of vibration on kinematics and muscle activation during cycling. J Sports Sci. 2022 Aug;40(15):1760-1771. doi: 10.1080/02640414.2022.2109841. Epub 2022 Aug 19. PMID: 35984289. | Excluded：review |
| 176 | Yoon J, Kanamori A, Fujii K, Isoda H, Okura T. Evaluation of maslinic acid with whole-body vibration training in elderly women with knee osteoarthritis. PLoS One. 2018 Mar 20;13(3):e0194572. doi: 10.1371/journal.pone.0194572. PMID: 29558490; PMCID: PMC5860762. | Excluded：review |
| 177 | Chuang LR, Yang WW, Chang PL, Chen VC, Liu C, Shiang TY. Managing Vibration Training Safety by Using Knee Flexion Angle and Rating Perceived Exertion. Sensors (Basel). 2021 Feb 7;21(4):1158. doi: 10.3390/s21041158. PMID: 33562177; PMCID: PMC7915332. | Excluded：review |
| 178 | Ferreira RM, Duarte JA, Gonçalves RS. Non-pharmacological and non-surgical interventions to manage patients with knee osteoarthritis: An umbrella review. Acta Reumatol Port. 2018 Jul-Sep;43(3):182-200. English. PMID: 30414367. | Excluded：review |
| 179 | Chaltron C, Sherman DA, Pamukoff DN, Bazett-Jones DM, Glaviano NR, Norte GE. Whole-body vibration reduces hamstrings neuromuscular function in uninjured individuals. Phys Ther Sport. 2023 Mar;60:17-25. doi: 10.1016/j.ptsp.2023.01.004. Epub 2023 Jan 9. PMID: 36640639. | Excluded：review |
| 180 | Paolucci T, Agostini F, Bernetti A, Paoloni M, Mangone M, Santilli V, Pezzi L, Bellomo RG, Saggini R. Integration of focal vibration and intra-articular oxygen-ozone therapy in rehabilitation of painful knee osteoarthritis. J Int Med Res. 2021 Feb;49(2):300060520986705. doi: 10.1177/0300060520986705. PMID: 33641438; PMCID: PMC7923992. | Excluded：review |
| 181 | Betik AC, Parker L, Kaur G, Wadley GD, Keske MA. Whole-Body Vibration Stimulates Microvascular Blood Flow in Skeletal Muscle. Med Sci Sports Exerc. 2021 Feb 1;53(2):375-383. doi: 10.1249/MSS.0000000000002463. PMID: 32826637. | Excluded：review |
| 182 | Abercromby AF, Amonette WE, Layne CS, McFarlin BK, Hinman MR, Paloski WH. Vibration exposure and biodynamic responses during whole-body vibration training. Med Sci Sports Exerc. 2007 Oct;39(10):1794-800. doi:10.1249/mss.0b013e3181238a0f. PMID: 17909407. | Excluded：review |
| 183 | Bergmann G, Kutzner I, Bender A, Dymke J, Trepczynski A, Duda GN, Felsenberg D, Damm P. Loading of the hip and knee joints during whole body vibration training. PLoS One. 2018 Dec 12;13(12):e0207014. doi: 10.1371/journal.pone.0207014. PMID: 30540775; PMCID: PMC6291191. | Excluded：review |
| 184 | Zhang J, Wang R, Zheng Y, Xu J, Wu Y, Wang X. Effect of Whole-Body Vibration Training on Muscle Activation for Individuals with Knee Osteoarthritis. Biomed Res Int. 2021 Mar 26;2021:6671390. doi: 10.1155/2021/6671390. PMID: 33855078; PMCID: PMC8019384. | Excluded：review |
| 185 | Gainutdinovs O, Gaynutdinov O, Jevstignejevs V, Studers P. Vibration transmission in bone-anchored prosthesis under vertical load. Cadaver study. Prosthet Orthot Int. 2022 Oct 1;46(5):444-451. doi: 10.1097/PXR.0000000000000127. Epub 2022 Apr 1. PMID: 35363637. | Excluded：review |
| 186 | Shadloo N, Kamali F, Salehi Dehno N. A comparison between whole-body vibration and conventional training on pain and performance in athletes with patellofemoral pain. J Bodyw Mov Ther. 2021 Jul;27:661-666. doi: 10.1016/j.jbmt.2021.03.003. Epub 2021 Mar 11. PMID: 34391303. | Excluded：review |
| 187 | Eloá MM, Carla F D, Danielle S M, Danubia C SC, Cintia R SG, Laisa L PD, Ygor TS, Mario José Dos Santos. Whole body vibration and auriculotherapy improve handgrip strength in individuals with knee osteoarthritis. J Tradit Chin Med. 2019 Oct;39(5):707-715. PMID: 32186121. | Excluded：review |
| 188 | Pan T, Zhang Y, Dong Q, Ye Y, Li Y, Wan Z, Ding T. Vibroarthrography-based Knee Lesions Location via Multi-Label Embedding Learning. Annu Int Conf IEEE Eng Med Biol Soc. 2023 Jul;2023:1-4. doi: 10.1109/EMBC40787.2023.10340411. PMID: 38083623. | Excluded：review |
| 189 | Sogut B, Ozsoy H, Baloglu R, Harput G. Effects of Whole-Body Vibration Training on Knee Muscle Strength After Anterior Cruciate Ligament Reconstruction: A Critically Appraised Topic. J Sport Rehabil. 2022 Mar 1;31(3):356-361. doi: 10.1123/jsr.2021-0210. Epub 2021 Oct 25. PMID: 34697253. | Excluded：review |
| 190 | Salmon JR, Roper JA, Tillman MD. Does acute whole-body vibration training improve the physical performance of people with knee osteoarthritis? J Strength Cond Res. 2012 Nov;26(11):2983-9. doi: 10.1519/JSC.0b013e318242a4be. PMID: 22130389. | Excluded：review |
| 191 | Lluch E, Nijs J, Courtney CA, Rebbeck T, Wylde V, Baert I, Wideman TH, Howells N, Skou ST. Clinical descriptors for the recognition of central sensitization pain in patients with knee osteoarthritis. Disabil Rehabil. 2018 Nov;40(23):2836-2845. doi: 10.1080/09638288.2017.1358770. Epub 2017 Aug 2. PMID: 28768437. | Excluded：review |
| 192 | Yu PM, Lin Y, Zhang C, Wang HM, Wei Q, Zhu SY, Wei QC, Wang ZG, Pan HX, Huang RD, He CQ. Low-Frequency Vibration Promotes Tumor Necrosis Factor-α  Production to Increase Cartilage Degeneration in Knee Osteoarthritis. Cartilage. 2021 Dec;13(2_suppl):1398S-1406S. doi: 10.1177/1947603520931178. Epub 2020 Jun  12. PMID: 32532183; PMCID: PMC8804826. | Excluded：review |
| 193 | Karatrantou K, Gerodimos V, Dipla K, Zafeiridis A. Whole-body vibration training improves flexibility, strength profile of knee flexors, and hamstrings- to-quadriceps strength ratio in females. J Sci Med Sport. 2013 Sep;16(5):477-81.  doi: 10.1016/j.jsams.2012.11.888. Epub 2012 Dec 17. PMID: 23253266. | Excluded：review |
| 194 | Harden RN, Wallach G, Gagnon CM, Zereshki A, Mukai A, Saracoglu M, Kuroda MM, Graciosa JR, Bruehl S. The osteoarthritis knee model: psychophysical characteristics and putative outcomes. J Pain. 2013 Mar;14(3):281-9. doi:  10.1016/j.jpain.2012.11.009. Epub 2013 Feb 4. PMID: 23380268. | Excluded：review |
| 195 | Hannah R, Minshull C, Folland JP. Whole-body vibration does not influence knee joint neuromuscular function or proprioception. Scand J Med Sci Sports. 2013 Feb;23(1):96-104. doi: 10.1111/j.1600-0838.2011.01361.x. Epub 2011 Aug 8. PMID: 21819446. | Excluded：review |
| 196 | Safaei M, Bolus NB, Erturk A, Inan OT. Vibration Characterization of the Human Knee Joint in Audible Frequencies. Sensors (Basel). 2020 Jul 25;20(15):4138. doi: 10.3390/s20154138. PMID: 32722389; PMCID: PMC7436205. | Excluded：review |
| 197 | Landsbergis P, Johanning E, Stillo M, Jain R, Davis M. Occupational risk factors for musculoskeletal disorders among railroad maintenance-of-way workers. Am J Ind Med. 2020 May;63(5):402-416. doi: 10.1002/ajim.23099. Epub 2020 Mar 7. PMID: 32144807. | Excluded：review |
| 198 | Moreira D, Silva J, Correia MV, Massada M. Classification of knee arthropathy with accelerometer-based vibroarthrography. Stud Health Technol Inform. 2016;224:33-9. PMID: 27225550. | Excluded：review |
| 199 | Pleguezuelos E, Pérez ME, Guirao L, Samitier B, Costea M, Ortega P, González MV, Del Carmen VA, Ovejero L, Moreno E, Miravitlles M. Effects of whole body vibration training in patients with severe chronic obstructive pulmonary disease. Respirology. 2013 Aug;18(6):1028-34. doi: 10.1111/resp.12122. PMID: 23692550. | Excluded：review |
| 200 | Roggio F, Trovato B, Ledda C, Rapisarda V, Musumeci G. Kinesiological Treatment of Early Spine Osteoarthritis in a Motorcyclist. Int J Environ Res Public Health. 2022 Jan 15;19(2):961. doi: 10.3390/ijerph19020961. PMID: 35055784; PMCID: PMC8776179. | Excluded：review |
| 201 | Safaei M, Bolus NB, Whittingslow DC, Jeong HK, Erturk A, Inan OT. Vibration Stimulation as a Non-Invasive Approach to Monitor the Severity of Meniscus Tears. IEEE Trans Neural Syst Rehabil Eng. 2021;29:350-359. doi: 10.1109/TNSRE.2021.3050439. Epub 2021 Mar 2. PMID: 33428572. | Excluded：review |
| 202 | Chadefaux D, Moorhead AP, Marzaroli P, Marelli S, Marchetti E, Tarabini M. Vibration transmissibility and apparent mass changes from vertical whole-body vibration exposure during stationary and propelled walking. Appl Ergon. 2021 Jan;90:103283. doi:10.1016/j.apergo.2020.103283. Epub 2020 Oct 10. PMID:33049546. | Excluded：review |
| 203 | Karacan I, Cidem M, Yilmaz G, Sebik O, Cakar HI, Türker KS. Tendon reflex is suppressed during whole-body vibration. J Electromyogr Kinesiol. 2016 Oct;30:191-5. doi: 10.1016/j.jelekin.2016.07.008. Epub 2016 Jul 25. PMID: 27485766. | Excluded：review |
| 204 | Segal NA, Glass NA, Shakoor N, Wallace R. Vibration platform training in women at risk for symptomatic knee osteoarthritis. PM R. 2013 Mar;5(3):201-9; quiz 209. doi: 10.1016/j.pmrj.2012.07.011. Epub 2012 Sep 12. PMID: 22981005; PMCID: PMC3838794. | Excluded：review |
| 205 | Krafft C, Steiner G, Beleites C, Salzer R. Disease recognition by infrared and Raman spectroscopy. J Biophotonics. 2009 Feb;2(1-2):13-28. doi: 10.1002/jbio.200810024. PMID: 19343682. | Excluded：review |
| 206 | Taşcılar LN, Kaya Utlu D, Sayaca Ç, Polat G, Kuyucu E, Erdil ME. Is plantar foot sensation affected in patients with gonarthrosis. Acta Orthop Traumatol Turc. 2021 Dec;55(6):518-526. doi: 10.5152/j.aott.2021.21213. PMID: 34967741. | Excluded：review |
| 207 | Hammer PE, Shiri R, Kryger AI, Kirkeskov L, Bonde JP. Associations of work activities requiring pinch or hand grip or exposure to hand-arm vibration with finger and wrist osteoarthritis: a meta-analysis. Scand J Work Environ Health. 2014 Mar;40(2):133-45. doi: 10.5271/sjweh.3409. Epub 2013 Dec 5. PMID: 24310528. | Excluded：review |
| 208 | Menéndez H, Martín-Hernández J, Ferrero C, Figueroa A, Herrero AJ, Marín PJ. Influence of isolated or simultaneous application of electromyostimulation and vibration on leg blood flow. Eur J Appl Physiol. 2015 Aug;115(8):1747-55. doi: 10.1007/s00421-015-3161-5. Epub 2015 Mar 29. PMID: 25820213. | Excluded：review |
| 209 | Martínez A, Lam CK, von Tscharner V, Nigg BM. Soft tissue vibration dynamics after an unexpected impact. Physiol Rep. 2019 Jan;7(2):e13990. doi: 10.14814/phy2.13990. PMID: 30659770; PMCID: PMC6339545. | Excluded：review |
| 210 | Artero EG, Espada-Fuentes JC, Argüelles-Cienfuegos J, Román A, Gómez-López PJ, Gutiérrez A. Effects of whole-body vibration and resistance training on knee extensors muscular performance. Eur J Appl Physiol. 2012 Apr;112(4):1371-8. doi: 10.1007/s00421-011-2091-0. Epub 2011 Aug 2. PMID: 21809090. | Excluded：review |
| 211 | Charlton JM, Xia H, Shull PB, Eng JJ, Li LC, Hunt MA. Multi-day monitoring of foot progression angles during unsupervised, real-world walking in people with and without knee osteoarthritis. Clin Biomech (Bristol, Avon). 2023 May;105:105957. doi: 10.1016/j.clinbiomech.2023.105957. Epub 2023 Apr 14. PMID: 37084548. | Excluded：review |
| 212 | Kumar R, Grønhaug KM, Afseth NK, Isaksen V, de Lange Davies C, Drogset JO, Lilledahl MB. Optical investigation of osteoarthritic human cartilage (ICRS grade) by confocal Raman spectroscopy: a pilot study. Anal Bioanal Chem. 2015 Oct;407(26):8067-77. doi: 10.1007/s00216-015-8979-5. Epub 2015 Aug 29. PMID:26319282. | Excluded：review |
| 213 | Cai S, Wu Y, Xiang N, Zhong Z, He J, Shi L, Xu F. Detrending knee joint vibration signals with a cascade moving average filter. Annu Int Conf IEEE Eng Med Biol Soc. 2012;2012:4357-60. doi: 10.1109/EMBC.2012.6346931. PMID: 23366892. | Excluded：review |
| 214 | Reiner MM, Tilp M, Guilhem G, Morales-Artacho A, Konrad A. Comparison of A Single Vibration Foam Rolling and Static Stretching Exercise on the Muscle Function and Mechanical Properties of the Hamstring Muscles. J Sports Sci Med. 2022 Jun 1;21(2):287-297. doi: 10.52082/jssm.2022.287. PMID: 35719228; PMCID: PMC9157528. | Excluded：review |
| 215 | Blackburn JT, Pietrosimone B, Spang JT, Goodwin JS, Johnston CD. Somatosensory Function Influences Aberrant Gait Biomechanics Following Anterior Cruciate Ligament Reconstruction. J Orthop Res. 2020 Mar;38(3):620-628. doi: 10.1002/jor.24495. Epub 2019 Oct 24. PMID: 31608488. | Excluded：review |
| 216 | Tseng SY, Lai CL, Ko CP, Chang YK, Fan HC, Wang CH. The Effectiveness of Whole-Body Vibration and Heat Therapy on the Muscle Strength, Flexibility, and Balance Abilities of Elderly Groups. Int J Environ Res Public Health. 2023 Jan 16;20(2):1650. doi: 10.3390/ijerph20021650. PMID: 36674404; PMCID: PMC9861224. | Excluded：review |
| 217 | Hulshof CTJ, Pega F, Neupane S, Colosio C, Daams JG, Kc P, Kuijer PPFM, Mandic-Rajcevic S, Masci F, van der Molen HF, Nygård CH, Oakman J, Proper KI, Frings-Dresen MHW. The effect of occupational exposure to ergonomic risk factors on osteoarthritis of hip or knee and selected other musculoskeletal diseases: A systematic review and meta-analysis from the WHO/ILO Joint Estimates of the Work-related Burden of Disease and Injury. Environ Int. 2021 May;150:106349. doi: 10.1016/j.envint.2020.106349. Epub 2021 Feb 3. PMID: 33546919. | Excluded：review |
| 218 | Nakamura M, Konrad A, Kasahara K, Yoshida R, Murakami Y, Sato S, Aizawa K, Koizumi R, Wilke J. The Combined Effect of Static Stretching and Foam Rolling With or Without Vibration on the Range of Motion, Muscle Performance, and Tissue Hardness of the Knee Extensor. J Strength Cond Res. 2023 Feb 1;37(2):322-327. doi: 10.1519/JSC.0000000000004263. Epub 2022 May 9. PMID: 35544351; PMCID: PMC7614110. | Excluded：review |
| 219 | Prioreschi A, Makda MA, Tikly M, McVeigh JA. In Patients with Established RA, Positive Effects of a Randomised Three Month WBV Therapy Intervention on  Functional Ability, Bone Mineral Density and Fatigue Are Sustained for up to Six Months. PLoS One. 2016 Apr 13;11(4):e0153470. doi: 10.1371/journal.pone.0153470.  PMID: 27073832; PMCID: PMC4830593. | Excluded：review |
| 220 | Arami A, Delaloye JR, Rouhani H, Jolles BM, Aminian K. Knee Implant Loosening Detection: A Vibration Analysis Investigation. Ann Biomed Eng. 2018 Jan;46(1):97-107. doi: 10.1007/s10439-017-1941-2. Epub 2017 Oct 24. PMID: 29067562; PMCID: PMC5754435. | Excluded：review |
| 221 | Picot B, Lempereur M, Morel B, Forestier N, Rémy-Néris O. Lack of Proprioceptive Strategy Modulation Leads to At-Risk Biomechanics for Anterior Cruciate Ligament in Healthy Athletes. Med Sci Sports Exerc. 2024 May 1;56(5):942-952. doi: 10.1249/MSS.0000000000003378. Epub 2024 Jan 8. PMID:38190373. | Excluded：review |
| 222 | Fischer AG, Erhart-Hledik JC, Asay JL, Chu CR, Andriacchi TP. Activating the somatosensory system enhances net quadriceps moment during gait. J Biomech. 2019 Jan 3;82:149-155. doi: 10.1016/j.jbiomech.2018.10.026. Epub 2018 Oct 26. PMID: 30381155. | Excluded：review |
| 223 | Łysiak A, Froń A, Bączkowicz D, Szmajda M. Vibroarthrographic Signal Spectral Features in 5-Class Knee Joint Classification. Sensors (Basel). 2020 Sep 3;20(17):5015. doi: 10.3390/s20175015. PMID: 32899440; PMCID: PMC7506694. | Excluded：review |
| 224 | Sañudo B, Feria A, Carrasco L, de Hoyo M, Santos R, Gamboa H. Does whole body vibration training affect knee kinematics and neuromuscular control in healthy people? J Sports Sci. 2012;30(14):1537-44. doi: 10.1080/02640414.2012.713503. Epub 2012 Aug 16. PMID: 22894146. | Excluded：review |
| 225 | Rieder F, Wiesinger HP, Kösters A, Müller E, Seynnes OR. Whole-body vibration training induces hypertrophy of the human patellar tendon. Scand J Med Sci Sports. 2016 Aug;26(8):902-10. doi: 10.1111/sms.12522. Epub 2015 Jul 15. PMID: 26173589. | Excluded：review |
| 226 | Clarke-Brodber AL, Taxy JB. The Stapes in Otosclerosis: Osteoarthritis of an Ear Ossicle. Head Neck Pathol. 2021 Sep;15(3):737-742. doi: 10.1007/s12105-020-01269-2. Epub 2021 Jan 7. PMID: 33415516; PMCID: PMC8384928. | Excluded：review |
| 227 | Chwała W, Pogwizd P. Effects of vibration and passive resting on muscle stiffness and restitution after submaximal exercise analyzed by elastography. Acta Bioeng Biomech. 2021;23(2):3-11. PMID: 34846035. | Excluded：review |
| 228 | Lee J, Lee K, Song C. Determining the Posture and Vibration Frequency that Maximize Pelvic Floor Muscle Activity During Whole-Body Vibration. Med Sci Monit. 2016 Oct 27;22:4030-4036. doi: 10.12659/msm.898011. PMID: 27787476; PMCID: PMC5087668. | Excluded：review |
| 229 | Wadsworth D, Turnbull J, Lark S. Psychological Effects of Whole-Body Vibration Training in Frail Older Adults: An Open, Randomized Control Trial. J Aging Phys Act. 2022 Feb 1;30(1):54-64. doi: 10.1123/japa.2020-0400. Epub 2021 Aug 4. PMID: 34348227. | Excluded：review |
| 230 | Elia A, Eiken O, Ånell R, Grönkvist M, Gennser M. Whole-body vibration preconditioning reduces the formation and delays the manifestation of high- altitude-induced venous gas emboli. Exp Physiol. 2021 Aug;106(8):1743-1751. doi:  10.1113/EP089522. Epub 2021 Jun 28. PMID: 34142740. | Excluded：review |
| 231 | Nawayseh N. Transmission of vibration from a vibrating plate to the head of standing people. Sports Biomech. 2019 Oct;18(5):482-500. doi: 10.1080/14763141.2018.1434233. Epub 2018 Mar 20. PMID: 29558238. | Excluded：review |
| 232 | Kalo K, Niederer D, Sus R, Sohrabi K, Groß V, Vogt L. Reliability of Vibroarthrography to Assess Knee Joint Sounds in Motion. Sensors (Basel). 2020 Apr 2;20(7):1998. doi: 10.3390/s20071998. PMID: 32252480; PMCID: PMC7181296. | Excluded：review |
| 233 | Liao LR, Ng GY, Jones AY, Huang MZ, Pang MY. Whole-Body Vibration Intensities in Chronic Stroke: A Randomized Controlled Trial. Med Sci Sports Exerc. 2016 Jul;48(7):1227-38. doi: 10.1249/MSS.0000000000000909. PMID: 26918558. | Excluded：review |
| 234 | Hnat SK, van Basten BJH, van den Bogert AJ. Compensation for inertial and gravity effects in a moving force platform. J Biomech. 2018 Jun 25;75:96-101. doi: 10.1016/j.jbiomech.2018.05.009. Epub 2018 May 19. PMID: 29789150. | Excluded：review |
| 235 | Condemi S, Panuel M, Chaumoitre K, Belcastro MG, Pietrobelli A, Voisin JL. A pathological Neandertal thumb phalanx from Moula-Guercy (France). Int J Paleopathol. 2023 Sep;42:14-17. doi: 10.1016/j.ijpp.2023.06.002. Epub 2023 Jun 22. PMID: 37354658. | Excluded：review |
| 236 | Luo X, Zhang J, Zhang C, He C, Wang P. The effect of whole-body vibration therapy on bone metabolism, motor function, and anthropometric parameters in women with postmenopausal osteoporosis. Disabil Rehabil. 2017 Nov;39(22):2315-2323. doi: 10.1080/09638288.2016.1226417. Epub 2016 Oct 10. PMID: 27718643. | Excluded：review |
| 237 | Kasahara K, Konrad A, Yoshida R, Murakami Y, Koizumi R, Sato S, Ye X, Thomas E, Nakamura M. Comparison of the Prolonged Effects of Foam Rolling and Vibration Foam Rolling Interventions on Passive Properties of Knee Extensors. J Sports Sci Med. 2022 Dec 1;21(4):580-585. doi: 10.52082/jssm.2022.580. PMID: 36523900; PMCID: PMC9741721. | Excluded：review |
| 238 | Beerse M, Lelko M, Wu J. Acute effect of whole-body vibration on acceleration transmission and jumping performance in children. Clin Biomech (Bristol, Avon). 2021 Jan;81:105235. doi: 10.1016/j.clinbiomech.2020.105235. Epub 2020 Nov 14. PMID: 33221052. | Excluded：review |
| 239 | Masani K, Alizadeh-Meghrazi M, Sayenko DG, Zariffa J, Moore C, Giangregorio L, Popovic MR, Catharine Craven B. Muscle activity, cross-sectional area, and density following passive standing and whole body vibration: A case series. J Spinal Cord Med. 2014 Sep;37(5):575-81. doi: 10.1179/2045772314Y.0000000255. Epub 2014 Jul 24. PMID: 25059652; PMCID: PMC4166192. | Excluded：review |
| 240 | Rieder F, Wiesinger HP, Kösters A, Müller E, Seynnes OR. Immediate effects of whole body vibration on patellar tendon properties and knee extension torque. Eur J Appl Physiol. 2016 Mar;116(3):553-61. doi: 10.1007/s00421-015-3316-4. Epub 2015 Dec 26. PMID: 26708361. | Excluded：review |
| 241 | Hardin EC, Bailey SN, Kobetic R, Lombardo LM, Foglyano KM, Schnellenberger JR, Selkirk SM. Development and deployment of cyclical focal muscle vibration system to improve walking performance in multiple sclerosis. J Med Eng Technol. 2022 Jul;46(5):393-401. doi: 10.1080/03091902.2022.2080880. Epub 2022 Jun 8. PMID: 35674709. | Excluded：review |
| 242 | Spain L, Yang L, Wilkinson JM, McCloskey E. Transmission of whole body vibration - Comparison of three vibration platforms in healthy subjects. Bone. 2021 Mar;144:115802. doi: 10.1016/j.bone.2020.115802. Epub 2020 Dec 10. PMID: 33309990. | Excluded：review |
| 243 | Shull PB, Jirattigalachote W, Hunt MA, Cutkosky MR, Delp SL. Quantified self and human movement: a review on the clinical impact of wearable sensing and feedback for gait analysis and intervention. Gait Posture. 2014;40(1):11-9. doi: 10.1016/j.gaitpost.2014.03.189. Epub 2014 Apr 6. PMID: 24768525. | Excluded：review |
| 244 | Liphardt AM, Schipilow J, Hanley DA, Boyd SK. Bone quality in osteopenic postmenopausal women is not improved after 12 months of whole-body vibration training. Osteoporos Int. 2015 Mar;26(3):911-20. doi: 10.1007/s00198-014-2995-8. Epub 2015 Jan 8. PMID: 25567775. | Excluded：review |
| 245 | Befrui N, Elsner J, Flesser A, Huvanandana J, Jarrousse O, Le TN, Müller M, Schulze WHW, Taing S, Weidert S. Vibroarthrography for early detection of knee osteoarthritis using normalized frequency features. Med Biol Eng Comput. 2018 Aug;56(8):1499-1514. doi: 10.1007/s11517-018-1785-4. Epub 2018 Feb 1. PMID: 29392547. | Excluded：review |
| 246 | Chen CH, Chiu CH, Tseng WC, Wu CY, Su HH, Chang CK, Ye X. Acute Effects of Combining Dynamic Stretching and Vibration Foam Rolling Warm-up on Lower-Limb Muscle Performance and Functions in Female Handball Players. J Strength Cond Res. 2023 Jun 1;37(6):1277-1283. doi: 10.1519/JSC.0000000000003998. Epub 2023 Feb 15. PMID: 33651738. | Excluded：review |
| 247 | Nakamura M, Kasahara K, Yoshida R, Murakami Y, Koizumi R, Sato S, Takeuchi K, Nishishita S, Ye X, Konrad A. Comparison of The Effect of High- and Low- Frequency Vibration Foam Rolling on The Quadriceps Muscle. J Sports Sci Med. 2022 Sep 1;21(3):376-382. doi: 10.52082/jssm.2022.376. PMID: 36157391; PMCID:  PMC9459764. | Excluded：review |
| 248 | Foucher KC, Chmell SJ, Courtney CA. Duration of symptoms is associated with conditioned pain modulation and somatosensory measures in knee osteoarthritis. J Orthop Res. 2019 Jan;37(1):136-142. doi: 10.1002/jor.24159. Epub 2018 Nov 1. PMID: 30325066. | Excluded：review |
| 249 | Yang F, Underdahl M, Yang H, Yang C. Effects of vibration intensity on lower limb joint moments during standing. J Biomech. 2019 May 9;88:18-24. doi: 10.1016/j.jbiomech.2019.03.012. Epub 2019 Mar 18. PMID: 30904333. | Excluded：review |
| 250 | Andersen RE, Arendt-Nielsen L, Madeleine P. Knee joint vibroarthrography of asymptomatic subjects during loaded flexion-extension movements. Med Biol Eng Comput. 2018 Dec;56(12):2301-2312. doi: 10.1007/s11517-018-1856-6. Epub 2018 Jun 21. PMID: 29926251. | Excluded：review |
| 251 | Xia H, Charlton JM, Shull PB, Hunt MA. Portable, automated foot progression angle gait modification via a proof-of-concept haptic feedback-sensorized shoe. J Biomech. 2020 Jun 23;107:109789. doi: 10.1016/j.jbiomech.2020.109789. Epub 2020 Apr 13. PMID: 32321637. | Excluded：review |
| 252 | Qu X, Jiang J, Hu X. Effects of Subsensory Noise and Fatigue on Knee Landing and Cross-over Cutting Biomechanics in Male Athletes. J Appl Biomech. 2018 Jun 1;34(3):205-210. doi: 10.1123/jab.2017-0180. Epub 2018 May 25. PMID: 29364038. | Excluded：review |
| 253 | Tsai ST, Li CF, Chi KC, Ko LW, Stevenson C, Chen YJ, Chen CH. Immediate Effect of Whole Body Vibration on Knee Extensor Tendon Stiffness in Hemiparetic Stroke Patients. Medicina (Kaunas). 2021 Sep 29;57(10):1037. doi: 10.3390/medicina57101037. PMID: 34684074; PMCID: PMC8540205. | Excluded：review |
| 254 | Nagao M, Konno S, Kim YH, Yokota O. Frequency response in bone joint acoustic sensor development. Technol Health Care. 2015;23(6):715-27. doi: 10.3233/THC-151024. PMID: 26409512. | Excluded：review |
| 255 | Kręcisz K, Bączkowicz D. Analysis and multiclass classification of pathological knee joints using vibroarthrographic signals. Comput Methods Programs Biomed. 2018 Feb;154:37-44. doi: 10.1016/j.cmpb.2017.10.027. Epub 2017 Nov 8. PMID: 29249345. | Excluded：review |
| 256 | Pollock RD, Woledge RC, Mills KR, Martin FC, Newham DJ. Muscle activity and acceleration during whole body vibration: effect of frequency and amplitude.Clin Biomech (Bristol, Avon). 2010 Oct;25(8):840-6. doi: 10.1016/j.clinbiomech.2010.05.004. Epub 2010 Jun 11. PMID: 20541297. | Excluded：review |
| 257 | Taleshi N, Brownjohn JMW, Lamb SE, Zivanovic S, Williams GKR. Vector coding reveals the underlying balance control strategies used by humans during  translational perturbation. Sci Rep. 2022 Dec 5;12(1):21030. doi: 10.1038/s41598-022-24731-3. PMID: 36470936; PMCID: PMC9722668. | Excluded：review |
| 258 | Nakamura M, Kasahara K, Yoshida R, Yahata K, Sato S, Murakami Y, Aizawa K, Konrad A. Cross-education effect of vibration foam rolling on eccentrically  damaged muscles. J Musculoskelet Neuronal Interact. 2022 Sep 1;22(3):369-374.  PMID: 36046993; PMCID: PMC9438511. | Excluded：review |
| 259 | aryn RC, Hazell TJ, Dickey JP. Transmission of acceleration from a synchronous vibration exercise platform to the head. Int J Sports Med. 2014 Apr;35(4):330-8. doi: 10.1055/s-0033-1349105. Epub 2013 Sep 30. PMID: 24081617. | Excluded：review |
| 260 | Deng L, Yang Y, Yang C, Fang Y, Zhang X, Liu L, Fu W. Compression Garments Reduce Soft Tissue Vibrations and Muscle Activations during Drop Jumps: An Accelerometry Evaluation. Sensors (Basel). 2021 Aug 21;21(16):5644. doi: 10.3390/s21165644. PMID: 34451085; PMCID: PMC8402353. | Excluded：review |
| 261 | Karaca Umay E, Gurcay E, Karsli PB, Cakci A. Sensory disturbance and polyneuropathy in rheumatoid arthritis patients with foot deformity. Rev Bras Reumatol Engl Ed. 2016 May-Jun;56(3):191-7. English, Portuguese. doi: 10.1016/j.rbre.2015.08.010. Epub 2015 Sep 8. PMID: 27267636. | Excluded：review |
| 262 | Lindsay KG, Nichols DL, Davis RW, Marshall DD. The Effect of Whole-Body Vibration on Lower-Body Resistance Detraining in College-Age Women. Res Q Exerc Sport. 2018 Mar;89(1):57-65. doi: 10.1080/02701367.2017.1401210. Epub 2017 Dec 8. PMID: 29220619. | Excluded：review |
| 263 | Blackburn J, Wylde V, Greenwood R, Blom AW, Levy A. The effect of numbness on outcome from total knee replacement. Ann R Coll Surg Engl. 2017 May;99(5):385-389. doi: 10.1308/rcsann.2017.0026. PMID: 28462655; PMCID: PMC5449701. | Excluded：review |
| 264 | Ma C, Yang J, Wang Q, Liu H, Xu H, Ding T, Yang J. A method of feature fusion and dimension reduction for knee joint pathology screening and separability evaluation criteria. Comput Methods Programs Biomed. 2022 Sep;224:106992. doi: 10.1016/j.cmpb.2022.106992. Epub 2022 Jun 30. PMID:35810509. | Excluded：review |
| 265 | Tankisheva E, Jonkers I, Boonen S, Delecluse C, van Lenthe GH, Druyts HL, Spaepen P, Verschueren SM. Transmission of whole-body vibration and its effect on muscle activation. J Strength Cond Res. 2013 Sep;27(9):2533-41. doi:10.1519/JSC.0b013e31827f1225. PMID: 23222086. | Excluded：review |
| 266 | Schleer P, Kaiser P, Drobinsky S, Radermacher K. Augmentation of haptic feedback for teleoperated robotic surgery. Int J Comput Assist Radiol Surg. 2020 Mar;15(3):515-529. doi: 10.1007/s11548-020-02118-x. Epub 2020 Jan 30. PMID: 32002750; PMCID: PMC7036061. | Excluded：review |
| 267 | Lowe T, Dong XN, Griffin L. Hamstrings vibration reduces tibiofemoral compressive force following anterior cruciate ligament reconstruction. J Orthop Res. 2024 Apr;42(4):788-797. doi: 10.1002/jor.25736. Epub 2023 Nov 27. PMID:37975273. | Excluded：review |
| 268 | Blackburn JT, Pamukoff DN, Sakr M, Vaughan AJ, Berkoff DJ. Whole body and local muscle vibration reduce artificially induced quadriceps arthrogenic inhibition. Arch Phys Med Rehabil. 2014 Nov;95(11):2021-8. doi: 10.1016/j.apmr.2014.07.393. Epub 2014 Jul 30. PMID: 25083559. | Excluded：review |
| 269 | Patel M, Nilsson MH, Rehncrona S, Tjernström F, Magnusson M, Johansson R, Fransson PA. Strategic alterations of posture are delayed in Parkinson's disease patients during deep brain stimulation. Sci Rep. 2021 Dec 7;11(1):23550. doi: 10.1038/s41598-021-02813-y. PMID: 34876604; PMCID: PMC8651728. | Excluded：review |
| 270 | Krause A, Gollhofer A, Lee K, Freyler K, Becker T, Kurz A, Ritzmann R. Acute whole-body vibration reduces post-activation depression in the triceps surae muscle. Hum Mov Sci. 2020 Aug;72:102655. doi: 10.1016/j.humov.2020.102655. Epub 2020 Jul 9. PMID: 32721374. | Excluded：review |
| 271 | Yang Z, Miller T, Xiang Z, Pang MYC. Effects of different vibration frequencies on muscle strength, bone turnover and walking endurance in chronic stroke. Sci Rep. 2021 Jan 8;11(1):121. doi: 10.1038/s41598-020-80526-4. PMID:  33420277; PMCID: PMC7794423. | Excluded：review |
| 272 | Dretakis K, Koutserimpas C. Pitfalls with the MAKO Robotic-Arm-Assisted Total Knee Arthroplasty. Medicina (Kaunas). 2024 Feb 2;60(2):262. doi: 10.3390/medicina60020262. PMID: 38399549; PMCID: PMC10890000. | Excluded：review |
| 273 | Ozmen GC, Safaei M, Semiz B, Whittingslow DC, Hunnicutt JL, Prahalad S, Hash R, Xerogeanes JW, Inan OT. Detection of Meniscal Tear Effects on Tibial Vibration Using Passive Knee Sound Measurements. IEEE Trans Biomed Eng. 2021 Jul;68(7):2241-2250. doi: 10.1109/TBME.2020.3048930. Epub 2021 Jun 17. PMID:  33400643; PMCID: PMC8284919. | Excluded：review |
| 274 | Priya L, Vignesh V, Krishnan V, Ajeesh RP. Design and development of a smart knee pain relief pad based on vibration and alternate heating and cooling treatments. Technol Health Care. 2018;26(3):543-551. doi: 10.3233/THC-181213. PMID: 29630572. | Excluded：review |
| 275 | Trama R, Blache Y, Hintzy F, Rossi J, Millet GY, Hautier C. Does neuromuscular fatigue generated by trail running modify foot-ground impact and soft tissue vibrations? Eur J Sport Sci. 2023 Jul;23(7):1155-1163. doi: 10.1080/17461391.2022.2093649. Epub 2022 Jul 10. PMID: 35730761. | Excluded：review |
| 276 | Kręcisz K, Bączkowicz D, Kawala-Sterniuk A. Using Nonlinear Vibroartrographic Parameters for Age-Related Changes Assessment in Knee Arthrokinematics. Sensors (Basel). 2022 Jul 25;22(15):5549. doi: 10.3390/s22155549. PMID: 35898052; PMCID: PMC9370942. | Excluded：review |
| 277 | Hackshaw KV, Aykas DP, Sigurdson GT, Plans M, Madiai F, Yu L, Buffington CAT, Giusti MM, Rodriguez-Saona L. Metabolic fingerprinting for diagnosis of fibromyalgia and other rheumatologic disorders. J Biol Chem. 2019 Feb  15;294(7):2555-2568. doi: 10.1074/jbc.RA118.005816. Epub 2018 Dec 6. Erratum in: J Biol Chem. 2020 Apr 24;295(17):5834. doi: 10.1074/jbc.AAC120.013667. PMID: 30523152; PMCID: PMC6378985. | Excluded：review |
| 278 | Barrera-Curiel A, Colquhoun RJ, Hernandez-Sarabia JA, DeFreitas JM. The effects of vibration-induced altered stretch reflex sensitivity on maximal motor unit firing properties. J Neurophysiol. 2019 Jun 1;121(6):2215-2221. doi:10.1152/jn.00326.2018. Epub 2019 Apr 10. PMID: 30969899. | Excluded：review |
| 279 | Maffiuletti NA, Saugy J, Cardinale M, Micallef JP, Place N. Neuromuscular fatigue induced by whole-body vibration exercise. Eur J Appl Physiol. 2013 Jun;113(6):1625-34. doi: 10.1007/s00421-013-2590-2. Epub 2013 Jan 24. PMID:23344670. | Excluded：review |
| 280 | Souron R, Zambelli A, Espeit L, Besson T, Cochrane DJ, Lapole T. Active versus local vibration warm-up effects on knee extensors stiffness and neuromuscular performance of healthy young males. J Sci Med Sport. 2019 Feb;22(2):206-211. doi: 10.1016/j.jsams.2018.07.003. Epub 2018 Jul 10. PMID:30017464. | Excluded：review |
| 281 | Schuster E, Routson RL, Hinchcliff M, Benoff K, Suri P, Richburg C, Muir BC, Czerniecki JM, Aubin PM. A novel walking cane with haptic biofeedback reduces knee adduction moment in the osteoarthritic knee. J Biomech. 2021 Jan 4;114:110150. doi: 10.1016/j.jbiomech.2020.110150. Epub 2020 Nov 28. PMID:33285489; PMCID: PMC9027918. | Excluded：review |
| 282 | Chang CM, Tsai CH, Lu MK, Tseng HC, Lu G, Liu BL, Lin HC. The neuromuscular responses in patients with Parkinson's disease under different conditions during whole-body vibration training. BMC Complement Med Ther. 2022 Jan 3;22(1):2. doi:10.1186/s12906-021-03481-1. PMID: 34980075; PMCID: PMC8722001. | Excluded：review |
| 283 | Cochrane DJ, Stannard SR, Firth EC, Rittweger J. Comparing muscle temperature during static and dynamic squatting with and without whole-body vibration. Clin Physiol Funct Imaging. 2010 Jul;30(4):223-9. doi: 10.1111/j.1475-097X.2010.00931.x. Epub 2010 May 11. PMID: 20491843. | Excluded：review |
| 284 | Xia H, Xu J, Wang J, Hunt MA, Shull PB. Validation of a smart shoe for estimating foot progression angle during walking gait. J Biomech. 2017 Aug 16;61:193-198. doi: 10.1016/j.jbiomech.2017.07.012. Epub 2017 Jul 25. PMID:  28780187. | Excluded：review |
| 285 | Nakamura M, Kasahara K, Yoshida R, Yahata K, Sato S, Murakami Y, Aizawa K, Konrad A. The Effect of Static Compression via Vibration Foam Rolling on Eccentrically Damaged Muscle. Int J Environ Res Public Health. 2022 Feb 5;19(3):1823. doi: 10.3390/ijerph19031823. PMID: 35162844; PMCID: PMC8834946. | Excluded：review |
| 286 | Thompson C, Bélanger M, Fung J. Effects of plantar cutaneo-muscular and tendon vibration on posture and balance during quiet and perturbed stance. Hum Mov Sci. 2011 Apr;30(2):153-71. doi: 10.1016/j.humov.2010.04.002. Epub 2010 Jun 26. PMID: 20580112. | Excluded：review |
| 287 | Huang M, Tang CY, Pang MYC. Use of whole body vibration in individuals with chronic stroke: Transmissibility and signal purity. J Biomech. 2018 May  17;73:80-91. doi: 10.1016/j.jbiomech.2018.03.022. Epub 2018 Mar 17. PMID:29588022. | Excluded：review |
| 288 | Yu S, Lowe T, Griffin L, Dong XN. Single bout of vibration-induced hamstrings fatigue reduces quadriceps inhibition and coactivation of knee muscles after anterior cruciate ligament (ACL) reconstruction. J Electromyogr  Kinesiol. 2020 Dec;55:102464. doi: 10.1016/j.jelekin.2020.102464. Epub 2020 Sep 9. PMID: 32942109. | Excluded：review |
| 289 | Petit PD, Pensini M, Tessaro J, Desnuelle C, Legros P, Colson SS. Optimal whole-body vibration settings for muscle strength and power enhancement in human knee extensors. J Electromyogr Kinesiol. 2010 Dec;20(6):1186-95. doi: 10.1016/j.jelekin.2010.08.002. PMID: 20801671. | Excluded：review |
| 290 | Yang F, Munoz J, Han LZ, Yang F. Effects of vibration training in reducing risk of slip-related falls among young adults with obesity. J Biomech. 2017 May 24;57:87-93. doi: 10.1016/j.jbiomech.2017.03.024. Epub 2017 Apr 9. PMID:  28431747. | Excluded：review |
| 291 | Braillon A. Efficacy and safety of duloxetine in osteoarthritis or chronic low back pain? The tip of an iceberg! Osteoarthritis Cartilage. 2020 Sep;28(9):1298-1299. doi: 10.1016/j.joca.2020.04.019. Epub 2020 May 21. PMID: 32446939. | Excluded：review |
| 292 | De Nardi M, Facheris C, Ruggeri P, La Torre A, Codella R. High-impact Routines to Ameliorate Trunk and Lower Limbs Flexibility in Women. Int J Sports Med. 2020 Dec;41(14):1039-1046. doi: 10.1055/a-1119-7902. Epub 2020 Jul 15.PMID: 32668475. | Excluded：review |
| 293 | Troy Blackburn J, Dewig DR, Johnston CD. Time course of the effects of vibration on quadriceps function in individuals with anterior cruciate ligament reconstruction. J Electromyogr Kinesiol. 2021 Feb;56:102508. doi:10.1016/j.jelekin.2020.102508. Epub 2020 Nov 28. PMID: 33302006. | Excluded：review |
| 294 | Tanaka N, Hoshiyama M. Vibroarthrography in patients with knee arthropathy. J Back Musculoskelet Rehabil. 2012;25(2):117-22. doi: 10.3233/BMR-2012-0319. PMID: 22684203. | Excluded：review |
| 295 | Nawayseh N, Hamdan S. Power Absorbed by the Standing Human Body During Whole-Body Vibration Training. J Biomech Eng. 2020 Jul 1;142(7):074501. doi:10.1115/1.4045809. PMID: 31891372. | Excluded：review |
| 296 | Fisher J, Van-Dongen M, Sutherland R. Combined isometric and vibration training does not enhance strength beyond that of isometric training alone. J Sports Med Phys Fitness. 2015 Sep;55(9):899-904. Epub 2014 Jun 19. PMID: 24947812. | Excluded：review |
| 297 | Dumas G, Perrin P, Ouedraogo E, Schmerber S. How to perform the skull vibration-induced nystagmus test (SVINT). Eur Ann Otorhinolaryngol Head Neck Dis. 2016 Nov;133(5):343-348. doi: 10.1016/j.anorl.2016.04.002. Epub 2016 May 6.PMID: 27161530. | Excluded：review |
| 298 | Marín PJ, García Rioja J, Bernardo-Filho M, Hazell TJ. Effects of Different Magnitudes of Whole-Body Vibration on Dynamic Squatting Performance. J Strength Cond Res. 2015 Oct;29(10):2881-7. doi: 10.1519/JSC.0000000000000940. PMID:25807029. | Excluded：review |
| 299 | Brunetti O, Botti FM, Roscini M, Brunetti A, Panichi R, Filippi GM, Biscarini A, Pettorossi VE. Focal vibration of quadriceps muscle enhances leg power and decreases knee joint laxity in female volleyball players. J Sports Med Phys Fitness. 2012 Dec;52(6):596-605. PMID: 23187322. | Excluded：review |
| 300 | Nagai T, Bates NA, Hewett TE, Schilaty ND. Effects of localized vibration on knee joint position sense in individuals with anterior cruciate ligament reconstruction. Clin Biomech (Bristol, Avon). 2018 Jun;55:40-44. doi:  10.1016/j.clinbiomech.2018.04.011. Epub 2018 Apr 14. PMID: 29680779; PMCID: PMC5960437. | Excluded：review |
| 301 | Lee CL, Chu IH, Lyu BJ, Chang WD, Chang NJ. Comparison of vibration rolling, nonvibration rolling, and static stretching as a warm-up exercise on flexibility, joint proprioception, muscle strength, and balance in young adults. J Sports Sci. 2018 Nov;36(22):2575-2582. doi: 10.1080/02640414.2018.1469848. Epub 2018 Apr 26. PMID: 29697023. | Excluded：review |
| 302 | Jakorinne P, Haanpää M, Arokoski J. Reliability of pressure pain, vibration detection, and tactile detection threshold measurements in lower extremities in subjects with knee osteoarthritis and healthy controls. Scand J Rheumatol. 2018 Nov;47(6):491-500. doi: 10.1080/03009742.2018.1433233. Epub 2018 Jun 25. PMID:29939097. | Excluded：review |
| 303 | Spiliopoulou SI, Amiridis IG, Tsigganos G, Economides D, Kellis E.Vibration effects on static balance and strength. Int J Sports Med. 2010 Sep;31(9):610-6. doi: 10.1055/s-0030-1249618. Epub 2010 Jun 29. PMID: 20589590. | Excluded：review |
| 304 | Pujol J, Ramos-López D, Blanco-Hinojo L, Pujol G, Ortiz H, Martínez-Vilavella G, Blanch J, Monfort J, Deus J. Testing the effects of gentle vibrotactile stimulation on symptom relief in fibromyalgia. Arthritis Res Ther. 2019 Jun 14;21(1):148. doi: 10.1186/s13075-019-1932-9. PMID: 31200775; PMCID:  PMC6570892. | Excluded：review |
| 305 | Harnie J, Cattagni T, Cornu C, McNair P, Jubeau M. Acute effect of tendon vibration applied during isometric contraction at two knee angles on maximal knee extension force production. PLoS One. 2020 Nov 13;15(11):e0242324. doi:10.1371/journal.pone.0242324. PMID: 33186411; PMCID: PMC7665630. | Excluded：review |
| 306 | Fischer AG, Erhart-Hledik JC, Asay JL, Andriacchi TP. Intermittent vibrational stimulation enhances mobility during stair navigation in patients with knee pain. Gait Posture. 2021 May;86:125-131. doi: 10.1016/j.gaitpost.2021.03.013. Epub 2021 Mar 8. PMID: 33721689. | Excluded：review |
| 307 | Rome K, Erikson K, Otene C, Sahid H, Sangster K, Gow P. Clinical characteristics of foot ulceration in people with chronic gout. Int Wound J. 2016 Apr;13(2):209-15. doi: 10.1111/iwj.12262. Epub 2014 Mar 28. PMID: 24674139;  PMCID: PMC7949590. | Excluded：review |
| 308 | Maupas E, Dyer JO, Melo SA, Forget R. Patellar tendon vibration reduces the increased facilitation from quadriceps to soleus in post-stroke hemiparetic individuals. Ann Phys Rehabil Med. 2017 Sep;60(5):319-328. doi: 10.1016/j.rehab.2017.03.008. Epub 2017 May 18. PMID: 28528818. | Excluded：review |
| 309 | Mason KJ, O'Neill TW, Lunt M, Jones AKP, McBeth J. Psychosocial factors partially mediate the relationship between mechanical hyperalgesia and self-reported pain. Scand J Pain. 2018 Jan 26;18(1):59-69. doi:10.1515/sjpain-2017-0109. PMID: 29794289. | Excluded：review |
| 310 | Pamukoff DN, Pietrosimone B, Ryan ED, Lee DR, Brown LE, Blackburn JT. Whole-Body Vibration Improves Early Rate of Torque Development in Individuals With Anterior Cruciate Ligament Reconstruction. J Strength Cond Res. 2017 Nov;31(11):2992-3000. doi: 10.1519/JSC.0000000000001740. PMID: 27930453. | Excluded：review |
| 311 | Da Silva-Grigoletto ME, De Hoyo M, Sañudo B, Carrasco L, García-Manso JM. Determining the optimal whole-body vibration dose-response relationship for muscle performance. J Strength Cond Res. 2011 Dec;25(12):3326-33. doi: 10.1519/JSC.0b013e3182163047. PMID: 22080316. | Excluded：review |
| 312 | Coelho-Oliveira AC, Lacerda ACR, de Souza ALC, Santos LMM, da Fonseca SF, Dos Santos JM, Ribeiro VGC, Leite HR, Figueiredo PHS, Fernandes JSC, Martins F, Filho RGT, Bernardo-Filho M, da Cunha de Sá-Caputo D, Sartorio A, Cochrane D, Lima VP, Costa HS, Mendonça VA, Taiar R. Acute Whole-Body Vibration Exercise Promotes Favorable Handgrip Neuromuscular Modifications in Rheumatoid Arthritis: A Cross-Over Randomized Clinical. Biomed Res Int. 2021 Dec 2;2021:9774980. doi: 10.1155/2021/9774980. PMID: 34901282; PMCID: PMC8660187. | Excluded：review |
| 313 | Feland JB, Hawks M, Hopkins JT, Hunter I, Johnson AW, Eggett DL. Whole body vibration as an adjunct to static stretching. Int J Sports Med. 2010 Aug;31(8):584-9. doi: 10.1055/s-0030-1254084. Epub 2010 Jun 9. PMID: 20535662. | Excluded：review |
| 314 | Kasahara K, Yoshida R, Yahata K, Sato S, Murakami Y, Aizawa K, Konrad A, Nakamura M. Comparison of the Acute Effects of Foam Rolling with High and Low Vibration Frequencies on Eccentrically Damaged Muscle. J Sports Sci Med. 2022 Feb 15;21(1):112-119. doi: 10.52082/jssm.2022.112. PMID: 35250340; PMCID: PMC8851125. | Excluded：review |
| 315 | Pamukoff DN, Pietrosimone B, Lewek MD, Ryan ED, Weinhold PS, Lee DR, Blackburn JT. Whole-Body and Local Muscle Vibration Immediately Improve Quadriceps Function in Individuals With Anterior Cruciate Ligament  Reconstruction. Arch Phys Med Rehabil. 2016 Jul;97(7):1121-9. doi: 10.1016/j.apmr.2016.01.021. Epub 2016 Feb 8. PMID: 26869286. | Excluded：review |
| 316 | Jiménez-Fabián R, Verlinden O. Review of control algorithms for robotic ankle systems in lower-limb orthoses, prostheses, and exoskeletons. Med Eng Phys. 2012 May;34(4):397-408. doi: 10.1016/j.medengphy.2011.11.018. Epub 2011 Dec 15. PMID: 22177895. | Excluded：review |
| 317 | Nawayseh N, Hamdan S. Apparent mass of the standing human body when using a whole-body vibration training machine: Effect of knee angle and input frequency.  J Biomech. 2019 Jan 3;82:291-298. doi: 10.1016/j.jbiomech.2018.11.003. Epub 2018  Nov 13. PMID: 30466950. | Excluded：review |
| 318 | Mahmoudian A, van Dieen JH, Baert IA, Jonkers I, Bruijn SM, Luyten FP, Faber GS, Verschueren SM. Changes in proprioceptive weighting during quiet standing in women with early and established knee osteoarthritis compared to  healthy controls. Gait Posture. 2016 Feb;44:184-8. doi:  10.1016/j.gaitpost.2015.12.010. Epub 2015 Dec 14. PMID: 27004655. | Excluded：review |
| 319 | Spiliopoulou SI, Amiridis IG, Tsigganos G, Hatzitaki V. Side-alternating vibration training for balance and ankle muscle strength in untrained women. J Athl Train. 2013 Sep-Oct;48(5):590-600. doi: 10.4085/1062-6050-48.4.03. Epub 2013 Aug 5. PMID: 23914911; PMCID: PMC3784360. | Excluded：review |
| 320 | Selionov VA, Solopova IA, Zhvansky DS, Karabanov AV, Chernikova LA, Gurfinkel VS, Ivanenko YP. Lack of non-voluntary stepping responses in Parkinson's disease. Neuroscience. 2013 Apr 3;235:96-108. doi: 10.1016/j.neuroscience.2012.12.064. Epub 2013 Jan 12. PMID: 23321538. | Excluded：review |
| 321 | Casal-Beiroa P, González P, Blanco FJ, Magalhães J. Molecular analysis of the destruction of articular joint tissues by Raman spectroscopy. Expert Rev Mol Diagn. 2020 Aug;20(8):789-802. doi: 10.1080/14737159.2020.1782747. Epub 2020 Jun 30. PMID: 32538250. | Excluded：review |
| 322 | Bączkowicz D, Majorczyk E, Kręcisz K. Age-related impairment of quality of joint motion in vibroarthrographic signal analysis. Biomed Res Int. 2015;2015:591707. doi: 10.1155/2015/591707. Epub 2015 Feb 23. PMID: 25802856; PMCID: PMC4352744. | Excluded：review |
| 323 | Shakoor N, Lee KJ, Fogg LF, Wimmer MA, Foucher KC, Mikolaitis RA, Block JA. The relationship of vibratory perception to dynamic joint loading, radiographic severity, and pain in knee osteoarthritis. Arthritis Rheum. 2012 Jan;64(1):181-6. doi: 10.1002/art.30657. PMID: 21898358. | Excluded：review |
| 324 | Kavchak AJ, Fernández-de-Las-Peñas C, Rubin LH, Arendt-Nielsen L, Chmell SJ, Durr RK, Courtney CA. Association between altered somatosensation, pain, and  knee stability in patients with severe knee osteoarthrosis. Clin J Pain. 2012 Sep;28(7):589-94. doi: 10.1097/AJP.0b013e31823ae18f. PMID: 22146110. | Excluded：review |
| 325 | Kędzierska K, Synder M, Kozłowski P. Influence of Vibroacoustic Therapy on Local Status and Rehabilitation of Post-TKR and Post-THR patients. Ortop Traumatol Rehabil. 2021 Apr 30;23(2):101-113. doi: 10.5604/01.3001.0014.8139.PMID: 33958495. | Excluded：review |
| 326 | Sañudo B, Feria A, Carrasco L, de Hoyo M, Santos R, Gamboa H. Gender differences in knee stability in response to whole-body vibration. J Strength Cond Res. 2012 Aug;26(8):2156-65. doi: 10.1519/JSC.0b013e31823b0716. PMID: 21997457. | Excluded：review |
| 327 | Marín-Cascales E, Rubio-Arias JA, Romero-Arenas S, Alcaraz PE. Effect of 12 Weeks of Whole-Body Vibration Versus Multi-Component Training in Post-Menopausal Women. Rejuvenation Res. 2015 Dec;18(6):508-16. doi: 10.1089/rej.2015.1681. Epub 2015 Sep 29. PMID: 25978688. | Excluded：review |
| 328 | Konishi Y, McNair PJ, Rice DA. TENS Alleviates Muscle Weakness Attributable to Attenuation of Ia Afferents. Int J Sports Med. 2017 Mar;38(3):253-257. doi: 10.1055/s-0042-118183. Epub 2017 Feb 13. PMID: 28192829. | Excluded：review |
| 329 | Goetschius J, Hertel J, Saliba S, Brockmeier SF, Hart JM. The effects of patellar tendon vibration on quadriceps strength in anterior cruciate ligament reconstructed knees. Phys Ther Sport. 2019 Nov;40:71-77. doi: 10.1016/j.ptsp.2019.08.014. Epub 2019 Sep 4. PMID: 31499398. | Excluded：review |
| 330 | Madeleine P, Andersen RE, Larsen JB, Arendt-Nielsen L, Samani A. Wireless multichannel vibroarthrographic recordings for the assessment of knee osteoarthritis during three activities of daily living. Clin Biomech (Bristol, Avon). 2020 Feb;72:16-23. doi: 10.1016/j.clinbiomech.2019.11.015. Epub 2019 Nov 26. PMID: 31794924. | Excluded：review |
| 331 | Onushko T, Hyngstrom A, Schmit BD. Hip proprioceptors preferentially modulate reflexes of the leg in human spinal cord injury. J Neurophysiol. 2013 Jul;110(2):297-306. doi: 10.1152/jn.00261.2012. Epub 2013 Apr 24. PMID: 23615544; PMCID: PMC4073919. | Excluded：review |
| 332 | Carrasco VB, Vidal JM, Caparrós-Manosalva C. Vibration motor stimulation device in smart leggings that promotes motor performance in older people. Med Biol Eng Comput. 2023 Mar;61(3):635-649. doi: 10.1007/s11517-022-02733-7. Epub 2022 Dec 27. PMID: 36574174. | Excluded：review |
| 333 | Jemni M, Mkaouer B, Marina M, Asllani A, Sands WA. Acute static vibration- induced stretching enhanced muscle viscoelasticity but did not affect maximal voluntary contractions in footballers. J Strength Cond Res. 2014  Nov;28(11):3105-14. doi: 10.1519/JSC.0000000000000404. PMID: 25051000. | Excluded：review |
| 334 | Fagnani F, Giombini A, Di Cesare A, Pigozzi F, Di Salvo V. The effects of a whole-body vibration program on muscle performance and flexibility in female athletes. Am J Phys Med Rehabil. 2006 Dec;85(12):956-62. doi: 10.1097/01.phm.0000247652.94486.92. PMID: 17117001. | Excluded：review |
| 335 | Avelar NC, Ribeiro VG, Mezêncio B, Fonseca SF, Tossige-Gomes R, da Costa SJ, Szmuchrowski L, Gripp F, Coimbra CC, Lacerda AC. Influence of the knee flexion on muscle activation and transmissibility during whole body vibration. J Electromyogr Kinesiol. 2013 Aug;23(4):844-50. doi: 10.1016/j.jelekin.2013.03.014. Epub 2013 May 3. PMID: 23643467. | Excluded：review |
| 336 | van Eeden FM, van Halewijn KF, Swart NM, Festen DA. Efficacy and Safety of Leg-Press Training With Moderate Vibration After Total Knee Arthroplasty Remains Unclear. Arch Phys Med Rehabil. 2016 Nov;97(11):2018. doi: 10.1016/j.apmr.2016.06.025. PMID: 27780526. | Excluded：review |
| 337 | Cronin J, Nash M, Whatman C. The acute effects of hamstring stretching and vibration on dynamic knee joint range of motion and jump performance. Phys Ther Sport. 2008 May;9(2):89-96. doi: 10.1016/j.ptsp.2008.01.003. Epub 2008 Apr 3. PMID: 19083708. | Excluded：review |
| 338 | Shull PB, Huang Y, Schlotman T, Reinbolt JA. Muscle force modification strategies are not consistent for gait retraining to reduce the knee adduction moment in individuals with knee osteoarthritis. J Biomech. 2015 Sep  18;48(12):3163-9. doi: 10.1016/j.jbiomech.2015.07.006. Epub 2015 Jul 17. PMID: 26209875. | Excluded：review |
| 339 | Simsek D. Different fatigue-resistant leg muscles and EMG response during whole-body vibration. J Electromyogr Kinesiol. 2017 Dec;37:147-154. doi: 10.1016/j.jelekin.2017.10.006. Epub 2017 Oct 19. PMID: 29102877. | Excluded：review |
| 340 | Simão AP, Avelar NC, Tossige-Gomes R, Neves CD, Mendonça VA, Miranda AS, Teixeira MM, Teixeira AL, Andrade AP, Coimbra CC, Lacerda AC. Functional performance and inflammatory cytokines after squat exercises and whole-body vibration in elderly individuals with knee osteoarthritis. Arch Phys Med Rehabil. 2012 Oct;93(10):1692-700. doi: 10.1016/j.apmr.2012.04.017. Epub 2012 Apr 27. PMID: 22546535. | Excluded：review |
| 341 | Edionwe J, Hess C, Fernandez-Rio J, Herndon DN, Andersen CR, Klein GL, Suman OE, Amonette WE. Effects of whole-body vibration exercise on bone mineral content and density in thermally injured children. Burns. 2016 May;42(3):605-13. doi: 10.1016/j.burns.2015.10.017. Epub 2016 Jan 18. PMID: 26796240; PMCID: PMC4880497. | Excluded：review |
| 326 | Wunderer K, Schabrun SM, Chipchase LS. Effects of whole body vibration on strength and functional mobility in multiple sclerosis. Physiother Theory Pract. 2010 Aug;26(6):374-84. doi: 10.3109/09593980903147384. PMID: 20658923. | Excluded：review |
| 327 | Chen ZR, Peng HT, Siao SW, Hou YT, Wang LI. Whole Body Vibration Immediately Decreases Lower Extremity Loading During the Drop Jump. J Strength Cond Res. 2016 Sep;30(9):2476-81. doi: 10.1519/JSC.0000000000001358. PMID: 26849793. | Excluded：review |
| 328 | Guggenheimer JD, Dickin DC, Reyes GF, Dolny DG. The effects of specific preconditioning activities on acute sprint performance. J Strength Cond Res. 2009 Jul;23(4):1135-9. doi: 10.1519/JSC.0b013e318191892e. PMID: 19528862. | Excluded：review |
| 329 | Trans T, Aaboe J, Henriksen M, Christensen R, Bliddal H, Lund H. Effect of whole body vibration exercise on muscle strength and proprioception in females with knee osteoarthritis. Knee. 2009 Aug;16(4):256-61. doi: 10.1016/j.knee.2008.11.014. Epub 2009 Jan 15. PMID: 19147365. | Excluded：review |
| 330 | Apinyankul R, Siriwattanasit K, Srungboonmee K, Witayakom W, Kosuwon W. A vibration sensor approach to detect intra-articular needle tip placement in the knee joint: a proof-of-concept study. BMC Musculoskelet Disord. 2021 Nov 15;22(1):946. doi: 10.1186/s12891-021-04836-y. PMID: 34781957; PMCID:PMC8591594. | Excluded：review |
| 331 | Raimundo AM, Gusi N, Tomas-Carus P. Fitness efficacy of vibratory exercise compared to walking in postmenopausal women. Eur J Appl Physiol. 2009 Jul;106(5):741-8. doi: 10.1007/s00421-009-1067-9. Epub 2009 May 12. PMID:  19434420. | Excluded：review |
| 332 | Gharehbaghi S, Whittingslow DC, Ponder LA, Prahalad S, Inan OT. Acoustic Emissions From Loaded and Unloaded Knees to Assess Joint Health in Patients With Juvenile Idiopathic Arthritis. IEEE J Biomed Health Inform. 2021  Sep;25(9):3618-3626. doi: 10.1109/JBHI.2021.3081429. Epub 2021 Sep 3. PMID: 34003759. | Excluded：review |
| 333 | Ritzmann R, Gollhofer A, Kramer A. The influence of vibration type, frequency, body position and additional load on the neuromuscular activity during whole body vibration. Eur J Appl Physiol. 2013 Jan;113(1):1-11. doi: 10.1007/s00421-012-2402-0. Epub 2012 Apr 27. PMID: 22538279. | Excluded：review |
| 334 | Osawa Y, Oguma Y. Effects of resistance training with whole-body vibration on muscle fitness in untrained adults. Scand J Med Sci Sports. 2013 Feb;23(1):84-95. doi: 10.1111/j.1600-0838.2011.01352.x. Epub 2011 Aug 3. PMID:21812821. | Excluded：review |
| 335 | Minetto MA, Botter A, Gamerro G, Varvello I, Massazza G, Bellomo RG, Maffiuletti NA, Saggini R. Contralateral effect of short-duration unilateral neuromuscular electrical stimulation and focal vibration in healthy subjects. Eur J Phys Rehabil Med. 2018 Dec;54(6):911-920. doi: 10.23736/S1973-9087.18.05004-9. Epub 2018 Mar 12. PMID: 29532649. | Excluded：review |
| 336 | Ema R, Takayama H, Miyamoto N, Akagi R. Effect of prolonged vibration to synergistic and antagonistic muscles on the rectus femoris activation during multi-joint exercises. Eur J Appl Physiol. 2017 Oct;117(10):2109-2118. doi: 10.1007/s00421-017-3702-1. Epub 2017 Aug 28. PMID: 28849258; PMCID: PMC5594059. | Excluded：review |
| 337 | Shakoor N, Lee KJ, Fogg LF, Block JA. Generalized vibratory deficits in osteoarthritis of the hip. Arthritis Rheum. 2008 Sep 15;59(9):1237-40. doi: 10.1002/art.24004. Erratum in: Arthritis Rheum. 2009 Jan 15;61(1):142. PMID: 18759259; PMCID: PMC3653291. | Excluded：review |
| 338 | Lau RW, Yip SP, Pang MY. Whole-body vibration has no effect on neuromotor function and falls in chronic stroke. Med Sci Sports Exerc. 2012 Aug;44(8):1409-18. doi: 10.1249/MSS.0b013e31824e4f8c. PMID: 22330025. | Excluded：review |
| 339 | Munera M, Bertucci W, Duc S, Chiementin X. Transmission of whole body vibration to the lower body in static and dynamic half-squat exercises. Sports Biomech. 2016 Nov;15(4):409-28. doi: 10.1080/14763141.2016.1171894. Epub 2016 May 30. PMID: 27238625. | Excluded：review |
| 340 | Colson SS, Pensini M, Espinosa J, Garrandes F, Legros P. Whole-body vibration training effects on the physical performance of basketball players. J Strength Cond Res. 2010 Apr;24(4):999-1006. doi: 10.1519/JSC.0b013e3181c7bf10. PMID: 20300027. | Excluded：review |
| 341 | Rice DA, McNair PJ, Lewis GN. Mechanisms of quadriceps muscle weakness in knee joint osteoarthritis: the effects of prolonged vibration on torque and muscle activation in osteoarthritic and healthy control subjects. Arthritis Res Ther. 2011;13(5):R151. doi: 10.1186/ar3467. Epub 2011 Sep 20. PMID: 21933392; PMCID: PMC3308081. | Excluded：review |
| 342 | Calder KM, Martin A, Lydiate J, MacDermid JC, Galea V, MacIntyre NJ. Sensory nerve action potentials and sensory perception in women with arthritis of the hand. J Neuroeng Rehabil. 2012 May 10;9:27. doi: 10.1186/1743-0003-9-27.  PMID: 22575001; PMCID: PMC3480934. |  |
| 343 | Hirjaková Z, Šingliarová H, Bzdúšková D, Kimijanová J, Bučková K, Valkovič P, Hlavačka F. Postural stability and responses to vibrations in patients after anterior cruciate ligament surgical reconstruction. Physiol Res. 2016 Oct  24;65(Suppl 3):S409-S416. doi: 10.33549/physiolres.933437. PMID: 27775426. | Excluded：review |
| 344 | Alizadeh-Meghrazi M, Masani K, Popovic MR, Craven BC. Whole-body vibration during passive standing in individuals with spinal cord injury: effects of plate choice, frequency, amplitude, and subject's posture on vibration propagation. PMR. 2012 Dec;4(12):963-75. doi: 10.1016/j.pmrj.2012.08.012. Epub 2012 Oct 24. PMID: 23102716. | Excluded：review |
| 345 | Carlucci F, Felici F, Piccinini A, Haxhi J, Sacchetti M. Individual Optimal Frequency in Whole-Body Vibration: Effect of Protocol, Joint Angle, and Fatiguing Exercise. J Strength Cond Res. 2016 Dec;30(12):3503-3511. doi:  10.1519/JSC.0b013e3182955e42. PMID: 23588483. | Excluded：review |
| 346 | Lindsey BW, Xu J, Chiasson D, Shull P, Cortes N. Feasibility of Wearable Haptic Biofeedback Training for Reducing the Knee Abduction Moment During Overground Walking. J Biomech Eng. 2021 Apr 1;143(4):044501. doi:  10.1115/1.4048082. PMID: 32793949. | Excluded：review |
| 347 | Dalton BH, Contento VS, Power GA. Residual force enhancement during submaximal and maximal effort contractions of the plantar flexors across knee angle. J Biomech. 2018 Sep 10;78:70-76. doi: 10.1016/j.jbiomech.2018.07.019. Epub 2018 Jul 17. PMID: 30037580. | Excluded：review |
| 348 | Prioreschi A, Tikly M, McVeigh JA. A three month controlled intervention of intermittent whole body vibration designed to improve functional ability and attenuate bone loss in patients with rheumatoid arthritis. BMC Musculoskelet Disord. 2014 Nov 29;15:403. doi: 10.1186/1471-2474-15-403. PMID: 25433517; PMCID: PMC4265489. | Excluded：review |
| 349 | Rees S, Murphy A, Watsford M. Effects of vibration exercise on muscle performance and mobility in an older population. J Aging Phys Act. 2007 Oct;15(4):367-81. doi: 10.1123/japa.15.4.367. PMID: 18048942. | Excluded：review |
| 350 | Courtney CA, Steffen AD, Fernández-de-Las-Peñas C, Kim J, Chmell SJ. Joint Mobilization Enhances Mechanisms of Conditioned Pain Modulation in Individuals With Osteoarthritis of the Knee. J Orthop Sports Phys Ther. 2016  Mar;46(3):168-76. doi: 10.2519/jospt.2016.6259. Epub 2016 Jan 1. Erratum in: J Orthop Sports Phys Ther. 2016 Apr;46(4):313. doi: 10.2519/jospt.2016.46.4.313.  PMID: 26721229. | Excluded：review |
| 351 | Jamison RN, Mei A, Edwards RR, Ross EL. Efficacy of Vibrating Gloves for Chronic Hand Pain due to Osteoarthritis. Pain Med. 2018 May 1;19(5):1044-1057.  doi: 10.1093/pm/pnx230. PMID: 29025096. | Excluded：review |
| 352 | Gillibrand S, Ntani G, Coggon D. Do exposure limits for hand-transmitted vibration prevent carpal tunnel syndrome? Occup Med (Lond). 2016 Jul;66(5):399-402. doi: 10.1093/occmed/kqw025. Epub 2016 May 9. PMID: 27162133; PMCID: PMC4913367. | Excluded：review |
| 353 | Sands WA, McNeal JR, Stone MH, Russell EM, Jemni M. Flexibility enhancement with vibration: Acute and long-term. Med Sci Sports Exerc. 2006 Apr;38(4):720-5. doi: 10.1249/01.mss.0000210204.10200.dc. PMID: 16679989. | Excluded：review |
| 354 | Cheng HY, Ju YY, Chen CL, Chuang LL, Cheng CH. Effects of whole body vibration on spasticity and lower extremity function in children with cerebral palsy. Hum Mov Sci. 2015 Feb;39:65-72. doi: 10.1016/j.humov.2014.11.003. Epub 2014 Nov 24. PMID: 25461434. | Excluded：review |
| 355 | Christie AD, Miller NR. Tndon vibration does not alter recovery time following fatigue. Med Sci Sports Exerc. 2015 May;47(5):931-6. doi: 10.1249/MSS.0000000000000492. PMID: 25202851. | Excluded：review |
| 356 | Uhlrich SD, Silder A, Beaupre GS, Shull PB, Delp SL. Subject-specific toe- in or toe-out gait modifications reduce the larger knee adduction moment peak more than a non-personalized approach. J Biomech. 2018 Jan 3;66:103-110. doi:10.1016/j.jbiomech.2017.11.003. Epub 2017 Nov 8. PMID: 29174534; PMCID: PMC5859947. | Excluded：review |
| 357 | Kiiski J, Heinonen A, Järvinen TL, Kannus P, Sievänen H. Transmission of vertical whole body vibration to the human body. J Bone Miner Res. 2008 Aug;23(8):1318-25. doi: 10.1359/jbmr.080315. PMID: 18348698. | Excluded：review |
| 358 | Yang F, Butler AJ. Efficacy of Controlled Whole-Body Vibration Training on Improving Fall Risk Factors in Stroke Survivors: A Meta-analysis. Neurorehabil Neural Repair. 2020 Apr;34(4):275-288. doi: 10.1177/1545968320907073. Epub 2020 Feb 28. PMID: 32106762. | Excluded：review |
| 359 | Bressel E, Smith G, Branscomb J. Transmission of whole body vibration in children while standing. Clin Biomech (Bristol, Avon). 2010 Feb;25(2):181-6. doi: 10.1016/j.clinbiomech.2009.10.016. Epub 2009 Nov 26. PMID: 19944501. | Excluded：review |
| 360 | Switlick T, Kernozek TW, Meardon S. Differences in joint-position sense and vibratory threshold in runners with and without a history of overuse injury. J Sport Rehabil. 2015 Feb;24(1):6-12. doi: 10.1123/jsr.2013-0089. Epub 2014 Jun  23. PMID: 24960029. | Excluded：review |
| 361 | Belavý DL, Wilson SJ, Armbrecht G, Rittweger J, Felsenberg D, Richardson CA. Resistive vibration exercise during bed-rest reduces motor control changes in the lumbo-pelvic musculature. J Electromyogr Kinesiol. 2012 Feb;22(1):21-30. doi: 10.1016/j.jelekin.2011.09.009. Epub 2011 Oct 20. PMID: 22018458. | Excluded：review |
| 362 | He L, Van Roie E, Bogaerts A, Morse CI, Delecluse C, Verschueren S, Thomis M. Genetic predisposition score predicts the increases of knee strength and muscle mass after one-year exercise in healthy elderly. Exp Gerontol. 2018 Oct 1;111:17-26. doi: 10.1016/j.exger.2018.06.030. Epub 2018 Jul 4. PMID: 29991458. | Excluded：review |
| 363 | otosy de Zepetnek JO, Miyatani M, Szeto M, Giangregorio LM, Craven BC. The effects of whole body vibration on pulse wave velocity in men with chronic spinal cord injury. J Spinal Cord Med. 2017 Nov;40(6):795-802. doi:0.1080/10790268.2017.1369248. Epub 2017 Sep 4. PMID: 28868990; PMCID:PMC5778943. | Excluded：review |
| 364 | Amonette WE, Boyle M, Psarakis MB, Barker J, Dupler TL, Ott SD.Neurocognitive responses to a single session of static squats with whole body vibration. J Strength Cond Res. 2015 Jan;29(1):96-100. doi: 10.1519/JSC.0b013e31829b26ce. PMID: 25536489. | Excluded：review |
| 365 | Shanahan CJ, Wrigley TV, Farrell MJ, Bennell KL, Hodges PW. Postural response to vibration of triceps surae, but not quadriceps muscles, differs between people with and without knee osteoarthritis. J Orthop Res. 2014 Aug;32(8):989-96. doi: 10.1002/jor.22637. Epub 2014 May 5. PMID: 24797419. | Excluded：review |
| 366 | Mileva KN, Naleem AA, Biswas SK, Marwood S, Bowtell JL. Acute effects of a vibration-like stimulus during knee extension exercise. Med Sci Sports Exerc. 2006 Jul;38(7):1317-28. doi: 10.1249/01.mss.0000227318.39094.b6. PMID: 16826030. | Excluded：review |
| 367 | Fernandez-Rio J, Terrados N, Fernandez-Garcia B, Suman OE. Effects of vibration training on force production in female basketball players. J Strength Cond Res. 2010 May;24(5):1373-80. doi: 10.1519/JSC.0b013e3181d1d2b1. PMID:  20386478. | Excluded：review |
| 368 | Yoshizuka H, Sato T, Murakami J, Mitsutake T, Hiromatsu M. Short-term changes in radiographic joint space width after jiggling exercise as conservative treatment for hip osteoarthritis: A retrospective case series ofnine patients. PLoS One. 2021 Jun 22;16(6):e0253643. doi: 10.1371/journal.pone.0253643. PMID: 34157042; PMCID: PMC8219140. |  |
| 369 | Pollock RD, Provan S, Martin FC, Newham DJ. The effects of whole body vibration on balance, joint position sense and cutaneous sensation. Eur J Appl Physiol. 2011 Dec;111(12):3069-77. doi: 10.1007/s00421-011-1943-y. Epub 2011 Apr 1. PMID: 21455611. | Excluded：review |
| 370 | Jordan M, Norris S, Smith D, Herzog W. Acute effects of whole-body vibration on peak isometric torque, muscle twitch torque and voluntary muscle activation of the knee extensors. Scand J Med Sci Sports. 2010 Jun;20(3):535-40.  doi: 10.1111/j.1600-0838.2009.00973.x. Epub 2009 Jul 3. PMID: 19602188. | Excluded：review |
| 371 | Cai S, Yang S, Zheng F, Lu M, Wu Y, Krishnan S. Knee joint vibration signal analysis with matching pursuit decomposition and dynamic weighted classifier fusion. Comput Math Methods Med. 2013;2013:904267. doi: 10.1155/2013/904267. Epub 2013 Mar 12. PMID: 23573175; PMCID: PMC3610364. | Excluded：review |
| 372 | Thorlund JB, Shakoor N, Ageberg E, Sandal LF, Block JA, Roos EM. Vibratory perception threshold in young and middle-aged patients at high risk of knee osteoarthritis compared to controls. Arthritis Care Res (Hoboken). 2012  Jan;64(1):144-8. doi: 10.1002/acr.20624. PMID: 21905255. | Excluded：review |
| 373 | Farkas GJ, Shakoor N, Cvetanovich GL, Fogg LF, Orías AAE, Nho SJ. Vibratory sense deficits in patients with symptomatic femoroacetabular impingement. J Musculoskelet Neuronal Interact. 2016 Mar;16(1):40-4. PMID: 26944822; PMCID: PMC5089454. | Excluded：review |
| 374 | Fuller JT, Thomson RL, Howe PR, Buckley JD. Vibration Therapy Is No More Effective Than the Standard Practice of Massage and Stretching for Promoting Recovery From Muscle Damage After Eccentric Exercise. Clin J Sport Med. 2015 Jul;25(4):332-7. doi: 10.1097/JSM.0000000000000149. PMID: 25290104. | Excluded：review |
| 375 | Kang SR, Kim GW, Ko MH, Han KS, Kwon TK. The effect of exercise load deviations in whole body vibration on improving muscle strength imbalance in the lower limb. Technol Health Care. 2020;28(S1):103-114. doi: 10.3233/THC-20900. PMID: 32333568; PMCID: PMC7369121. | Excluded：review |
| 376 | Chang SH, Dudley-Javoroski S, Shields RK. Gravitational force modulates muscle activity during mechanical oscillation of the tibia in humans. J Electromyogr Kinesiol. 2011 Oct;21(5):847-53. doi: 10.1016/j.jelekin.2011.06.001. Epub 2011 Jun 25. PMID: 21708472; PMCID: PMC3355375. | Excluded：review |
| 377 | Konishi Y, Yoshii R, Takeshita D. Tactile stimulation restores inhibited stretch reflex attributable to attenuation of Ia afferents during surprise landing. Scand J Med Sci Sports. 2024 Jan;34(1):e14568. doi: 10.1111/sms.14568.  PMID: 38268071. | Excluded：review |
| 378 | Osawa Y, Oguma Y. Effects of combining whole-body vibration with exercise on the consequences of detraining on muscle performance in untrained adults. J Strength Cond Res. 2013 Apr;27(4):1074-82. doi: 10.1519/JSC.0b013e31826520af. PMID: 22739330. | Excluded：review |
| 379 | Lovell R, Midgley A, Barrett S, Carter D, Small K. Effects of different half-time strategies on second half soccer-specific speed, power and dynamic strength. Scand J Med Sci Sports. 2013 Feb;23(1):105-13. doi: 10.1111/j.1600-0838.2011.01353.x. Epub 2011 Aug 3. PMID: 21812822. | Excluded：review |
| 380 | Alizadeh-Meghrazi M, Masani K, Zariffa J, Sayenko DG, Popovic MR, Craven BC. Effect of whole-body vibration on lower-limb EMG activity in subjects with and without spinal cord injury. J Spinal Cord Med. 2014 Sep;37(5):525-36. doi: 10.1179/2045772314Y.0000000242. Epub 2014 Jul 1. PMID: 24986541; PMCID: PMC4166187. | Excluded：review |
| 381 | Cronström A, Ageberg E. Association between sensory function and medio-lateral knee position during functional tasks in patients with anterior cruciate ligament injury. BMC Musculoskelet Disord. 2014 Dec 13;15:430. doi:  10.1186/1471-2474-15-430. PMID: 25494866; PMCID: PMC4301659. | Excluded：review |
| 382 | Gomez S, Patel M, Magnusson M, Johansson L, Einarsson EJ, Fransson PA. Differences between body movement adaptation to calf and neck muscle vibratory proprioceptive stimulation. Gait Posture. 2009 Jul;30(1):93-9. doi: 10.1016/j.gaitpost.2009.03.009. Epub 2009 Apr 23. PMID: 19398340. | Excluded：review |
| 383 | Rice D, Lewis G, McNair P. Impaired Regulation of Submaximal Force after ACL Reconstruction: Role of Muscle Spindles. Int J Sports Med. 2021 Jun;42(6):550-558. doi: 10.1055/a-1292-4461. Epub 2020 Nov 11. PMID: 33176382. | Excluded：review |
| 384 | Selionov VA, Ivanenko YP, Solopova IA, Gurfinkel VS. Tonic central and sensory stimuli facilitate involuntary air-stepping in humans. J Neurophysiol. 2009 Jun;101(6):2847-58. doi: 10.1152/jn.90895.2008. Epub 2009 Apr 1. PMID:19339461. | Excluded：review |
| 385 | Fry A, Folland JP. Prolonged infrapatellar tendon vibration does not influence quadriceps maximal or explosive isometric force production in man. Eur J Appl Physiol. 2014 Aug;114(8):1757-66. doi: 10.1007/s00421-014-2904-z. Epub 2014 May 21. PMID: 24846679. | Excluded：review |
| 386 | Saito A, Ando R, Akima H. Effects of prolonged patellar tendon vibration on force steadiness in quadriceps femoris during force-matching task. Exp Brain Res. 2016 Jan;234(1):209-17. doi: 10.1007/s00221-015-4447-x. Epub 2015 Sep 29. PMID: 26419664. | Excluded：review |
| 387 | Kelly SB, Alvar BA, Black LE, Dodd DJ, Carothers KF, Brown LE. The effect of warm-up with whole-body vibration vs. cycle ergometry on isokinetic dynamometry. J Strength Cond Res. 2010 Nov;24(11):3140-3. doi: 10.1519/JSC.0b013e3181f9278f. PMID: 20940645. | Excluded：review |
| 388 | Kamei N, Yamane K, Nakanishi S, Yamashita Y, Tamura T, Ohshita K, Watanabe H, Fujikawa R, Okubo M, Kohno N. Effectiveness of Semmes-Weinstein monofilament examination for diabetic peripheral neuropathy screening. J Diabetes Complications. 2005 Jan-Feb;19(1):47-53. doi: 10.1016/j.jdiacomp.2003.12.006. PMID: 15642490. | Excluded：review |
| 389 | Nawayseh N, Hamdan S, Bernardo-Filho M, Taiar R. Modelling the apparent mass of the standing human body under whole-body vibration training conditions. Proc Inst Mech Eng H. 2020 Jul;234(7):697-710. doi: 10.1177/0954411920917311. Epub 2020 May 6. PMID: 32370665. | Excluded：review |
| 390 | Tihanyi J, Di Giminiani R, Tihanyi T, Gyulai G, Trzaskoma L, Horváth M. Low resonance frequency vibration affects strength of paretic and non-paretic leg differently in patients with stroke. Acta Physiol Hung. 2010 Jun;97(2):172-82.  doi: 10.1556/APhysiol.97.2010.2.3. PMID: 20511126. | Excluded：review |
| 391 | Field-Fote E, Ness LL, Ionno M. Vibration elicits involuntary, step-like behavior in individuals with spinal cord injury. Neurorehabil Neural Repair. 2012 Sep;26(7):861-9. doi: 10.1177/1545968311433603. Epub 2012 Feb 9. PMID: 22328683. | Excluded：review |
| 392 | Bily W, Franz C, Trimmel L, Kasche W, Kern H, Loefler S, Zampieri S, Sarabon N, Cvecka J, Zenz P. The Authors Respond. Arch Phys Med Rehabil. 2016 Nov;97(11):2018-2019. doi: 10.1016/j.apmr.2016.08.458. PMID: 27780527. | Excluded：authors Respond |
| 393 | Camacho-Cardenosa M, Camacho-Cardenosa A, Brazo-Sayavera J, Olcina G, Tomas-Carus P, Timón R. Evaluation of 18-Week Whole-Body Vibration Training in Normobaric Hypoxia on Lower Extremity Muscle Strength in an Elderly Population. High Alt Med Biol. 2019 Jun;20(2):157-164. doi: 10.1089/ham.2018.0129. Epub 2019 Apr 25. PMID: 31021265. | Excluded：review |
| 394 | Shakoor N, Agrawal A, Block JA. Reduced lower extremity vibratory perception in osteoarthritis of the knee. Arthritis Rheum. 2008 Jan 15;59(1):117-21. doi: 10.1002/art.23241. PMID: 18163397; PMCID: PMC3653289. | Excluded：review |
| 395 | Annino G, Padua E, Castagna C, Di Salvo V, Minichella S, Tsarpela O, Manzi V, D'Ottavio S. Effect of whole body vibration training on lower limb performance in selected high-level ballet students. J Strength Cond Res. 2007 Nov;21(4):1072-6. doi: 10.1519/R-18595.1. PMID: 18076222. | Excluded：review |
| 396 | Savelberg HH, Keizer HA, Meijer K. Whole-body vibration induced adaptation in knee extensors; consequences of initial strength, vibration frequency, and joint angle. J Strength Cond Res. 2007 May;21(2):589-93. doi: 10.1519/R-20766.1. PMID: 17530984. | Excluded：review |
| 397 | Layne CS, Chelette AM, Pourmoghaddam A. Impact of altered lower limb proprioception produced by tendon vibration on adaptation to split-belt treadmill walking. Somatosens Mot Res. 2015;32(1):31-8. doi: 10.3109/08990220.2014.949007. Epub 2014 Aug 27. PMID: 25162146. | Excluded：review |
| 398 | Hadi SC, Delparte JJ, Hitzig SL, Craven BC. Subjective experiences of men with and without spinal cord injury: tolerability of the juvent and WAVE whole body vibration plates. PM R. 2012 Dec;4(12):954-62. doi: 10.1016/j.pmrj.2012.07.006. Epub 2012 Sep 12. PMID: 22981004. | Excluded：review |
| 399 | Jacobs PL, Burns P. Acute enhancement of lower-extremity dynamic strength and flexibility with whole-body vibration. J Strength Cond Res. 2009 Jan;23(1):51-7. doi: 10.1519/JSC.0b013e3181839f19. PMID: 18824930. | Excluded：review |
| 400 | Medel FJ, Rimnac CM, Kurtz SM. On the assessment of oxidative and microstructural changes after in vivo degradation of historical UHMWPE knee components by means of vibrational spectroscopies and nanoindentation. J Biomed Mater Res A. 2009 May;89(2):530-8. doi: 10.1002/jbm.a.31992. PMID: 18435407; PMCID: PMC2661358. | Excluded：review |
| 401 | Li X, Liu Z, Gao X, Zhang J. Bicycling Phase Recognition for Lower Limb Amputees Using Support Vector Machine Optimized by Particle Swarm Optimization. Sensors (Basel). 2020 Nov 15;20(22):6533. doi: 10.3390/s20226533. PMID: 33203169; PMCID: PMC7696493. | Excluded：review |
| 402 | Munera M, Bertucci W, Duc S, Chiementin X. Analysis of muscular activity and dynamic response of the lower limb adding vibration to cycling. J Sports Sci. 2018 Jul;36(13):1465-1475. doi: 10.1080/02640414.2017.1398407. Epub 2017 Nov 3. PMID: 29099665. | Excluded：review |
| 403 | Shull PB, Zhu X, Cutkosky MR. Continuous Movement Tracking Performance for Predictable and Unpredictable Tasks with Vibrotactile Feedback. IEEE Trans Haptics. 2017 Oct-Dec;10(4):466-475. doi: 10.1109/TOH.2017.2689023. Epub 2017 Mar 29. PMID: 28368831. | Excluded：review |
| 404 | Plater EB, Sharma T, Peters RM, Bent LR. Calf cutaneous stimulation generates reflexes in the thigh that can be modified by subthreshold noisy vibration. J Neurophysiol. 2023 Jul 1;130(1):199-211. doi: 10.1152/jn.00497.2022. Epub 2023 Jun 28. PMID: 37377219. | Excluded：review |
| 405 | Tsai ST, Li CF, Chi KC, Ko LW, Stevenson C, Chen YJ, Chen CH. Immediate Effect of Whole Body Vibration on Knee Extensor Tendon Stiffness in Hemiparetic Stroke Patients. Medicina (Kaunas). 2021 Sep 29;57(10):1037. doi:10.3390/medicina57101037. PMID: 34684074; PMCID: PMC8540205. | Excluded: the inclusion criteria were not met after reading the title and were therefore excluded |
| 406 | Wu Z, Zou Z, Zhong J, Fu X, Yu L, Wang J, Wang X, Wu Q, Hou X. Effects of whole-body vibration plus hip-knee muscle strengthening training on adult patellofemoral pain syndrome: a randomized controlled trial. Disabil Rehabil.  2022 Oct;44(20):6017-6025. doi: 10.1080/09638288.2021.1954703. Epub 2021 Aug 4.PMID: 34346273. | Excluded: the inclusion criteria were not met after reading the abstract and were therefore excluded |
| 407 | Moezy A, Olyaei G, Hadian M, Razi M, Faghihzadeh S. A comparative study of whole body vibration training and conventional training on knee proprioception and postural stability after anterior cruciate ligament reconstruction. Br J Sports Med. 2008 May;42(5):373-8. doi: 10.1136/bjsm.2007.038554. Epub 2008 Jan 8. PMID: 18182623. | Excluded: the inclusion criteria were not met after reading the abstract and were therefore excluded |
| 408 | Befrui N, Elsner J, Flesser A, Huvanandana J, Jarrousse O, Le TN, Müller M, Schulze WHW, Taing S, Weidert S. Vibroarthrography for early detection of knee osteoarthritis using normalized frequency features. Med Biol Eng Comput. 2018 Aug;56(8):1499-1514. doi: 10.1007/s11517-018-1785-4. Epub 2018 Feb 1. PMID: 29392547. | Excluded: the inclusion criteria were not met after reading the abstract and were therefore excluded |
| 409 | Rieder F, Wiesinger HP, Kösters A, Müller E, Seynnes OR. Immediate effects of whole body vibration on patellar tendon properties and knee extension torque. Eur J Appl Physiol. 2016 Mar;116(3):553-61. doi: 10.1007/s00421-015-3316-4. Epub 2015 Dec 26. PMID: 26708361. | Excluded: the inclusion criteria were not met after reading the abstract and were therefore excluded |
| 410 | Pasterczyk-Szczurek A, Golec J, Golec E. Effect of low-magnitude, variable-frequency vibration therapy on pain threshold levels and mobility in adults with moderate knee osteoarthritis-randomized controlled trial. BMC Musculoskelet Disord. 2023 Apr 13;24(1):287. doi: 10.1186/s12891-023-06334-9. PMID: 37055733; PMCID: PMC10099927. | Excluded: the inclusion criteria were not met after reading the abstract and were therefore excluded |
| 411 | Coelho-Oliveira AC, Lacerda ACR, de Souza ALC, Santos LMM, da Fonseca SF, Dos Santos JM, Ribeiro VGC, Leite HR, Figueiredo PHS, Fernandes JSC, Martins F, Filho RGT, Bernardo-Filho M, da Cunha de Sá-Caputo D, Sartorio A, Cochrane D, Lima VP, Costa HS, Mendonça VA, Taiar R. Acute Whole-Body Vibration Exercise Promotes Favorable Handgrip Neuromuscular Modifications in Rheumatoid Arthritis: A Cross-Over Randomized Clinical. Biomed Res Int. 2021 Dec 2;2021:9774980. doi: 10.1155/2021/9774980. PMID: 34901282; PMCID: PMC8660187. | Excluded: the inclusion criteria were not met after reading the abstract and were therefore excluded |
| 412 | Artero EG, Espada-Fuentes JC, Argüelles-Cienfuegos J, Román A, Gómez-López PJ, Gutiérrez A. Effects of whole-body vibration and resistance training on knee extensors muscular performance. Eur J Appl Physiol. 2012 Apr;112(4):1371-8. doi: 10.1007/s00421-011-2091-0. Epub 2011 Aug 2. PMID: 21809090. | Excluded: the inclusion criteria were not met after reading the abstract and were therefore excluded |
| 413 | Barati K, Kamyab M, Takamjani IE, Bidari S, Parnianpour M. Effect of equipping an unloader knee orthosis with vibrators on pain, function, stiffness, and knee adduction moment in people with knee osteoarthritis: A pilot randomized trial. Gait Posture. 2023 Jan;99:83-89. doi: 10.1016/j.gaitpost.2022.10.019. Epub 2022 Oct 29. PMID: 36368240. | Excluded: the inclusion criteria were not met after reading the abstract and were therefore excluded |
| 414 | Sañudo B, Feria A, Carrasco L, de Hoyo M, Santos R, Gamboa H. Does whole body vibration training affect knee kinematics and neuromuscular control in healthy people? J Sports Sci. 2012;30(14):1537-44. doi: 10.1080/02640414.2012.713503. Epub 2012 Aug 16. PMID: 22894146. | Excluded: the inclusion criteria were not met after reading the abstract and were therefore excluded |
| 415 | Tossige-Gomes R, Avelar NC, Simão AP, Neves CD, Brito-Melo GE, Coimbra CC, Rocha-Vieira E, Lacerda AC. Whole-body vibration decreases the proliferativeb response of TCD4(+) cells in elderly individuals with knee osteoarthritis. Braz J Med Biol Res. 2012 Dec;45(12):1262-8. doi: 10.1590/s0100-879x2012007500139. Epub 2012 Sep 6. PMID: 22948377; PMCID: PMC3854226. | Excluded: the inclusion criteria were not met after reading the abstract and were therefore excluded |
| 416 | Huang M, Tang CY, Pang MYC. Use of whole body vibration in individuals with chronic stroke: Transmissibility and signal purity. J Biomech. 2018 May  17;73:80-91. doi: 10.1016/j.jbiomech.2018.03.022. Epub 2018 Mar 17. PMID:29588022. | Excluded: the inclusion criteria were not met after reading the title and were therefore excluded |
| 417 | He Z, Zheng J, Liu S, Guan Z, Zhou Q, Jin X, Guan Z. The effect of whole-body vibration in osteopenic patients after total knee arthroplasty: a randomized controlled trial. Aging Clin Exp Res. 2022 Jun;34(6):1381-1390. doi: 10.1007/s40520-021-02043-2. Epub 2022 Jan 14. PMID: 35028919. | Excluded: the inclusion criteria were not met after reading the abstract and were therefore excluded |
| 418 | Blackburn JT, Pamukoff DN, Sakr M, Vaughan AJ, Berkoff DJ. Whole body and local muscle vibration reduce artificially induced quadriceps arthrogenic inhibition. Arch Phys Med Rehabil. 2014 Nov;95(11):2021-8. doi: 10.1016/j.apmr.2014.07.393. Epub 2014 Jul 30. PMID: 25083559. | Excluded: the inclusion criteria were not met after reading the abstract and were therefore excluded |
| 419 | Krause A, Gollhofer A, Lee K, Freyler K, Becker T, Kurz A, Ritzmann R. Acute whole-body vibration reduces post-activation depression in the triceps surae muscle. Hum Mov Sci. 2020 Aug;72:102655. doi: 10.1016/j.humov.2020.102655. Epub 2020 Jul 9. PMID: 32721374. | Excluded: the inclusion criteria were not met after reading the abstract and were therefore excluded |
| 420 | Fu CL, Yung SH, Law KY, Leung KH, Lui PY, Siu HK, Chan KM. The effect of early whole-body vibration therapy on neuromuscular control after anterior cruciate ligament reconstruction: a randomized controlled trial. Am J Sports  Med. 2013 Apr;41(4):804-14. doi: 10.1177/0363546513476473. Epub 2013 Mar 4. PMID: 23460328. | Excluded: the inclusion criteria were not met after reading the abstract and were therefore excluded |
| 421 | Stein, G., Knoell, P., Faymonville, C., Kaulhausen, T., Siewe, J., Otto, C., Eysel, P., & Zarghooni, K. (2010). Whole body vibration compared to conventional physiotherapy in patients with gonarthrosis: a protocol for a randomized, controlled study. BMC musculoskeletal disorders, 11, 128. https://doi.org/10.1186/1471-2474-11-128 | Excluded: study protocols (no results available) |
| 422 | Kurtça, M. P., Aslan, U. B., Koçyiğit, F., Koçyiğit, A., & Kuyucu, E. (2016). Effects of lower limb exercise training with whole body vibration on femoral articular cartilage in patients with knee osteoarthritis. Osteoarthritis and Cartilage, 24, S496. | Excluded: Not a randomized controlled trial; only single-group experimentation with pre-post assessments. |
| 423 | Moura-Fernandes, M. C., Moreira-Marconi, E., de Meirelles, A. G., Reis-Silva, A., de Souza, L. F. F., Lirio Pereira da Silva, A., ... & Bernardo-Filho, M. (2020). Acute effects of whole-body vibration exercise on pain level, functionality, and rating of exertion of elderly obese knee osteoarthritis individuals: a randomized study. Applied Sciences, 10(17), 5870. | Excluded: The study lacked a proper control group, as the control group did not receive rehabilitation exercises. |
| 424 | Zhang, J., Wang, R., Zheng, Y., Xu, J., Wu, Y., & Wang, X. (2021). Effect of Whole-Body Vibration Training on Muscle Activation for Individuals with Knee Osteoarthritis. BioMed research international, 2021, 6671390. https://doi.org/10.1155/2021/6671390 | Excluded: This is a cross-sectional study, not a randomized controlled trial; only single-group experimentation with pre-post assessments. |
| 425 | Yu, X., Guo, J., & Jihua, Y. (2018). Effects of different vibration modes on proprioception and motor function in female patients with knee osteoarthritis [J]. Chinese Journal of Rehabilitation Medicine, 33(8), 940-944. | Excluded: intervention applied in this trial without combination with rehabilitation exercise |
| 426 | Moreira-Marconi, E., Dionelo, C. F., Sa-Caputo, D. C., Paineiras-Domingos, L. L., Souza-Goncalves, C. R., & Bernardo-Filho, M. (2018, April). Whole body vibration increase functionality in individuals with knee osteoartrhitis. In osteoporosis international (vol. 29, pp. S523-s524). | Excluded: intervention applied in this trial without combination with rehabilitation exercise |
| 427 | Osugi, T., Iwamoto, J., Yamazaki, M., & Takakuwa, M. (2014). Effect of a combination of whole body vibration exercise and squat training on body balance, muscle power, and walking ability in the elderly. Therapeutics and clinical risk management, 10, 131–138. https://doi.org/10.2147/TCRM.S57806 | Excluded: intervention applied in this trial without combination with rehabilitation exercise; not specific for KOA population |
| 428 | Trans, T., Aaboe, J., Henriksen, M., Christensen, R., Bliddal, H., & Lund, H. (2009). Effect of whole body vibration exercise on muscle strength and proprioception in females with knee osteoarthritis. The Knee, 16(4), 256–261. https://doi.org/10.1016/j.knee.2008.11.014 | Excluded: intervention applied in this trial without combination with rehabilitation exercise |
| 429 | Segal, N. A., Glass, N. A., Shakoor, N., & Wallace, R. (2013). Vibration platform training in women at risk for symptomatic knee osteoarthritis. PM & R : the journal of injury, function, and rehabilitation, 5(3), 201–209. https://doi.org/10.1016/j.pmrj.2012.07.011 | Excluded: intervention applied in this trial without combination with rehabilitation exercise |
| 430 | Lai, Z., Wang, X., Lee, S., Hou, X., & Wang, L. (2017). Effects of whole body vibration exercise on neuromuscular function for individuals with knee osteoarthritis: study protocol for a randomized controlled trial. Trials, 18(1), 437. https://doi.org/10.1186/s13063-017-2170-6 | Excluded: study protocols (no results available) |
| 431 | Bokaeian, H. R., Bakhtiary, A. H., Mirmohammadkhani, M., & Moghimi, J. (2016). The effect of adding whole body vibration training to strengthening training in the treatment of knee osteoarthritis: A randomized clinical trial. Journal of bodywork and movement therapies, 20(2), 334–340. https://doi.org/10.1016/j.jbmt.2015.08.005 | Included |
| 432 | Aggarwal A. Effect of whole body vibration on lower body strength and balance in osteoarthritis knee. Int J Physiother. 2020;7(2):7. | Included |
| 433 | Avelar, N. C., Simão, A. P., Tossige-Gomes, R., Neves, C. D., Rocha-Vieira, E., Coimbra, C. C., & Lacerda, A. C. (2011). The effect of adding whole-body vibration to squat training on the functional performance and self-report of disease status in elderly patients with knee osteoarthritis: a randomized, controlled clinical study. Journal of alternative and complementary medicine (New York, N.Y.), 17(12), 1149–1155. https://doi.org/10.1089/acm.2010.0782 | Included |
| 434 | Bokaeian, H. R., Bakhtiary, A. H., Mirmohammadkhani, M., & Moghimi, J. (2016). The effect of adding whole body vibration training to strengthening training in the treatment of knee osteoarthritis: A randomized clinical trial. Journal of bodywork and movement therapies, 20(2), 334–340. https://doi.org/10.1016/j.jbmt.2015.08.005 | Included |
| 435 | Lai, Z., Lee, S., Hu, X., & Wang, L. (2019). Effect of adding whole-body vibration training to squat training on physical function and muscle strength in individuals with knee osteoarthritis. Journal of musculoskeletal & neuronal interactions, 19(3), 333–341. | Included |
| 436 | Lai, Z., Lee, S., Chen, Y., & Wang, L. (2021). Comparison of whole-body vibration training and quadriceps strength training on physical function and neuromuscular function of individuals with knee osteoarthritis: A randomised clinical trial. Journal of exercise science and fitness, 19(3), 150–157. https://doi.org/10.1016/j.jesf.2021.01.003 | Included |
| 437 | ark, Y. G., Kwon, B. S., Park, J. W., Cha, D. Y., Nam, K. Y., Sim, K. B., Chang, J., & Lee, H. J. (2013). Therapeutic effect of whole body vibration on chronic knee osteoarthritis. Annals of rehabilitation medicine, 37(4), 505–515. https://doi.org/10.5535/arm.2013.37.4.505 | Included |
| 438 | Trans, T., Aaboe, J., Henriksen, M., Christensen, R., Bliddal, H., & Lund, H. (2009). Effect of whole body vibration exercise on muscle strength and proprioception in females with knee osteoarthritis. The Knee, 16(4), 256-261. | Included |
| 439 | Segal, N. A., Glass, N. A., Shakoor, N., & Wallace, R. (2013). Vibration platform training in women at risk for symptomatic knee osteoarthritis. PM & R : the journal of injury, function, and rehabilitation, 5(3), 201–209. https://doi.org/10.1016/j.pmrj.2012.07.011 | Included |
| 440 | Simão, A. P., Avelar, N. C., Tossige-Gomes, R., Neves, C. D., Mendonça, V. A., Miranda, A. S., Teixeira, M. M., Teixeira, A. L., Andrade, A. P., Coimbra, C. C., & Lacerda, A. C. (2012). Functional performance and inflammatory cytokines after squat exercises and whole-body vibration in elderly individuals with knee osteoarthritis. Archives of physical medicine and rehabilitation, 93(10), 1692–1700. https://doi.org/10.1016/j.apmr.2012.04.017 | Included |
| 441 | Simão, A. P., Mendonça, V. A., Avelar, N. C. P., da Fonseca, S. F., Santos, J. M., de Oliveira, A. C. C., Tossige-Gomes, R., Ribeiro, V. G. C., Neves, C. D. C., Balthazar, C. H., Leite, H. R., Figueiredo, P. H. S., Bernardo-Filho, M., & Lacerda, A. C. R. (2019). Whole Body Vibration Training on Muscle Strength and Brain-Derived Neurotrophic Factor Levels in Elderly Woman With Knee Osteoarthritis: A Randomized Clinical Trial Study. Frontiers in physiology, 10, 756. https://doi.org/10.3389/fphys.2019.00756 | Included |
| 442 | Tsuji, T., Yoon, J., Aiba, T., Kanamori, A., Okura, T., & Tanaka, K. (2014). Effects of whole-body vibration exercise on muscular strength and power, functional mobility and self-reported knee function in middle-aged and older Japanese women with knee pain. The Knee, 21(6), 1088–1095. https://doi.org/10.1016/j.knee.2014.07.015 | Included |
| 443 | Wang, P., Yang, L., Liu, C., Wei, X., Yang, X., Zhou, Y., Jiang, H., Lei, Z., Reinhardt, J. D., & He, C. (2016). Effects of Whole Body Vibration Exercise associated with Quadriceps Resistance Exercise on functioning and quality of life in patients with knee osteoarthritis: a randomized controlled trial. Clinical rehabilitation, 30(11), 1074–1087. https://doi.org/10.1177/0269215515607970 | Included |
| 444 | Wang, P., Yang, L., Li, H., Lei, Z., Yang, X., Liu, C., Jiang, H., Zhang, L., Zhou, Z., Reinhardt, J. D., & He, C. (2016). Effects of whole-body vibration training with quadriceps strengthening exercise on functioning and gait parameters in patients with medial compartment knee osteoarthritis: a randomised controlled preliminary study. Physiotherapy, 102(1), 86–92. https://doi.org/10.1016/j.physio.2015.03.3720 | Included |
| 445 | Xia X. The efficacy of whole-body vibration training combined with aerobic training in the treatment of knee osteoarthritis. Impact Res Med Appl. 2017;1(11):3. | Included |
| 446 | Zhang, J., Wang, R., Zheng, Y., Xu, J., Wu, Y., & Wang, X. (2021). Effect of Whole-Body Vibration Training on Muscle Activation for Individuals with Knee Osteoarthritis. BioMed research international, 2021, 6671390. https://doi.org/10.1155/2021/6671390 | Included |
