# Supplementary material for: Effects of whole-body vibration training as an adjunct to conventional rehabilitation exercise on pain, physical function and disability in knee osteoarthritis: A systematic review and meta-analysis
Source: PLoS One. 2025 Feb 10;20(2):e0318635. doi: 10.1371/journal.pone.0318635 (PMC11809854; doi:10.1371/journal.pone.0318635)
Supplement: S2 Table — (PDF) [file pone.0318635.s004.pdf]

S2_Table. Results of the PEDro Scale Evaluation

Study / Item	1	2		3	4	5	6	7	8	9	10	11	Total PEDro Score
Abbasi et al. (2017)	1	1		1	1	1	0	0	0	0	1	1	6
Aggarwal et al. (2020)	0	0		0	1	0	0	0	1	0	1	1	4
Avelar et al. (2011)	0	1		0	1	0	0	0	1	0	1	1	5
Bokaeian et al. (2016)	1	1		1	1	0	0	1	1	0	1	1	7
Lai et al. (2021)	1	1		1	1	0	0	1	0	1	1	1	7
Lai et al. (2019)	1	1		1	1	0	0	1	0	0	1	1	6
Park et al. (2013)	0	1		0	1	0	0	0	0	0	1	1	4
Wang et al. (2016)	1	1		1	1	0	1	1	1	0	1	1	8
Simão et al. (2012)	0	1		1	1	0	0	1	1	0	1	1	7
Tsuji et al. (2014)	0	0		0	1	0	0	0	0	1	1	1	4
Wang et al. (2015)	1	1		1	1	0	1	1	1	1	1	1	9
Simão et al. (2019)	1	1		1	1	1	0	1	1	0	1	1	8
Segal et al. (2019)	1	1		1	1	1	0	0	1	0	1	1	7
Xia et al. (2017)	1	1		0	1	1	0	0	1	0	1	1	6
Zhang et al. (2021)	0	1		0	1	0	0	0	1	0	1	1	5
Philip et al. (2018)	0	1		0	1	0	0	0	1	0	1	1	5

- 0 = No, 1 = Yes

Criterion 1 of the PEDro scale assesses external validity and is not included in the final quality score (total score out of 10).

PEDro Scale Items:

1. Eligibility criteria specified
2. Random allocation of subjects to interventions
3. Concealed allocation
4. Similar baseline characteristics across groups
5. Blinding of subjects
6. Blinding of therapists
7. Blinding of assessors
8. Outcome measures obtained from more than 85% of participants
9. Intention-to-treat analysis
10. Statistical comparison between intervention groups for key outcomes
11. Provision of both point measures and measures of variability for at least one key outcome
