# Supplementary material for: Effects of whole-body vibration training as an adjunct to conventional rehabilitation exercise on pain, physical function and disability in knee osteoarthritis: A systematic review and meta-analysis
Source: PLoS One. 2025 Feb 10;20(2):e0318635. doi: 10.1371/journal.pone.0318635 (PMC11809854; doi:10.1371/journal.pone.0318635)
Supplement: S3 Table — (PDF) [file pone.0318635.s005.pdf]

S3 Table. Cochrane risk-of-bias assessment details.

Study	Random sequence generation	Allocation concealment	Blinding (participants and personnel)	Blinding (outcome assessment)	Incomplete outcome data	Selective reporting
Abbasi et al. 2017	low risk	low risk	low risk	**high risk: outcome assessors were not blinded**	low risk	low risk
Aggarwal et al. 2020	low risk	**high risk: the allocation was not concealed**	**high risk: participants and personnel were not blinded**	unclear risk: does not mention whether outcome assessors were blinded	low risk	low risk
Avelar et al. 2011	low risk	**high risk: the allocation was not concealed**	**high risk: participants not blinded**	**high risk: outcome assessors were not blinded**	low risk	low risk

Bokaeian et al. 2016	low risk	low risk	**high risk: participants not blinded**	low risk	low risk	low risk
Lai et al. 2019	low risk	low risk	**high risk: participants not blinded**	low risk	**high risk: per-protocol analysis with significant dropout**	low risk
Lai et al. 2021	low risk	low risk	**high risk: participants not blinded**	low risk	low risk	low risk
Park et al. 2013	low risk	**high risk: the allocation was not concealed**	**high risk: participants not blinded**	**high risk: outcome assessors were not blinded**	low risk	low risk
Wang et al. 2015	low risk	low risk	low risk	low risk	low risk	low risk

Simao et al. 2012	low risk	low risk	**high risk: participants not blinded**	low risk	low risk	low risk
Tsuji et al. 2014	**high risk (not specified)**	**high risk: the allocation was not concealed**	unclear risk (single-blind, but no mention of who's blind)	unclear risk (single-blind, but no mention of who's blind)	low risk	unclear risk (this information is not available)
Wang et al. 2016	low risk	low risk	low risk	low risk	low risk	low risk
Simao et al. 2019	low risk	low risk	low risk	low risk	low risk	low risk
Segal et al. 2019	low risk	low risk	low risk	**high risk: outcome assessors were not blinded**	low risk	low risk

Xia et al. 2017	low risk	**unclear risk (method of allocation concealment was not described)**	low risk	**high risk: outcome assessors were not blinded**	low risk	low risk
Zhang et al. 2021	low risk	**unclear risk (method of allocation concealment was not described)**	unclear risk (single-blind, but no mention of who's blind)	unclear risk (single-blind, but no mention of who's blind)	low risk	low risk
Philip et al. 2018	low risk	**unclear risk (method of allocation concealment was not described)**	unclear risk (single-blind, but no mention of who's blind)	unclear risk (single-blind, but no mention of who's blind)	low risk	low risk
